# Mountain Refugia Play a Role in Soil Arthropod Speciation on Madagascar: A Case Study of the Endemic Giant Fire-Millipede Genus *Aphistogoniulus*

**DOI:** 10.1371/journal.pone.0028035

**Published:** 2011-12-06

**Authors:** Thomas Wesener, Michael J. Raupach, Peter Decker

**Affiliations:** 1 Zoological Research Museum Alexander Koenig, Bonn, Germany; 2 AG Molekulare Taxonomie Mariner Organismen, Deutsches Zentrum für Marine Biodiversitätsforschung, Senckenberg am Meer, Wilhelmshaven, Germany; 3 Metadatenbank Myriapoda, Bodenzoologie, Senckenberg Museum für Naturkunde Görlitz, Görlitz, Germany; Field Museum of Natural History, United States of America

## Abstract

To elucidate the speciation mechanisms prevalent within hotspots of biodiversity, and the evolutionary processes behind the rise of their species-rich and endemic biota, we investigated the phylogeny of the giant fire-millipede genus *Aphistogoniulus* Silvestri, 1897, a Malagasy endemic. This study is the first comprehensive (molecular and morphological) phylogenetic study focusing on millipede (class Diplopoda) speciation on Madagascar. The morphological analysis is based on 35 morphological characters and incorporates ten described as well as two newly described species (*A. rubrodorsalis* **n. sp.** and *A. jeekeli* **n. sp.**) of *Aphistogoniulus*. The molecular analysis is based on both mitochondrial (COI and 16S), and nuclear genes (complete 18S rDNA), together comprised of 3031 base pairs, which were successfully sequenced for 31 individual specimens and eight species of *Aphistogoniulus*. In addition to the null-model (speciation by distance), two diversification models, mountain refugia and ecotone shift, were discovered to play a role in the speciation of soil arthropods on Madagascar. Mountain refugia were important in the speciation of the *A. cowani* clade, with three species occurring in the Andringitra and Ranomafana Mountains in the southeast (*A. cowani*), the Ambohijanahary and Ambohitantely Mountains in the mid-west (*A. sanguineus*), and the Marojejy Mountain in the northeast (*A. rubrodorsalis* **n. sp.**). An ecotone shift from the eastern rainforest to the unique subarid spiny forest of Mahavelo was discovered in the *A. vampyrus* - *A. aridus* species-pair. In the monophyletic *A. diabolicus* clade, evidence for divergent evolution of sexual morphology was detected: species with greatly enlarged gonopods are sister-taxa to species with normal sized gonopods. Among the large-bodied Spirobolida genera of Madagascar, *Colossobolus* and *Sanguinobolus* were found to be close sister-genera to *Aphistogoniulus*. Forest destruction has caused forest corridors between populations to disappear, which might limit the possible resolution of biogeographic analyses on Madagascar.

## Introduction

Madagascar is one of the world's centers of endemism and is considered a biodiversity hotspot [1]; with its highly diverse ecosystems, it mirrors a small continent rather than an island. Although now located off the coast of eastern Africa, until 88 million years ago Madagascar formed a landmass with India [2]. The Madagascar-India landmass separated from continental Africa in the early Mesozoic era, 158–160 million years ago [3], although land bridges towards Antarctica might have existed up to a more recent date [4]. The long isolation of Madagascar from other continents resulted in mixed-origins of the Malagasy fauna: neo-endemics whose ancestors colonized the island and quickly diversified [5]–[7]; and truly ancient endemics, whose ancestors were present before Madagascar became an island [8]–[11].

The isolation of Madagascar makes the island an ideal model region for the study of diversification in species, because unlike on most continents, introgression or admixture events with lineages that evolved outside the area can be almost ruled out [12]. Recent studies, almost exclusively on vertebrates, present different hypotheses of diversification mechanisms on Madagascar (see overview in [12]), including mountain refugia [13], [14], retreat-dispersal watersheds [15], and river barriers [16]. Unfortunately, some speciation patterns might already be artificially modified due to habitat destructions by humans, especially in the transitional zones [17].

Currently, the study of speciation events on Madagascar focuses primarily on vertebrates (e. g. [14], [18]–[21]). Although terrestrial invertebrates represent the largest percentage of Madagascar's diversity, little is known about their biogeography. The existing studies of Madagascar's invertebrates focus on taxa more likely to be dispersers, either through their ability to fly [6], [22], [23] or to wind disperse [24]. Non-flying soil invertebrates are, with some notable exceptions in mollusks [25], [26], generally ignored. For Madagascar, the general absence of studies of taxa with few dispersal abilities is problematic because, unlike in mobile taxa, certain dispersal mechanisms can be ruled out, which provides a clearer view of the past [27]. Many soil invertebrates are good model taxa for biogeographic studies because they possess poor dispersal abilities, are often confined to now discontinuous habitats, and show slow growth and low fecundity [28]. For example, in the Australian wet tropics non-flying invertebrates have a much greater number of subregional endemics compared to vertebrates (50% vs. 9%, [29]). Furthermore, invertebrates have smaller habitat requirements than vertebrates [30], and are more likely to have a continuous history of occupation on a site than a mammal species [27].

The incorporation of less vagile lineages into the studies of speciation events on Madagascar may be useful because they are phylogenetically older, can survive in smaller habitats during climatically unfavorable times, and might represent relictual elements, living witnesses of a climatically different past.

This study utilizes the giant fire-millipede genus *Aphistogoniulus* Silvestri, 1897 as a test case for invertebrate speciation on Madagascar. As in other groups of organisms [31], species representing the largest, most widespread and most colorful Malagasy millipede genus were described first. For the Spirobolida from Madagascar, this was the large-bodied fire-millipede genus *Aphistogoniulus* [32], with its first species being described almost 130 years ago [33]. Species of *Aphistogoniulus* feature a striking pitchblack/blood-red aposematic color, sometimes accompanied by golden legs, and can reach a body length of up to 185 mm. A complete taxonomic revision of *Aphistogoniulus* was recently undertaken [34], providing a foundation for the phylogenetic study undertaken here. The wide distribution of the genus renders *Aphistogoniulus* a suitable model taxon to test different methods of species diversification on Madagascar (as proposed by [12]). Species of the genus can be found in montane and lowland rainforests all the way from the southeastern tip of the island, expanding north, leaving out only the far north of Madagascar [34]. Two species almost never live in sympatry, but replace each other in adjacent areas. Only a single species, *A. aridus* Wesener, 2009, lives in the dry spiny forest.

Although a complete revision of the genus *Aphistogoniulus* was undertaken recently, two additional undescribed species were discovered in more or less recent collections, including the pet trade. These new discoveries prompted the attempts undertaken in this study to construct a phylogeny of all species of the genus *Aphistogoniulus*, in combination with all other large-bodied Spirobolida genera from Madagascar. This study is one of only a handful of molecular systematic studies of millipedes [35] and the first dealing with millipedes in the southern hemisphere. It is investigated which diversification mechanisms, such as mountain refugia [13] or retreat-dispersion watersheds [15], most-likely played a role in the speciation of the endemic rainforest soil biome on Madagascar.

## Materials and Methods

### Nomenclatural Acts

The electronic version of this document does not represent a published work according to the International Code of Zoological Nomenclature (ICZN), and hence the nomenclatural acts contained in the electronic version are not available under that Code from the electronic edition. Therefore, a separate edition of this document was produced by a method that assures numerous identical and durable copies, and those copies were simultaneously obtainable (from the publication date noted on the first page of this article) for the purpose of providing a public and permanent scientific record, in accordance with Article 8.1 of the Code. The separate print-only edition is available on request from PLoS by sending a request to PLoS ONE, Public Library of Science, 1160 Battery Street, Suite 100, San Francisco, CA 94111, USA along with a check for $10 (to cover printing and postage) payable to “Public Library of Science”. In addition, this published work and the nomenclatural acts it contains have been registered in ZooBank, the proposed online registration system for the ICZN. The ZooBank LSIDs (Life Science Identifiers) can be resolved and the associated information viewed through any standard web browser by appending the LSID to the prefix “http://zoobank.org/”. The LSID for this publication is: urn:lsid:zoobank.org:pub:BE53B87F-9FDA-4B75-B461-A7110D373A99; urn:lsid:zoobank.org:pub:BE53B87F-9FDA-4B75-B461-A7110D373A99.

### Morphological Phylogenetic Analysis

#### Taxon selection

**Figure 1:**
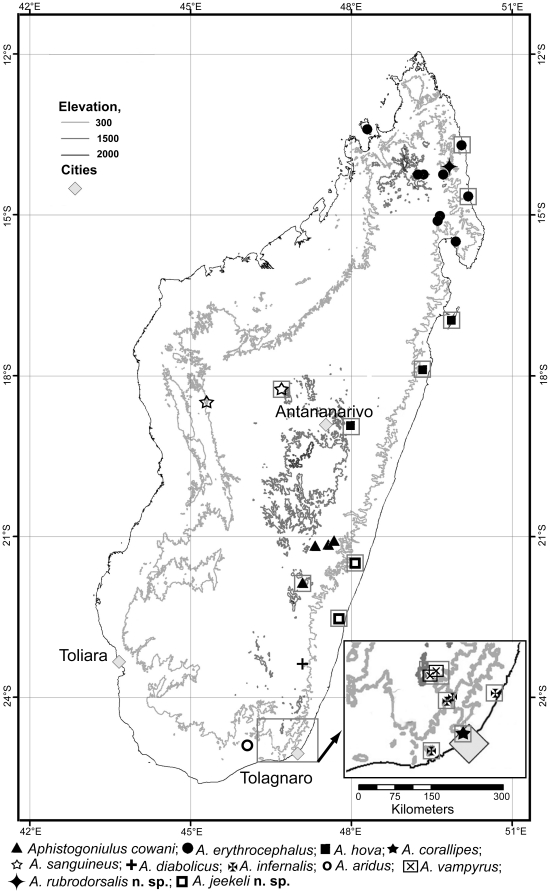
Distribution map of the genus *Aphistogoniulus*. New species described are already added. New locality information given in Supporting Information S1. Boxes surround localities from which DNA could be extracted from specimens.

Because the closest relative of *Aphistogoniulus* is unknown, several genera were selected as outgroups. The distant outgroup includes *Madabolus maximus* Wesener & Enghoff, 2008 from Madagascar and *Epibolus pulchripes* from continental Africa, members of the tribe Pachybolini, which are not closely related to *Aphistogoniulus* [36]. The near-outgroup includes members of all other large-bodied Spirobolida genera from Madagascar [37], the monotypic *Corallobolus* Wesener, 2009 and *Sanguinobolus* Wesener, 2009, as well as two species of the genus *Colossobolus*, *C. semicyclus* Wesener, 2009 and *C. oblongopedus* Wesener, 2009. For the ingroup, all ten described species as well as two newly discovered species of *Aphistogoniulus*, distributed above the whole island of Madagascar (Fig. 1) were added to the character matrix. All material was either the type series, or a specimen which was directly compared to the types. For all specimens studied see Table 1.

**Table 1:** Species included in the morphological character matrix, with their collection codes and depository.

Species	Deposited
*Epibolus pulchripes* (Gerstäcker, 1873)	ZMUC
*Madabolus maximus* Wesener & Enghoff, 2008	FMNH-INS 5466
*Corallobolus cruentus* Wesener, 2009	FMNH-INS 5397
*Sanguinobolus maculosus* Wesener, 2009	FMNH-INS 3918
*Colossobolus semicyclus* Wesener, 2009	FMNH-INS 5488
*Colossobolus oblongopedus* Wesener, 2009	FMNH-INS 55031
*Aphistogoniulus cowani* (Butler, 1882)	FMNH-INS 7791–7792
*Aphistogoniulus erythrocephalus* (Pocock, 1893)	FMNH-INS 3925
*Aphistogoniulus hova* (de Saussure & Zehntner, 1897)	CASENT 9032821
*Aphistogoniulus corallipes* (de Saussure & Zehntner, 1902)	FMNH-INS 56116
*Aphistogoniulus sanguineus* Wesener, 2009	FMNH-INS 7890
*Aphistogoniulus infernalis* Wesener, 2009	FMNH-INS 56007
*Aphistogoniulus diabolicus* Wesener, 2009	FMNH-INS 6169
*Aphistogoniulus aridus* Wesener, 2009	CAS BLF 5241
*Aphistogoniulus vampyrus* Wesener, 2009	FMNH-INS 5414
*Aphistogoniulus rubrodorsalis* **n. sp.**	FMNH-INS 55999
*Aphistogoniulus jeekeli* **n. sp.**	CASENT 9032822

CAS = California Academy of Sciences; FMNH = Field Museum of Natural History; ZMUC = Natural History Museum of Denmark.

#### Character selection

A character matrix was created using Mesquite 2.6 ([38], see Table 2). A total of 35 characters were selected, focusing on the male copulatory devices (gonopods, see also Supporting Information S5). Two character sets of *Aphistogoniulus*, the endotergum ( = underside of the body rings) and the female vulva (see Supporting Information S3) were illustrated for the first time (see Supporting Information S4). Due to a lack of sufficient material, endotergum characters could not be added to the matrix (see Supporting Information S6). All characters were scored as ‘unordered’ with equal weights. Drawings were produced using a standard camera lucida mounted on an Olympus SZH10 dissecting microscope. For scanning electron microscopy, samples were cleaned and dehydrated in an ethanol series and air-dried overnight before being sputter coated (Denton Vacuum Desk IV) for 180 seconds. SEM micrographs were taken using a Zeiss (Leo) EVO SEM, based at the FMNH.

**Table 2:** Morphological Character matrix.

	Characters			
Species	1–10	11–20	21–30	31–35
*Epibolus pulchripes*	1111102000	0000000000	---000-00-	00-00
*Madabolus maximus*	1111102000	0000000000	---000-00-	00-00
*Corallobolus cruentus*	0000011000	0000000000	---000-00-	00-00
*Sanguinobolus maculosus*	0000002110	0011000011	101000-00-	00-00
*Colossobolus semicyclus*	0000002111	1110000111	011000-00-	00-00
*Colossobolus oblongopedus*	0000002111	1110000111	011000-00-	00-00
*Aphistogoniulus cowani*	0000002000	0000110011	0001111000	00100
*A. erythrocephalus*	0000002000	0000110011	0000100000	01100
*A. hova*	00---02000	0000110011	0000101000	01100
*A. corallipes*	00---02000	0000111011	0000000000	00200
*A. sanguineus*	00---02000	0000110011	0001111000	00100
*A. infernalis*	00---02000	0000111011	0000000111	10100
*A. diabolicus*	00---02000	0000111011	0000000000	00000
*A. aridus*	00---02000	0000111011	0000000111	00001
*A. vampyrus*	00---02000	0000111011	0000000111	00001
*A. rubrodorsalis* **n. sp.**	00---02000	0000110011	0001111000	00100
*A. jeekeli* **n. sp.**	0000002000	0000111011	0000000011	00010

#### Phylogenetic analysis

A maximum parsimony Branch and Bound tree search was conducted in PAUP* 4.0b10 [39]. Accelerated transformation was used as character optimization criterion. Six of the 35 characters were parsimony uninformative. The search yielded a single shortest tree with a length of 35. Furthermore, a bootstrap analysis, incorporating 2000 pseudoreplicates, was undertaken in PAUP 4.0b10 under the tree-bisection-reconnection (TBR) branch-swapping algorithm.

### Molecular Phylogenetic Analysis

#### Taxon selection

Sequences of the Spirostreptida species *Doratogonus* sp., one of the few millipedes for which the 18S [AY288687.1], COI [AY288738.1] and 16S [AY288715.1] genes are available on Genbank, were downloaded and used as far-outgroup. Because of the unclear position of the genus *Aphistogoniulus* inside the family, *Madabolus* with its type species *M. maximus* of the tribe Pachybolini [36], and two species of the subfamily Spiromiminae [40], *Spiromimus simplex* Wesener & Enghoff, 2009 and *S. triaureus* Wesener & Enghoff, 2009 were both chosen as far-outgroups for the Spirobolida. Furthermore, the type species of the genus *Colossobolus*, *C. semicyclus* Wesener, 2009, was chosen as near-outgroup because of morphological similarity. All available species of *Aphistogoniulus*, including populations from different areas, were included. Eight of the twelve *Aphistogoniulus* species (25 different terminals) were successfully sequenced. The four missing species (*A. sakalava*, *A. rubrodorsalis* n. sp., *A. diabolicus* and *A. aridus*) are currently only known from their original type series [34].

#### Specimen sampling and DNA extraction

Freshly collected specimens were euthanized through freezing, which relaxes the specimen and allows for further study without breaking. Two legs were removed immediately and stored in 95% ethanol which was changed once after approximately four weeks. The specimen was then transferred to 75% ethanol. The other 22 specimens were previously stored in 75% ethanol and were collected between 1995 and 2006 (see Supporting Information S1 and Supporting Information S8). From these specimens, muscle from the male gonopods was removed and transferred to 95% ethanol. All tissue samples came from male specimens, with the exception of *M. maximus* B and *A. infernalis* E which came from mature females. Total genomic DNA was extracted using the DNAeasy® Blood & Tissue kit from Qiagen™, following the manufacturer's extraction protocol. The leftover DNA extraction product as well as the complete second elution will be stored at the Field Museum's cryogenic storage facility. To allow a later identification and verification of the species after a taxonomic update, voucher specimens were deposited (see Table 3).

**Table 3:** Newly sequenced species, Museum voucher and GenBank access codes.

Species	Voucher #	nc 18S	mt CoI	mt 16S
*Madabolus maximus* A	FMNH-INS X01	HQ891264	HQ891241	-
*Madabolus maximus* B	FMNH-INS X02	HQ891265	HQ891242	-
*Spiromimus simplex*	CASENT 9032813	HQ891266	HQ891243	HQ891215
*Spiromimus triaureus*	CASENT 9032804	HQ891267	HQ891244	HQ891216
*Colossobolus semicyclus* A	CASENT 9032800	-	HQ891239	-
*Colossobolus semicyclus* B	CASENT 9032801	HQ891263	HQ891240	-
*Aphistogoniulus vampyrus* A	FMNH-INS 5387	HQ891259	-	HQ891213
*Aphistogoniulus vampyrus* B	FMNH-INS 5366	HQ891260	HQ891237	-
*Aphistogoniulus vampyrus* C	FMNH-INS 5392	HQ891261	HQ891238	HQ891214
*Aphistogoniulus vampyrus* D	FMNH-INS 5414	HQ891262	-	-
*Aphistogoniulus infernalis* A	FMNH-INS	HQ891252	HQ89127	HQ891204
*Aphistogoniulus infernalis* B	FMNH-INS	HQ891253	HQ89128	HQ891205
*Aphistogoniulus infernalis* C	FMNH-INS-56488	HQ891254	HQ89129	HQ891206
*Aphistogoniulus infernalis* D	FMNH-INS-56488	HQ891255	-	HQ891207
*Aphistogoniulus infernalis* E	CASENT 9032823	HQ891256	HQ89130	HQ891208
*Aphistogoniulus corallipes* A	FMNH-INS-56116	HQ891245	HQ891217	HQ891196
*Aphistogoniulus corallipes* B	FMNH-INS-56116	HQ891246	HQ891218	HQ891197
*Aphistogoniulus jeekeli* n. sp. A	CASENT 9032790	HQ891257	HQ891231	HQ891209
*Aphistogoniulus jeekeli* n. sp. B	CASENT 9032822	HQ891258	HQ891232	HQ891210
*Aphistogoniulus erythrocephalus* A	CASENT 9032802	-	HQ891222	HQ891200
*Aphistogoniulus erythrocephalus* B	CASENT 9032810	-	HQ891223	-
*Aphistogoniulus hova* A	CASENT 9032803	HQ891249	HQ891224	HQ891201
*Aphistogoniulus hova* B	CASENT 9032821	HQ891250	HQ891225	HQ891202
*Aphistogoniulus hova* C	FMNH-INS-55886	HQ891251	HQ891226	HQ891203
*Aphistogoniulus cowani* B	FMNH-INS 7792	HQ891247	HQ891219	-
*Aphistogoniulus cowani* C	FMNH-INS 7791	-	HQ891220	HQ891198
*Aphistogoniulus cowani* D	FMNH-INS 7866	HQ891248	HQ891221	HQ891199
*Aphistogoniulus sanguineus* A	FMNH-INS 54	-	HQ891233	HQ891211
*Aphistogoniulus sanguineus* B	FMNH-INS 7904	-	HQ891234	-
*Aphistogoniulus sanguineus* C	FMNH-INS 44981	-	HQ891235	-
*Aphistogoniulus sanguineus* D	FMNH-INS 7890	-	HQ891236	HQ891212

For locality information, see Supporting Information S8.

#### DNA amplification and sequencing

Three genes, the mitochondrial partial 16S rRNA gene, the cytochrome oxidase *c* subunit I (COI) partial gene, and the complete nuclear 18S rRNA gene, were amplified using the polymerase chain reaction (PCR) [41]. All three genes and gene fragments were successfully used in previous millipede studies and provided good resolution up to family level [11]. Amplification reactions were carried out on a MJ Research© PTC 200 thermal cycler. Temperature profile and summaries of the amplification reactions can be found in previous studies [11]. Negative and positive controls were included in every PCR setup. The PCR product was purified with a commercial kit (QIAquick^©^ PCR Purification Kit, Qiagen GmbH). Cycle sequencing for sequencing was conducted with BigDye on a Bio-nad Dyad® DNA Engine, cleaned with ethanol on an Eppendorf^©^ centrifuge 5810R and sequenced on a Hitachi/AB 3730 automatic DNA sequencer, using the same primer sets as for PCR or, in the case of the 18S rDNA, specific primer pairs (see Supporting Information S9). Sequencing reads were assembled and proofread with the program Seqman™ II (DNASTAR, Inc.) while the identity of all new sequences was confirmed with BLAST searches [42]. All used primer sequences are listed in Supporting Information S9, while all new sequences were deposited in GenBank (see Table 3 for accession numbers).

#### Alignment

Datasets were aligned using MUSCLE Version 3.6 [43] with default settings, including gene sequences from a Spirostreptida as far-outgroup taxon (*Doratogonus* sp. [AY288687], [AY288738], [AY288715]). The final alignments consisted of 655 basepairs (bp) (COI), 513 bp (16S rRNA) and 1863 bp (18S rRNA). The alignment of the combined dataset can be found in the Supporting Information S7. The combined analysis consisted of 3031 bp. Uncorrected p-distances of all three genes are given in Supporting Information S10.

#### Maximum Parsimony phylogenetic analysis

The program PAUP*4.0b10 [39] was used for maximum parsimony (MP) analyses using the TBR branch swapping algorithm with an unenforced ‘MaxTrees’ option. The number of parsimony informative characters in the dataset was 645. Total number of shortest length trees with 2114 steps was 15, which all showed identical topologies on the species-level. A strict consensus tree was built out of all 15 shortest-length trees. To test if no tree island was overlooked during the heuristic search, a Winclada-Asado^©^ Ratchet analysis [44] was conducted under Asado version 1.7^©^. The Ratchet parameters were set to: number of iterations = 200 (default), number of trees to hold at each iteration = 5 (default = 1), number of characters to sample = 350 (default = 303). All other settings were within the default parameters of Asado 1.7.

To assess statistical support for hypothesized clades, 1,000 bootstrap pseudoreplicates were calculated with the program PAUP*4.0b10, under the TBR branch swapping algorithm, with one tree held at each step and without enforcing maximum trees.

#### Bayesian phylogenetic analysis

Bayesian analyses were conducted using MrBayes v3.1.2 [45]. Appropriate DNA-substitution models were determined separately for all analysed three genes under the Bayesian information (BIC) [46] implemented in jModeltest 0.1.1 ([47], [48]. In the Bayesian analysis, the combined data set was partitioned (see MrBayes manual for details) to allow unlinked models and model parameters for all three genes. Following the MrBayes manual, the most appropriate substitution model was specified without fixing the parameter values, allowing them to vary during the annealing process. This method leads to more conservative results but more realistic posterior probabilities. The Bayesian analysis was conducted by computing 3,000,000 Monte Carlo Markov chain (MCMC) generations in two parallel runs each with three cold chains and one hot chain. Trees were sampled every 100 generations. The number of burn-in generations was determined by manual inspection of the likelihood score over the 3,000,000 generations.

#### Maximum Likelihood phylogenetic analysis

Using RAxML 7.0.4 [49] a fast maximum likelihood (ML) method including bootstrap analyses with 10,000 pseudoreplicates was performed. The dataset was partitioned to allow different parameters for each of the three analysed genes under GTRMIX parameter settings (see RAxML 7.0.4 manual for details).

### Analysis of the Historical Biogeography of *Aphistogoniulus*

In order to explain the current biogeographic distribution of the analyzed eight species of *Aphistogoniulus* we used RASP (formerly S-DIVA) to reconstruct the habitat ranges of the ancestral taxa [50]. Habitat classifications were defined based on the collection site of the studied species (see Fig. 1), following the atlas of the vegetation of Madagascar. Habitat A refers to seasonal dry forests, habitat B refers to montane rain forests, habitat C refers to lowland rainforests and habitat D refers to mid elevation rain forests. It is important to note that the outgroup taxon *Colossobolus semicyclus* was chosen primarily for rooting the phylogeny, not for determining the ancestral habitat of the genus *Aphistogoniulus*. A Bayesian binary MCMC analysis was run using default settings: number of chains = 10, frequent of sample = 100, discard samples = 100, temperature = 0.1, maximum number of areas = 4, state frequencies = fixed (Jukes-Cantor), and an across-site rate variation = equal. However, we increased the number of cycles from 50,000 to 100,000. All results in detail are listed in Supporting Information S11.

## Results

### Morphologic Phylogenetic Analysis

**Figure 2:**
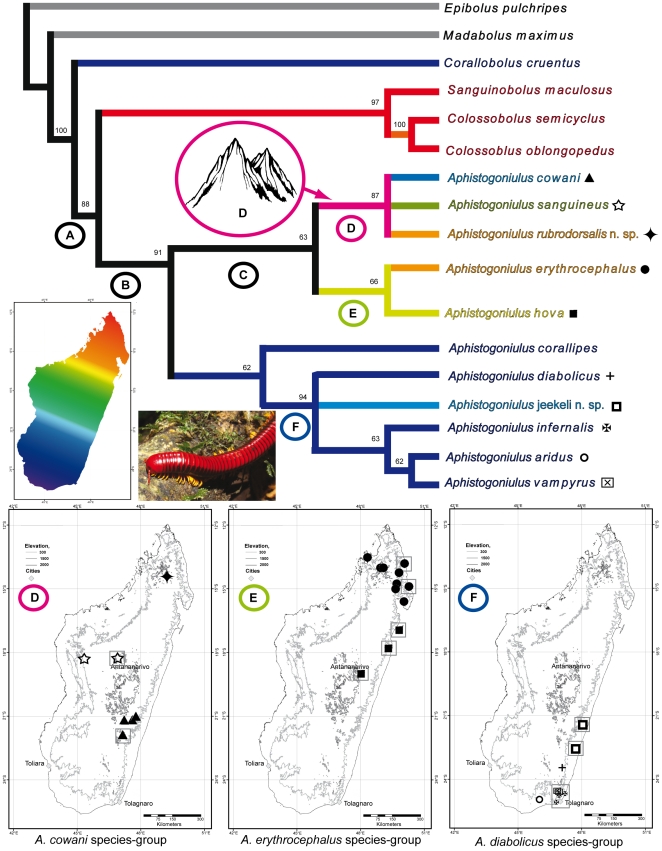
Phylogeny and Biogeography of *Aphistogoniulus*. Shortest tree found with Branch & Bound method. Numbers above branches refer to the bootstrap support of the node. Symbols behind species names match those of the distribution maps. Color of branches refers to the longitudinal distribution of the species, with red referring to northern, green to central and dark blue to southern distribution. Pink branches highlight the mountain clade.

The first split in the tree occurs between the members of the tribe Pachybolini (*Epibolus pulchripes, Madabolus maximus*) and the rest of the large-bodied Spirobolida from Madagascar (Fig. 2). Characters supporting this split are found in the anatomy of the head (Supporting Information S5, c1) and mouthparts (c2), as well as the special shape of the vulva (c3, c4, c5), and is also supported by a bootstrap value (100%) in the analysis (Fig. 2).

The next well-supported split divides the southern Malagasy genus *Corallobolus* from the three genera *Sanguinobolus*, *Colossobolus* and *Aphistogoniulus* (Fig. 2: clade A). All members of clade A share a strongly elongated coxite (Supporting Information S5, c19) and a disc-shaped telopodite of the posterior gonopods (S5, c20). *Sanguinobolus* & *Colossobolus* form the sister-group to *Aphistogoniulus* (Fig. 2). *Aphistogoniulus* is monophyletic in the tree (Fig. 2, clade B) and supported by apomorphies such as the elongation of the coxite process of the anterior gonopods (S5, c15) and the location of the telopodite retrorse process on the anterior gonopods (S5, c16).

Three clades are present within *Aphistogoniulus*, the *A. cowani, A. erythrocephalus* and *A. diabolicus* clades. The genus is divided by a basal split between the *A. cowani* and *A. erythrocephalus* sister clades (Fig. 2: clade C) and a clade comprising *A. corallipes* and the *A. diabolicus* clade (Fig. 2: clade F). Clade (C) is supported by the presence of a retrorse projection on the anterior side of the main branch of the posterior gonopods (S5, c25). The main apomorphy of the *A. cowani* clade is the lateral membranous fringe which is developed into a strong process on the main branch of the posterior gonopods (S5, c24). The *A. erythrocephalus* clade is corroborated by a sulcate fringe present on the apex of the posterior gonopod main branch (Supporting Information S2). Three characters support the *A diabolicus* clade: the special shape (S5, c29) and the sharp, pointed ending (c33) of the basal branch of the posterior gonopod, as well as the presence of a apico-mesal fringe on main branch of the posterior gonopod (c30). This clade also includes the only species of *Aphistogoniulus* adapted to arid environments, *A. aridus*, which is the sister taxon of *A. vampyrus* (Fig. 2). The current position of *A. corallipes* is not well-supported in the tree, being in a basal position towards the *A. diabolicus* clade.

### Molecular Phylogenetic Analyses

**Figure 3:**
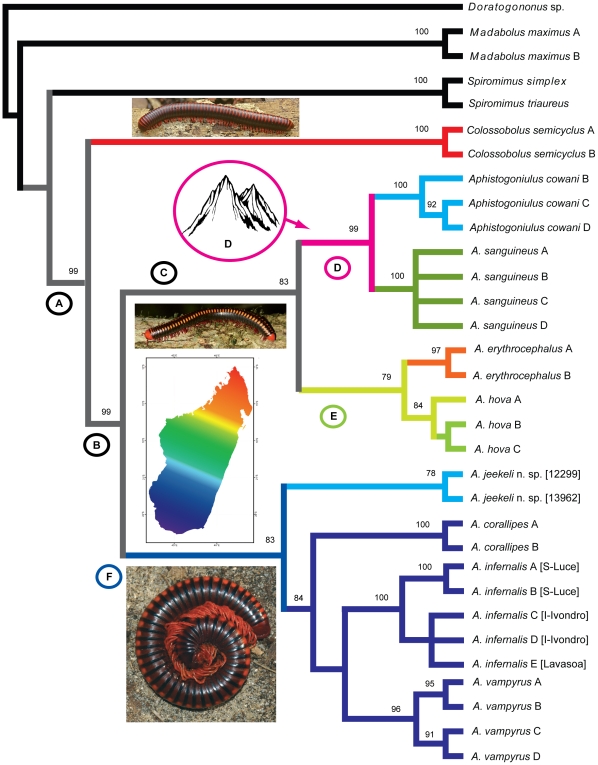
Maximum parsimony (MP) tree based on the combined (16S+CO1+18S) dataset (3033 bp, 455 parsimony-informative characters). Shortest tree found with Branch & Bound method. Numbers above nodes indicate statistical support based on MP bootstraps >50% of the combined analysis, below nodes of the 16S/18S/Co1 single gene analysis. Gaps (−) refer to bootstrap support <50%, ? to missing species (and therefore nodes) in the single gene analysis. Color photographs (above to below) show *Colossobolus* sp. (Arne Hartig), *Aphistogoniulus cowani* (Arne Hartig), *A. infernalis* (K. Schütte). Color of branches refers to the longitudinal distribution of the species, with red referring to northern, green to central and dark blue to southern distribution.

The molecular phylogenetic tree obtained by the parsimony analysis (Fig. 3) closely resembles the morphological one (Fig. 2). *Aphistogoniulus* and *Sanguinobolus* form a well-supported group (Fig. 3, clade A), distinct from the other Malagasy genera *Spiromimus* (Spiromiminae) and *Madabolus* (Pachybolini). The monophyly of *Aphistogoniulus* (Fig. 3, clade B) is also well supported with a bootstrap value of 99%. Inside *Aphistogoniulus*, we observe a basal split between the species of the *A. cowani*- and *A. erythrocephalus* group (clade C) and the *A. diabolicus* clade (Fig. 3, clade F). The *A. cowani* clade (Fig. 3, clade D) with *A. cowani* and *A. sanguineus* is strongly supported (99%) and clearly distinct from the clade incorporating *A. erythrocephalus* and *A. hova* (Fig. 3, clade D). *A. sanguineus* and *A. cowani* differ moderately in their mitochondrial DNA (COI: 14–16%, 16S: 4.4–5.7%, see Supporting Information S10). The *A. diabolicus* clade (Fig. 3, clade F) is statistically well-supported (83%) and also incorporates *A. corallipes*. All species of the clade (*A. jeekeli*, *A. corallipes*, *A. infernalis* and *A. vampyrus*) come out as monophyletic with strong statistical support. The northernmost species of the group, *A jeekeli* **n. sp.**, is the sister-taxon to all others, followed by *A. corallipes*, which is the sister taxon to the *A. vampyrus*/*A. infernalis* species-pair. The genetic difference between *A vampyrus* and *A. infernalis* is moderate (COI: 15–17%, 16S: 5.7–6.1%), despite their quite distinct copulation legs, especially the posterior gonopods [34]. In *A. infernalis* the specimens from the littoral rainforest of Sainte Luce are the sister group (differences COI: 6.6%, 16S: 2.0%) to populations from the isolated rainforest at Grand Lavasoa and from the Andohahela Mountain chain (Fig. 3).

For the Bayesian analysis, jModeltest indicated the following models as the appropriate nucleotide substitution models for the three analysed genes, including the following parameters: 18S rDNA – Selected model: TPM3+G; 16S rDNA – Selected model: TrN+G; COI – Selected model: SYM+I+G. For reasons described above, only the model, not the parameters, has been fixed in the Bayesian analysis. Manual inspection of the likelihood scores of the 2×30,001 trees samples in two parallel runs showed a good convergence after 650 trees, a value that has therefore been chosen as the burn-in. Posterior probabilities have been computed from a majority rule consensus tree of the remaining 2×29,351 trees.

**Figure 4:**
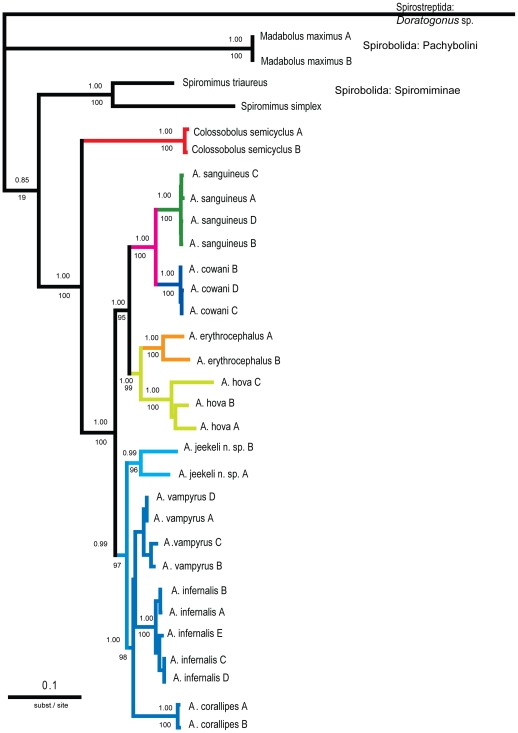
Maximum likelihood (ML) tree based on the combined (16S+CO1+18S) dataset (3033 bp).

The Bayesian tree (not shown, but values in Fig. 4), as well as the maximum likelihood tree (Fig. 4) show a topology identical to the maximum parsimony trees (Fig. 3). The branch length of the likelihood tree indicates larger intraspecific divergence in *A. erythrocephalus*, *A. hova* and *A. jeekeli* n. sp. than in all other analysed species (Fig. 4).

### Biogeographical Patterns of *Aphistogoniulus*

**Figure 5:**
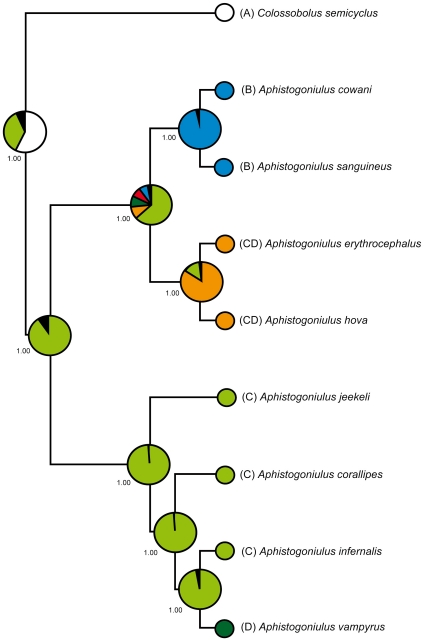
Graphical results of the ancestral habitats for the given molecular phylogeny of *Aphistogoniulus* using RASP. Pie charts at internal nodes show calculated probabilities of alternative ancestral habitats (detailed information are given in Supplementary Information S8; see also material and methods). Habitat color code: seasonal dry forest (A) = white, montane rainforest (B) = dark blue, lowland rainforest (C) = light green, mid elevation rain forests (D) = dark green, mixture of B and C = red, and a mixture of C and D = orange. Black sections indicate habitats with probabilities <5%. Numbers at nodes indicate estimated *p* values.

Members of *Aphistogoniulus* are generally distributed in the rainforests and montane rainforests of Madagascar, spanning from the northern massif of Marojejy towards the south at Andohahela and Ambatotsirongorongo. *Aphistogoniulus* species are often tree-climbers [34] and show a widespread distribution (Fig. 1). The elevation-range, even on species-level, varies from close to sea level to mountaintops [34]. Only one *Aphistogoniulus* species, *A. aridus*, is currently known from a dry habitat, the southern spiny forest of Mahavelo. In the far north of Madagascar, *Aphistogoniulus* seems to be replaced by its sister-genera *Colossobolus* and *Sanguinobolus* (Fig. 2). In this context, RASP indicates that the most likely ancestral habitat for the genus *Aphistogoniulus* was lowland rainforest (90.2%, Fig. 5) which is still inhabited by five species (*Aphistogoniulus jeekeli*, *A. corallipes, A. infernalis, A. hova*, and *A. erythrocephalus*). Interestingly, two species that are restricted to montane rainforests (*Aphistogoniulus cowani* and *A. sanguineus*) represent the sistergroup of two other species which are found in both lowland and mid-elevation rainforests (*Aphistogoniulus erythrocephalus* and *A. hova*). These results give evidence for a trend of colonization of higher rainforest levels from lowland rainforests within this clade.

Two of the three *Aphistogoniulus* clades match a general distribution pattern; the *A. erythrocephalus* clade is distributed in the northern and median longitudes (Fig. 2, middle) in both lowland and mid-elevation rainforests. All representatives of the *A. diabolicus* clade can be found south of the 21° longitude (Fig. 2, right), and include the only species of the genus recorded from an arid ecosystem. Inside the *A. diabolicus* clade, the northern-most species (*A. jeekeli*) is the most basal species (Fig. 3).

The distribution of the *A. cowani* clade is not consistent with this coherent biogeographical pattern. Its three members show a disjunctive distribution in mountains of the south (*A. cowani*), the middle (*A. sanguineus*) and the north (*A. rubrodorsalis*) of Madagascar (Fig. 2). The *A. cowani* clade also shows an unusual pattern regarding the altitude: its members seem to be restricted to montane forests, only living in areas of more than 900 m elevation.

## Discussion

### Speciation Model for Aphistogoniulus

The majority of *Aphistogoniulus* species, including all members of the *A. erythrocephalus* and the *A. diabolicus* clades, show a stochastical distribution pattern which can best be explained by a ‘simple’ model reflecting speciation-by-distance, or simply coherent parapatric speciation. Our analyses give evidence that the most likely habitat for *Aphistogoniulus* was lowland rainforest (90.2%, Fig. 5), supporting these ideas. Major rivers do not appear to delineate species distribution borders (Fig. 1). At two nodes (Fig. 2), the phylogeny of *Aphistogoniulus* reflects two speciation models that are currently the subject of intense discussion [12].

(1) The *A. cowani* clade, clade D in our cladogram (Fig. 2), includes species restricted to areas with an elevation higher than 900 m. Here, the most congruent evolutionary model explaining the existence of such a clade is mountain refugia [13]. The ancestor of the *A. cowani* clade was probably a widespread species inhabiting lowland rainforests (63.6% probablity, see Fig. 5). The recent species are distributed in the Andringitra and Ranomafana massif (*A. cowani*), the Ambohijanahary and Ambohitantely Mountains (*A. sanguineus*), and Marojejy (*A. rubrodorsalis*). Distribution and speciation patterns reflecting the *A. cowani* clade were previously discovered in dwarf chameleons of the genus *Brookesia* [14], which occur in a similar ecological niche (leaf litter and low vegetation).

(2) The *A. vampyrus* - *A. aridus* species pair is an example of speciation possibly caused by an ecotone shift. The species *A. vampyrus* is like all other *Aphistogoniulus* species distributed in the rainforest, in this case the rainforests of Andohahela in south-eastern Madagascar. Its sister-taxon *A. aridus* is currently only known from the Mahavelo forest, a spiny forest located approximately 60 km from Andohahela [51]. Here we have an ancestor (potentially *A. vampyrus*) originating in the south-eastern rainforest colonizing and adapting to the southern spiny forest (*A. aridus*). This speciation model was coined “ecotone shift” in other Malagasy animals, including amphibians and lemurs [12], while in Malagasy giant pill-millipedes such an ecotone shift seems to have occurred in the opposite direction from *Aphistogoniulus*: within the genera *Zoosphaerium* [52] and *Sphaeromimus* [53], the most basal species are distributed in the dry spiny forest ecosystem [10], [11].

### Molecular phylogeny of *Aphistogoniulus* vis-à-vis other molecular millipede studies

This is the first molecular millipede study on species level in the Southern hemisphere, and therefore the first on Madagascar. Millipede molecular studies on species level are still less than 10 and greatly focus on North America [35], with a single one also conducted in Japan [54]. In North America, Ice Age refugia were discovered to play a role in the current distribution of haplotypes of the spirobolid millipede genus *Narceus*. Unfortunately, the historical climate on Madagascar is still too little known for any comparison. There does not seem to be any indication of glaciation during the last ice ages on Madagascar. While the molecular Spirobolida dataset cannot be dated due to lack of fossils, the relatively large genetic distances between the different *Aphistogoniulus* species (14–30% of COI, 5–13% in the 16S, Supporting Information S5) are an indication that the speciation events in *Aphistogoniulus* predate the most recent ice ages.

### Divergent Gonopod Evolution in the *A. diabolicus* Clade

**Figure 6:**
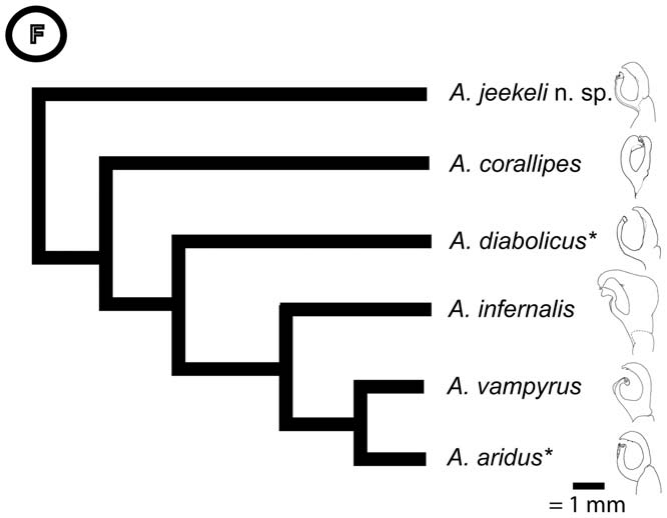
Phylogeny of the *Aphistogoniulus diabolicus* clade with schematic drawing of the disc of the posterior gonopod (modified leg podomere used in sperm transfer), anterior view. For species marked with an asterisk, no sequence data was available.

The disc of the posterior gonopods is shaped like an almost closed ‘C’ in most members of the *A. diabolicus* clade, including *A. jeekeli* n. sp., *A. diabolicus*, *A. vampyrus* and *A. aridus* (Fig. 6), and displays only minor differences between species. However, the two other species of the *A. diabolicus* clade, *A. corallipes* and *A. infernalis*, show strongly modified posterior gonopods, where in the former the main branch and in the latter the basal branch are greatly enlarged (Fig. 6). This finding needs further investigation (especially if the corresponding female organs are also enlarged), and is another hint that divergent evolution in sexual morphology of related organisms with limited dispersal ability plays a role in speciation [54].

### Analysis Problems

The current morphological dataset of *Aphistogoniulus* is comprised of only a very limited number of characters (Supporting Information S5). Although all higher clades are supported by good apomorphies, more characters would give a higher resolution to the analysis on the species level. While more morphological characters (such as the endotergum or the mandible) could be explored with a scanning electron microscopy study, it is questionable if (a) enough specimens of all rarely collected *Aphistogoniulus* species can be made available, and (b) if such characters really vary enough and are independent enough to provide a more robust phylogeny of *Aphistogoniulus*. Our reconstruction of the historical biogeography of *Aphistogoniulus* was also restricted to the molecular data set.

The three *Aphistogoniulus* species missing from the molecular analysis are those species only known from their type series. The absence of sequence data for *A. rubrodorsalis* n. sp., *A. diabolicus* and *A. aridus* is regrettable, but all three species are morphologically close enough to their respective sister taxa. Therefore, their exclusion from the molecular analysis does not likely affect the obtained results.

### Description of two new species of *Aphistogoniulus*

Genus *Aphistogoniulus* Silvestri, 1897

*Mystalides* Attems, 1910

See Wesener *et al.* [34] for a revision and complete systematics of the genus and its species.

*Aphistogoniulus rubrodorsalis* n. sp. Decker & Wesener

Red-Back Fire-Millipede

urn:lsid:zoobank.org:pub:BE53B87F-9FDA-4B75-B461-A7110D373A99

#### Derivatio nominis

Rubrodorsalis, adjective, consisting of the assembled Latin words ruber = red and dorsalis = back, referring to the blood-red dorsal side of the species.

#### Material examined

1 M

#### Holotype

1 M, **FMNH-INS 55999**, Madagascar, Province Antsiranana, PN de Marojejy, along tributary of Manantenina River, 10.5 km NW Manantenina, montane rainforest, 1625 m, 14°26′40″S, 49°44′05″E, leg. S. Goodman et al., general collecting, 3–9.ix.1997.

#### Diagnosis

**Figure 7:**
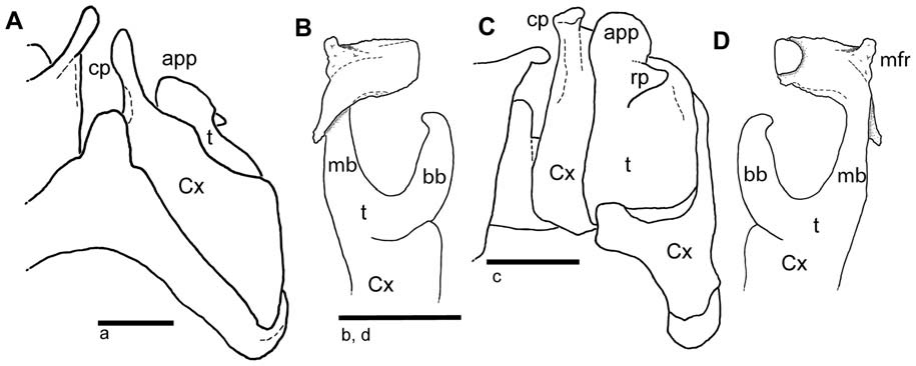
*Aphistogoniulus rubrodorsalis* n. sp., holotype (FMNH-INS 7914) A: anterior gonopods, anterior view; B: right posterior gonopod, anterior view; C: anterior gonopods, posterior view; D: right posterior gonopod, posterior view. **Abbreviations**: app = appendage; bb = basal branch; cp = coxite process; Cx = coxite; mb = main branch; mfr = membranous fringe; rp = retrorse process of appendage; St = sternite; t = telopodite. Scale bars = 1 mm.

Main branch of telopodite of posterior gonopods with lateral membranous fringe strongly developed into erect process (Fig. 7B), a character only shared with *A. sanguineus* and *A. cowani*, identifying this species as a member of the *A. cowani* clade (Fig. 2, clade D). Distinguishable from the latter two species by a massive and apically curved basal branch of telopodite of posterior gonopods (Fig. 7D), which is slender in both *A. cowani* and *A. sanguineus*, and only in *A. sanguineus* more conspicuously curved. Retrorse process of anterior gonopod in A. rubrodorsalis **n. sp.** is more strongly developed than in *A. sanguineus* and *A. cowani* (Fig. 7C). Apical lobe of main branch of posterior gonopod folded backwards at a straight angle (Fig. 7D), while it is more diagonally folded in *A. cowani* and *A. sanguineus*.

### Description

#### Measurements

49 body rings, circa 91 mm long, 8 mm wide.

#### Color (in preserved specimen)

Head, antenna, legs and telson red. Anterior half of collum red, posterior half black. Body rings ventrally black, dorsally metazonites black and mesozonites red. Ozopores located in black area.

Antenna short, extending back to ring 4.

Anterior gonopod sternite with shoulders and well-rounded tip (Fig. 7A). Appendage of telopodite swollen, retrorse process strongly developed, projecting medially instead of apically (Fig. 7C). Process of coxite strongly elongated, extending far beyond telopodite (Fig. 6A).

Posterior gonopod telopodite branches forming a ‘J’ (Fig. 7D). Basal branch of telopodite massive, apically curved, membranous folds absent (Fig. 7b). Tip of main branch of telopodite apically and mesally with characteristic membranous fringes (Figs. 7B, D). Apical membranous fringe of main branch folded at straight angle (Fig. 7D). Width of main branch towards apex slightly increasing.

#### Conservation and distribution

A. rubrodorsalis is currently only known from the type locality, the Marojejy Mountain (Fig. 1).

*Aphistogoniulus jeekeli* n. sp. Decker & Wesener

Jeekel's Fire-Millipede

urn:lsid:zoobank.org:pub:BE53B87F-9FDA-4B75-B461-A7110D373A99

#### Derivatio nominis

Named to honor the late Myriapodologist Casimir Albrecht Jeekel.

#### Material examined

4 M, 3 F

#### Holotype

1 M, **CAS BLF 12299 (CASENT 9032790)**, Madagascar, Province Fianarantsoa, 7,6 km Kianjavato, Forêt Classée Vatovavy, rainforest, 175 m, 21°24′00″S, 47°56′24″E, leg. Brian L. Fisher *et al.*, general collecting, 6.–8.vi.2005.

#### Paratypes

1 F, **(CASENT 9032790)**, same data as holotype.

#### Other material examined

3 M, 2 F, **CAS BLF 13962 (CASENT 9032822)**, Province Fianarantsoa, 24.5 km SW of Farafangana, Réserve Speciale Manombo, rainforest, 30 m, 23°00′57″S, 47°43′08″E, Brian L. Fisher *et al.*, general collecting, 20.iv.2006.

#### Diagnosis

**Figure 8:**
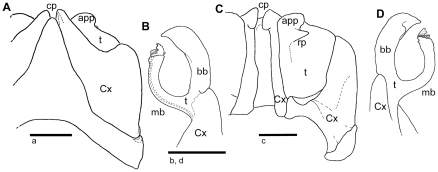
*Aphistogoniulus jeekeli* n. sp., holotype (CASENT 9032790) A: anterior gonopods, oral view; B: left posterior gonopod, oral view; C: anterior gonopods, anal view; D: left posterior gonopod, anal view. **Abbreviations**: app = appendage; bb = basal branch; cp = coxite process; Cx = coxite; mb = main branch; mfr = membranous fringe; rp = retrorse process of appendage; St = sternite; t = telopodite; ml = mesal lobe. Scale bars = 1 mm.

Special shape and sharp-edged ending of basal branch of posterior gonopod (Fig. 8B), and presence of an apico-mesal fringe on its main branch (Fig. 8D) identify *A. jeekeli* as a member of the *A. diabolicus* clade (Fig. 2, clade F). Membranous folds also present mesally on main branch of posterior gonopod (Fig. 8B), a character shared only by *A. aridus* and *A. vampyrus*. *A. jeekeli* can be distinguished from *A. aridus* and *A. vampyrus* by the presence of a unique rounded lobe (Fig. 7D) on the basal branch of posterior gonopod: only sclerotized spines are present in *A. aridus* and *A. vampyrus*. Main branch of posterior gonopod apically undulated (Fig. 8B). Coxite process of anterior gonopod slightly longer than telopodite (Fig. 8C). Collum completely red.

### Description

#### Measurements

Male: 53–58 body rings, circa 130 mm long, 9 mm wide. Female: 50–57 body rings, circa 135 mm long, 11 mm wide.

Color of head, antenna, collum, legs and telson red. Mesozonites of body rings dorsally red, ventrally darker. Anterior half of metazonite black, posterior half red. Red color extends ventrally along whole metazona. Ozopores marked in last third of body by black spots, which become larger towards telson.

Antenna short, extending back to ring 4.

Anterior gonopod sternite triangular, no shoulders, rounded tip (Fig. 7A). Coxite process elongated, as long as or slightly longer than telopodite (Figs. 7A, C). Telopodite appendage only weakly swollen, sharp-edged retrorse process starting to project medially.

Posterior gonopod telopodite branches forming an ‘O’ (Fig. 8B). Tips of main and basal branch close to but not touching one another. Main branch at mesal margin and apically with membranous folds, tip bi-lobed (Fig. 8D). Main branch slenderer and shorter than basal branch. Basal branch along its length of equal width, but tapering at apex (Figs. 8B, D); mesally with unique rectangular lobe (Fig. 8D).

#### Intraspecific variation

There is a difference in the number of body rings between the two observed populations. The specimens of Vatovavy possess 57 (F) and 58 (M) body rings, whereas the male specimens from Manombo only have 53 or 54 body rings and the two females 50 body rings, respectively. The population from Manombo is only tentatively placed in A. jeekeli because of strong morphological similarities, their gonopods and coloration are very similar. The large genetic distances between both populations (COI: 15.2%, 16S: 8.2%, 18S: 0.8%, see Supporting Information S10) are, however, only based on a single specimen from each population. Larger samples should be analyzed to discover if the population from Manombo might represent a distinct species.

#### Conservation and distribution

*A. jeekeli* is distributed in lowland rainforests of southeastern Madagascar. Both known localities, Vatovavy and Manombo, are isolated rainforest vestiges. Although the locations are 180 kilometers apart, all natural forests in between the locations are now degraded to pseudosteppe [54]. None of the current areas of distribution are effectively protected.

### Outlook

The genetic differences between different populations of the widespread northern species *A. erythrocephalus* and *A. hova* are conspicuous enough to look into biogeographic patterns and possibly even cryptic speciation in those taxa when more properly preserved specimens become available. Further studies are necessary to highlight intraspecific differences or even cryptic speciation in two species of the *A. cowani* clade. Populations of *A. cowani* show color differences (amount of red on the collum, [34]) between populations in Ranomafana and Andringitra-Ivohibe (100 km distance). Also, the long distance (180 km) between both known populations of *A. sanguineus* (Ambohijanahary and Ambohitantely) needs to be further investigated for eventual gene flow and/or undiscovered populations in intermediate areas. Unfortunately, no appropriately conserved material from any of these populations is currently available for a genetic study.

The discovery of two new *Aphistogoniulus* species in the last year highlights how little we know about the millipede fauna of Madagascar. It is already difficult to say if the current distribution of the highland species *A. sanguineus* is so disjunctive because of recent habitat fragmentation or ecological specialization. Highland vegetation between the closely related *A. sanguineus* and the eastern species *A. cowani* and *A. rubrodorsalis* is now completely degraded [55], [56], creating a void of information that hampers biogeographic studies [17].

Phylogenetic data remains lacking for most other millipede genera from Madagascar. In the order Spirobolida, only the genus *Spiromimus* of the subfamily Spiromiminae shares the wide distribution pattern of *Aphistogoniulus* [40], all other genera have a much more restricted range. A phylogenetic analysis of the Spiromiminae based on a morphological dataset showed several ecotone shifts occurring between rainforest and dry forest species of this subfamily [40]. The more mobile habit of the fire-millipede genus *Aphistogoniulus*, with its numerous widespread species, is in itself unusual for Malagasy millipedes. The wide range of the genus *Aphistogoniulus* and most of its species (Fig. 1), especially in comparison to other Spirobolida genera on Madagascar, could be related to its adaptation to a specific ecological niche, the surface of the leaf litter and lower branches of trees [34].

It might be interesting to compare the speciation pattern of the mobile *Aphistogoniulus* species with those of other Malagasy millipedes which occupy a different, more cryptic ecological niche, such as the wood-living genus *Hylekobolus* Wesener, 2009 (Spirobolida: Spirobolellidae [37]), or the giant pill-millipede genus *Sphaeromimus* (Sphaerotheriida: Arthrosphaeridae) that lives in the leaf litter.

## Supporting Information

Supporting Information S1 New locality data and notes for described *Aphistogoniulus* species.
(DOC)

Supporting Information S2 *Aphistogoniulus hova*, from Andasibe (FMNH-INS 55886).
(TIF)

Supporting Information S3 New character sets for *Aphistogoniulus* classification and taxonomy.
(DOC)

Supporting Information S4 SEM images of *Aphistogoniulus*.
(TIF)

Supporting Information S5 Character discussion.
(DOC)

Supporting Information S6 Character matrix as nexus file.
(DOC)

Supporting Information S7 Alignment of the combined (16S+CO1+18S) dataset as nexus file.
(DOC)

Supporting Information S8 Specimens, voucher code and locality information of specimens used for the molecular analysis. All 31 specimens, belonging to four genera and 12 species, were newly sequenced. Abbreviations: FMNH-INS = Field Museum insect collection number; CASENT = California Academy of Sciences Entomology specimen number.
(DOC)

Supporting Information S9 PCR primers & Sequencing primers.
(DOC)

Supporting Information S10 Uncorrected Pairwise Distances of the COI, 16S, and 18S dataset.
(XLS)

Supporting Information S11 Result file of the Bayesian analysis using RASP.
(DOC)
