# Supplementary material for: Mountain Refugia Play a Role in Soil Arthropod Speciation on Madagascar: A Case Study of the Endemic Giant Fire-Millipede Genus Aphistogoniulus
Source: PLoS One. 2011 Dec 6;6(12):e28035. doi: 10.1371/journal.pone.0028035 (PMC3232213; doi:10.1371/journal.pone.0028035)
Supplement: Supporting Information S1 — New locality data and notes for described Aphistogoniulus species. (DOC) [file pone.0028035.s001.doc]

**Supporting Information S1: New locality data and notes for described *Aphistogoniulus* species**

***Aphistogoniulus hova*** (de Saussure & Zehntner, 1897)

Common Madagascar Fire-Millipede

**Paratype**: 1 M, **MNHG**, Madagascar, det. H. de Saussure. Erroneously labeled as 'holotype' by a later worker.

**New locality data**: 1 M, **CAS** **BLF 13243** (CASENT 9032803), Madagascar, Province Toamasina, Reserve Betampona, Camp Vohitsivalana, 37.1 km 338° Toamasina, rainforest, 520 m, 17°53'12" S, 049°12'09" E, leg. Brian L. Fisher et al., 1–3.xii.2005; 3 M, **CAS BLF 12990** (CASENT 9032821), Madagascar, Province Toamasina, Ile Sainte Marie, Forêt Kalalao, 9.9 km 34° NW Ambodifotatra, rainforest, 100 m, 16°55'21" S, 049°53'14" E, leg. Brian L. Fisher et al., 24–27.xi.2005; 1 M, **MNHG**, Madagascar, imported to Germany by OdenwaldExoten, most likely from Province Toamasina, Andasibe (Périnet), close to Périnet-Analamazaotra-Reserve, 2009; 2 F, **MNHG**, same data as previous; 2 M, 1 F, **FMNH-INS 55886**, same data as previous; 1 M, **ZMUC**, same data as previous; 2 M, 1 F, Madagascar, imported to Germany by b.t.b.e Insektenzucht, most likely from Province Toamasina, Andasibe (Périnet), close to Périnet-Analamazaotra-Reserve, 2007.

**Distribution**: eastern lowland rainforests (Fig. 1).

**Description of population from Andasibe**:

*Measurements*: males with 53–57 body rings, holotype circa 105 mm long, 8.5 mm wide. Female with 53–56 body rings, circa 125–130 mm long, 10 mm wide

*Color* in living specimens pitch black/blood red. Head, antenna, collum, legs and telson red. Collum with more or less developed dark spot which can be almost absent. Legs in living specimens red, with yellow tibia and tarsi. Legs fade in alcohol to light brown. Mesozonites of body rings dorsally red, red coloration not reaching ventrally to ozopores. Anterior region of metazonites dorsally is black of variable extend, from a thin stripe to a nearly complete cover of metazonite. Posterior half of metazonite always with a narrow reddish margin dorsally and ventrally.

*Antenna* short, extending back to segment 4.

*Anterior gonopod* sternite broadly triangular with rounded tip (Fig. S2). Coxite process elongated, as long as, or slightly longer than telopodite (Figs S2A, C). Telopodite appendage swollen, tip extending into strongly developed, well-rounded retrorse process (Fig. S2C).

*Posterior gonopod* telopodite branches forming a ‘C’ (Fig. S2B). Basal branch short and as wide as main branch (Fig. S2D). Width of main branch apically increasing, tip flipped backwards anteriorly (Fig. S2B). Tip of main branch with membranous fringe, whose folds overlap several times mesally along lateral edge of curved branch (Fig. S2B).

**Intraspecific variation**: the number of body rings varied among specimens from the same sample and even more so between the group of specimens imported in 2007 and those imported in 2009.

**Conservation and distribution**: *A. hova* is since several years available in the U.S: and European pet trade, and commonly known and sold as the 'Madagascar Fire-millipede'.

***Aphistogoniulus erythrocephalus*** (Butler, 1882)

**New locality data**: 1 M, 1 F, **CAS** **BLF 11097** (CASENT 9032802), Madagascar, Province Antsiranana, Ambondrobe, 41.1 km 175° Vohemar, littoral rainforest, 10 m, 13°42'55" S, 050°06'06" E, leg. B. L. Fisher, hand collecting, 29.xi.2004; 1 M, **CAS BLF 11000** (CASENT 9032810), Madagascar, Province Antsiranana, Forêt Ambanitaza, 26.1 km, 347° Antalaha, rainforest, 240 m, 14°40'46" S, 050°11'01" E, leg. B. L. Fisher, hand collecting, 26.xi.2004.

**Distribution**: rainforests in northern Madagascar (Fig. 1).

***Aphistogoniulus infernalis*** Wesener, 2009

**New locality data**: 1 F, **CAS BLF 15414 (CASENT 9032823)**, Madagascar, Province Toliara, Ambatotsirongorongo, Grand Lavasoa, 25.9 km W Tolagnaro, 450 m, rainforest, 25˚05'16"S, 046˚44'56"E, leg. B. L. Fisher et al., sifted litter, 30.XI.2006.

**Comments:** No *Aphistogoniulus* specimen could be found in 2007 at the Ambatotsirongorongo sites Petit Lavasoa and Grand Lavasoa despite intense searching efforts by the first author. The collection attempt was made in June 2007, the same time period this species was active at Sainte Luce and Andohahela. The identity of the female specimen from Ambatotsirongorongo was confirmed by the molecular analysis (Fig. 3), highlighting the importance of molecular methods to identify difficult to determine specimens, such as female Diplopoda.

***Aphistogoniulus corallipes*** (deSaussure & Zehntner, 1902)

**Correction of error**: In a recent revision of this species [34], the orientation of the posterior gonopod was illustrated incorrectly, the mesal and lateral sides were accidentally confused. The branch labeled as 'main branch' is correctly identified as the lateral 'basal branch' and vice versa. A closer examination also revealed that the sperm groove in *A. corallipes* runs through the mesal main branch like in all other *Aphistogoniulus* species. A corrected figure of the posterior gonopod is presented here (Fig. 6).
