# Supplementary figures and images for: Mountain Refugia Play a Role in Soil Arthropod Speciation on Madagascar: A Case Study of the Endemic Giant Fire-Millipede Genus Aphistogoniulus
Source: PLoS One. 2011 Dec 6;6(12):e28035. doi: 10.1371/journal.pone.0028035 (PMC3232213; doi:10.1371/journal.pone.0028035)

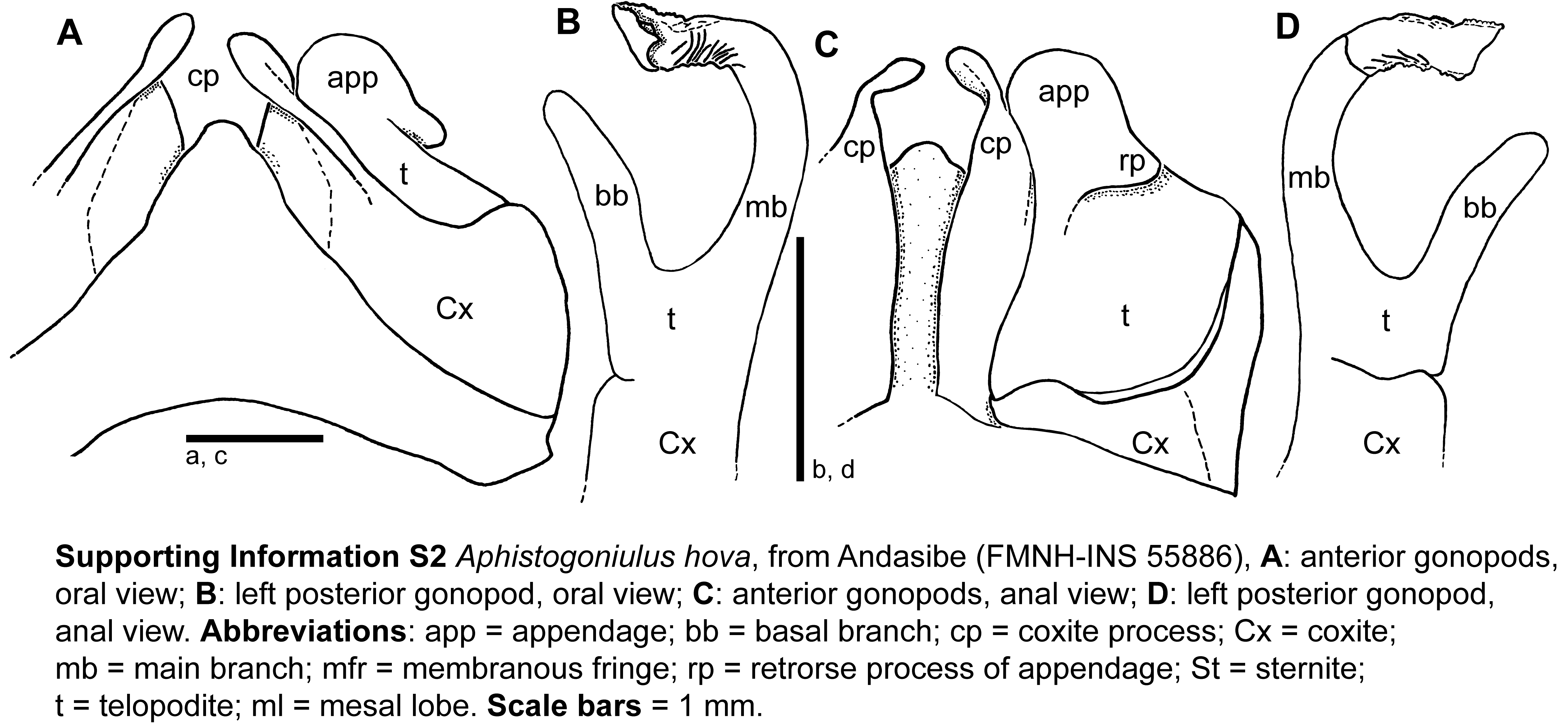

Supplement: Supporting Information S2 — Aphistogoniulus hova, from Andasibe (FMNH-INS 55886). (TIF) [file pone.0028035.s002.tif]

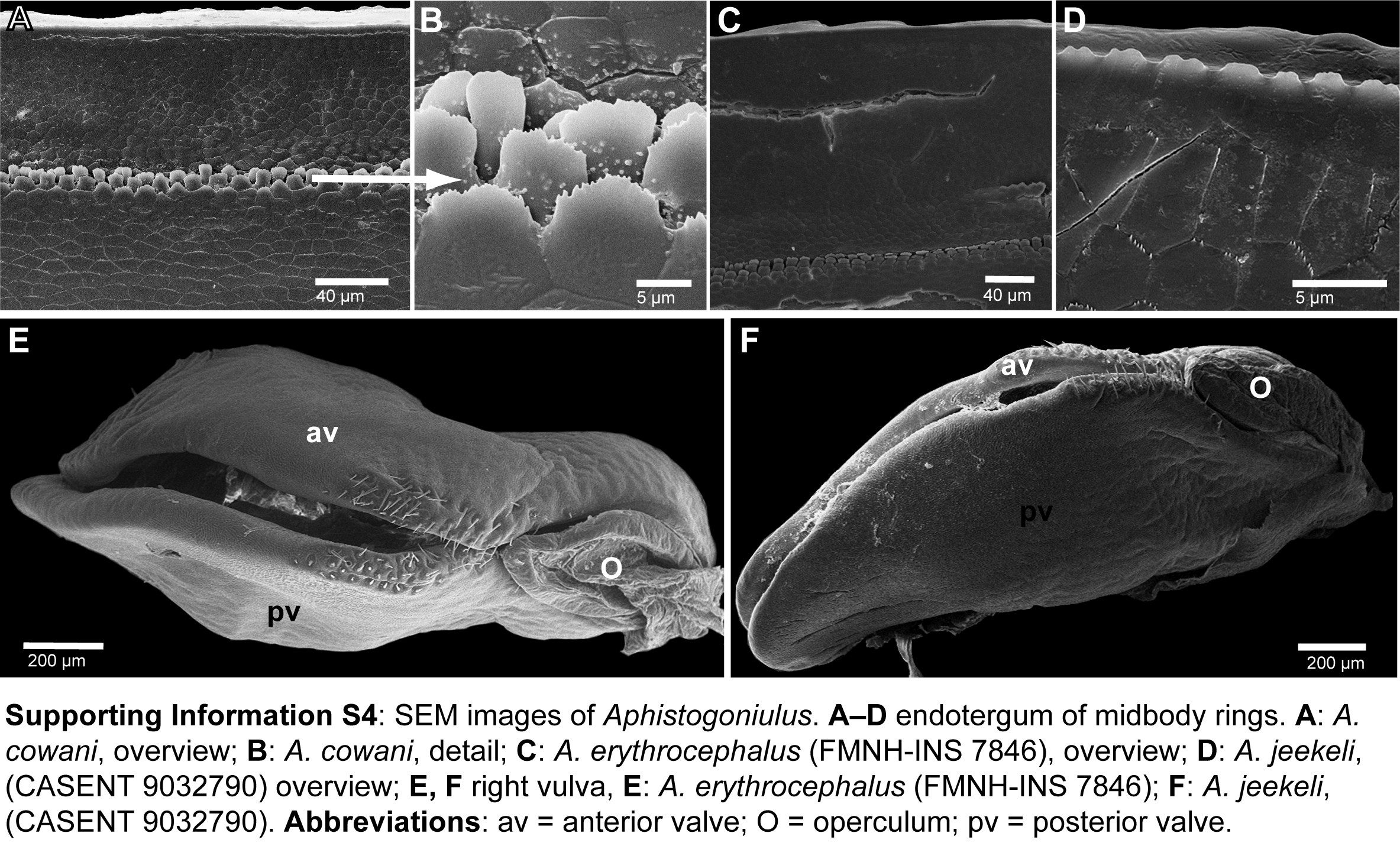

Supplement: Supporting Information S4 — SEM images of Aphistogoniulus. (TIF) [file pone.0028035.s004.tif]
