# Supplementary material for: Mountain Refugia Play a Role in Soil Arthropod Speciation on Madagascar: A Case Study of the Endemic Giant Fire-Millipede Genus Aphistogoniulus
Source: PLoS One. 2011 Dec 6;6(12):e28035. doi: 10.1371/journal.pone.0028035 (PMC3232213; doi:10.1371/journal.pone.0028035)
Supplement: Supporting Information S3 — New character sets for Aphistogoniulus classification and taxonomy. (DOC) [file pone.0028035.s003.doc]

**Supporting Information S3: New character sets for Aphistogoniulus classification and taxonomy:**

Two character sets were investigated for the first time in *Aphistogoniulus*, the female vulva and the endotergum (posterior margin of the underside of body rings).

*The female vulva:* The vulva of *Aphistogoniulus* (Supporting Information S4) is quite similar to those of other members of the family Pachybolidae (e.g. compare to figures in [37]). The vulva in *Aphistogoniulus* is of a bivalve shape, consisting of an anterior and posterior valve (Supporting Information S4). Where both valves meet, two or three rows of setae are present. The area located basally of the plates is covered with the operculum. There is a noticeable intra-generical difference in the vulval shape between both investigated species. The anterior and posterior valves are of similar size in *A. jeekeli* n. sp. (Supporting Information S4), while the posterior valve is slightly larger in *A. erythrocephalus* (Supporting Information S4). The anterior valve is apically bulged in *A. erythrocephalus*, which is absent in *A. jeekeli*.

*The endotergum:* This character is generally overlooked in millipede studies, with the except of a few studies in the order Julida, Polydesmida and Chordeumatida. Similar structures on the tergite underside are coined 'endotergum' in the order Sphaerotheriida [53]. In the Spirobolida family Pachybolidae, the endotergum is only known from the Australian genus *Austrostrophus*. The endotergum consists of dentate cuticular scales in *Aphistogoniulus*, with large differences between the species. In *A. cowani* (Supporting Information S4), *A. erythrocephalus* (Supporting Information S4), *A. sanguineus*, *A. rubrodorsalis* n. sp., *A. diabolicus*, *A. corallipes*, and *A. vampyrus* the endotergum consists of several rows of dentate cuticular scales located quite a distance away from the posterior margin, while the endotergum in *A. jeekeli* n. sp. consists of a single row of non-dentate scales located at the margin (Supporting Information S4). The endotergum in *A. sanguineus* is also unique in that both large rectangular and more elongated scales are present. These results show that the endotergum should be considered more often in Spirobolida systematics.
