# Supplementary material for: Mountain Refugia Play a Role in Soil Arthropod Speciation on Madagascar: A Case Study of the Endemic Giant Fire-Millipede Genus Aphistogoniulus
Source: PLoS One. 2011 Dec 6;6(12):e28035. doi: 10.1371/journal.pone.0028035 (PMC3232213; doi:10.1371/journal.pone.0028035)
Supplement: Supporting Information S5 — Character discussion. (DOC) [file pone.0028035.s005.doc]

**Supporting Information S5**: Character discussion

| 1 - **Incisura lateralis: (0) open; (1) closed**. The incisura lateralis is a notch in the margin of the head capsule where the capsule articulates with the internal tentorium [61]. A closed incisura lateralis is an apomorphy of the tribe Pachybolini [36].  2 - **Gnathochilarium, number and position of setae on each lamella lingualis: (0) two setae, behind each other; (1) two or more setae on distal edge**. The presence of setae on the distal edge of the gnathochilarium is an apomorphy of the tribe Pachybolini [36].  3 - **Vulva: (0) simple, bivalve-like; (1) kidney- or crescent-shaped**. A kidney-shaped vulva is an apomorphy of the tribe Pachybolini [365].  4 - **Vulva: (0) both valves meet in a straight, regular fissure; (1) strongly sinuous fissure**. The presence of a sinuous fissure is an apomorphy of the tribe Pachybolini [36.  5 - **Vulva: (0) ridge, regular, not protruding; (1) strongly protruding**. A ridge is an apomorphy of the tribe Pachybolini [36].  6 - **Telson, preanal ring, (0) not protruding beyond anal valves; (1) protruding beyond anal valves**. The preanal rings in some genera of Spirobolida strongly protrude above the anal valves, likely an ecological adaptation.  7 - **Legs, male tarsal pads, (0) absent; (1) present on legs three to midbody legs; (2) present in legs three to last pair**. Male tarsal pads help the male when riding on the female for courtship behavior.  8 - **Male coxa 3, process (0) absent; (1) present**. Numerous species of Spirobolida possess coxite processes on their pregonopod legs. Their function is unknown.  9 - **Male coxa 4, process (0) absent; (1) present**. See above for a discussion of this character.  10 - **Male coxa 5, process (0) absent; (1) present**. See above for a discussion of this character.  11 - **Male coxa 6, process (0) absent; (1) present**. See above for a discussion of this character.  12 - **Male coxa 7, process (0) absent; (1) present**. See above for a discussion of this character.  13 - **Anterior gonopod (AG), retrorse process of telopodite appendage, (0) strongly developed; (1) absent**. A strongly developed retrorse process on the telopodite is found in the genera *Aphistogoniulus* (Fig. 7C, 8C), *Corallobolus* and several other Spirobolida genera. A very small, spine-like process is present in some *Colossobolus* species, while a retrorse process is absent in *Sanguinobolus*.  14 - **AG, telopodite, basally narrowed and apically strongly swollen knob-like appendage, (0) absent; (1) present**. Such a conspicuous swollen appendage is only found in the genus *Sanguinobolus* [37].  15 - **AG, coxite process (0) short and wide; (1) elongated**. An elongated coxite process is found in *Aphistogoniulus* (Figure 6A) and *Corallobolus*, while in *Sanguinobolus* and *Colossobolus* the coxite process short and wide. Either an elongated, or a short and wide process is present in numerous genera of Spirobolida.  16 - **AG telopodite, vertical location of large retrorse process and tip of appendage in (0) not recessed; (1) recessed**. In *Corallobolus* and most other genera of Spirobolida, the retrorse process is vertically on the same level as the appendage, while the retrorse process in *Aphistogoniulus* is located behind the level of the tip (Figs 7C, 8C).  17 - **AG, appendage, curvature towards lateral margin, (0) absent; (1) present**. The swollen appendage is suspiciously curved towards the lateral margin in some *Aphistogoniulus* species (Fig. 6C).  18 - **AG, mesal margin of appendage, (0) regular; (1) protruding**. The mesal margin of the appendage is projecting in *Colossobolus* species [37].  19 - **Posterior gonopod (pg), coxite, (0) not elongated; (1) restiform elongated**. The restiform shape of the posterior gonopod coxite can be found within the order Spirobolida only in the genus *Aphistogoniulus* and the recently described genera *Colossobolus* and *Sanguinobolus* [37].  20 - **PG, telopodite disc, (0) absent; (1) present**. A disc-shaped telopodite can only be found in *Aphistogoniulus* (Figs 6B, 7B), *Sanguinobolus* and *Colossobolus* [36].  21 - **PG, disc-shaped telopodite basally divided into two branches with sperm canal running through (0) mesal branch; (1) lateral branch**. The sperm canal running through the mesal branch of the telopodite of the posterior gonopods can only be found in the genera *Aphistogoniulus* (Fig. 7B) and *Colossobolus* [37]. The sperm canal runs through the lateral branch in *Sanguinobolus*. This character is not applicable to species where the telopodite of the posterior gonopods is not shaped like a disc  22 - **PG, disc-shaped telopodite, swollen membranous area between mesal and lateral branches, (0) absent; (1) present**. A membranous area between the lateral and mesal branches of the posterior gonopod telopodite is only present in the recently described genus *Colossobolus* [37]. This character is not applicable to species where the telopodite of the posterior gonopods is not shaped like a disc.  23 - **PG, mesal and lateral branches of disc-shaped telopodite forming (0) curved, branches non-parallel, forming a 'C' or almost an 'O'; (1) parallel to each other, forming a 'U'**. A clearly shaped C or O and a wide space between the two branches of the telopodite can be found in members of the genus *Aphistogoniulus* (Fig. 7D, 8D). A more compressed form of a 'U' can be found in genera *Colossobolus* and *Sanguinobolus* [37]. The telopodite branches of *A. infernalis* could be interpreted as a U, but is scored here as an 'O' because the apical part curves towards each other and more closely resemble telopodite branches of other *Aphistogoniulus* species. This character is not applicable to species where the telopodite of the posterior gonopods is not shaped like a disc.  24 - **PG, main branch, lateral membranous fringe (mfr) (0) absent or not projecting; (1) strongly developed into erect process**. Such a conspicuous fringe is only found in *A. cowani*, *A. sanguineus* and *A. rubrodorsalis* (Fig. 7B).  25 - **PG, telopodite, main branch, retrorse projection on anterior side (0) absent; (1) present**. This retrorse projection on the main branch is only present in *A. cowani*, *A. sanguineus*, *A. rubrodorsalis* (Fig. 7B), *A. erythrocephalus*, and *A. hova* [see 34].  26 - **PG, telopodite, tip of main branch (0) not folded; (1) folded**. The tip of the main branch is folded backwards only in *A. cowani*, *A. sanguineus* and *A. rubrodorsalis* (Fig. 7D).  27 - **PG, telopodite, (0) main branch < basal branch; (1) main branch > basal branch**. A short basal branch is found in *A. cowani*, *A. sanguineus*, *A. rubrodorsalis* (Fig. 7B) and *A. hova*. This character is only applicable for *Aphistogoniulus*, because of an unclear homology of the gonopod branches.  28 - **PG telopodite, basal branch, mesal spines, (0) absent; (1) present**. One mesal spine on the basal branch is present in *A. vampyrus* ([34]: fig. 20) and *A. infernalis* ([34]: fig. 15C). In *A. aridus* two mesal spines are found ([34]: fig. 18B).  29 - **PG, telopodite, basal branch shaped like a reaping hook (0) absent; (1) present**. The basal branch of the posterior gonopod telopodite is only shaped like a reaping hook in *A. diabolicus*, *A. infernalis*, *A. aridus*, *A. vampyrus* and *A. jeekeli* (Fig. 8B).  30 - **PG, telopodite, main branch, apical mesal fringe (0) absent; (1) present**. A mesal fringe on the main branch is only present in *A. infernalis*, *A. aridus*, *A. vampyrus* and *A. jeekeli* (Fig. 8D). This character is only applicable for *Aphistogoniulus*, because of an unclear homology of the membranous fringes.  31 - **PG, telopodite, basal branch, latero-basal swelling (0) absent; (1) present**. A conspicuous swelling at the basal branch is present only in *A. infernalis* ([34]: fig. 15B).  32 - **PG, telopodite, tip of main branch, large membranous sulcate fringe (0) absent; (1) present**. Such an intricate membranous fringe on the tip of the main branch is only present in *A. erythrocephalus*, and *A. hova* (Fig. S2C).  33 - **PG, telopodite, apical tip of basal branch, (0) well-rounded; (1) sharp-edged**. The tip of the basal branch is sharp-edged in *A. infernalis*, *A. diabolicus*, *A. aridus*, *A. vampyrus* and *A. jeekeli* (Fig. 7B), while the basal branch is rounded in *A. cowani*, *A. erythrocephalus*, A*. corallipes*, *A. hova*, *A. sanguineus*, *A. infernalis* and *A. rubrodorsalis* (Fig. 7B). This character is not applicable for the outgroup because of an unclear homology of the telopodite branches.  34 - **PG, telopodite, basal branch, rounded lobe on posterior side (0) absent; (1) present**. This rounded projection on the lateral side of the basal branch is only present in *A. jeekeli* (Fig. 8D) and absent in the other species of *Aphistogoniulus*.  35 - **PG, telopodite, bi-lobed apical tip of main branch (0) absent; (1) present**. The tip of the main branch is bi-lobed only in *A. vampyrus* and *A. aridus*. |
| --- |
