# Supplementary material for: Mountain Refugia Play a Role in Soil Arthropod Speciation on Madagascar: A Case Study of the Endemic Giant Fire-Millipede Genus Aphistogoniulus
Source: PLoS One. 2011 Dec 6;6(12):e28035. doi: 10.1371/journal.pone.0028035 (PMC3232213; doi:10.1371/journal.pone.0028035)
Supplement: Supporting Information S6 — Character matrix as nexus file. (DOC) [file pone.0028035.s006.doc]

**Supporting Information S6**: Character matrix as nexus file

#NEXUS

[written Thu Feb 11 15:06:16 CST 2010 by Mesquite version 2.6 (build 486) at Darwin/10.10.33.108]

BEGIN TAXA;

TITLE Taxa;

DIMENSIONS NTAX=18;

TAXLABELS

Epibolus_pulchripes Madabolus_maximus Corallobolus_cruentus Sanguinobolus_maculosus Colossobolus_semicyclus Colossoblus_oblongopedus A_cowani A_erythrocephalus A_hova A_corallipes A_sanguineus A_infernalis A_diabolicus A_aridus A_vampyrus 'A_rubrodorsalis' A_sp_12299 'A_ignipes'

;

END;

BEGIN CHARACTERS;

TITLE Character_Matrix;

DIMENSIONS NCHAR=35;

FORMAT DATATYPE = STANDARD GAP = - MISSING = ? SYMBOLS = " 0 1 2";

CHARSTATELABELS

1 'Incisura lateralis: (0) open; (1) closed', 2 'Gnathochilarium, number and position of setae on each lamella lingualis: (0) two setae, behind each other; (1) two or more setae on distal edge. ', 3 'Vulva: simple, bivalve-like (0); kidney- or crescent-shaped (1)', 4 'Vulva: both valves meet in a straight, regular fissure (0); strongly sinuous fissure (1)', 5 'Vulva: ridge (sensu Brolemann, 1922), regular, not protruding (0); strongly protruding (1)', 6 'Telson, preanal ring, (0) not protruding above anal valves; (1) protruding above anal valves', 7 'Legs, male tarsal pads, (0) absent; (1) present on legs three to midbody legs; (2) present in legs three to last pair', 8 'Male coxa 3, process (0) absent; (1) present', 9 'Male coxa 4, process (0) absent; (1) present', 10 'Male coxa 5, process (0) absent; (1) present.', 11 'Male coxa 6, process (0) absent; (1) present', 12 'Male coxa 7, process (0) absent; (1) present', 13 'Anterior gonopod (AG), retrorse process of telopodite appendage, (0) strongly developed; (1) absent.', 14 'AG telopodite, basally narrowed and apically strongly swollen knob-like appendage, (0) absent; (1) present', 15 'AG, coxite process (0) short and wide; (1) elongated', 16 'AG telopodite, vertical location of large retrorse process and tip of appendage in (0) not recessed; (1) recessed', 17 'AG, appendage, curvature towards lateral margin, absent (0); present (1)', 18 'AG, mesal margin of appendage, regular (0); protruding (1)', 19 'Posterior gonopod (pg), coxite, (0) not elongated; (1) restiform elongated', 20 'PG, telopodite disc, (0) absent; (1) present', 21 'PG, disc-shaped telopodite basally divided into two branches with sperm canal running through (0) mesal branch; (1) lateral branch', 22 'PG, disc-shaped telopodite, swollen membranous area between mesal and lateral branches, (0) absent; (1) present', 23 'PG, mesal and lateral branches of disc-shaped telopodite forming (0) non-parallel, curved, forming a C or almost an O; (1) parallel to each other, forming a ''U''', 24 'PG, main branch, lateral membranous fringe (mfr) (0) absent or not projecting; (1) strongly developed into erect process', 25 'PG telopodite, main branch, retrorse projection on anterior side (0) absent; (1) present', 26 'PG, telopodite, tip of main branch (0) not folded; (1) folded', 27 'PG, telopodite, (0) main branch < basal branch; (1) main branch > basal branch', 28 'PG telopodite, basal branch, mesal spines, (0) absent; (1) present;', 29 'PG, telopodite, basal branch shaped like a reaping hook (0) absent; (1) present', 30 'PG, telopodite, main branch, apical mesal fringe (0) absent; (1) present', 31 'PG, telopodite, basal branch, latero-basal swelling (0) absent; (1) present', 32 'PG, telopodite, tip of main branch, large membraneous sulcate fringe (0) absent; (1) present', 33 'PG, telopodite, apical tip of basal branch, (0) pointed; (1) rounded;', 34 'PG, telopodite, basal branch, rounded lobe on posterior side (0) absent; (1) present', 35 'PG, telopodite, bi-lobed apical tip of main branch (0) absent; (1) present' ;

MATRIX

Epibolus_pulchripes 11111020000000000000---000-00-00-00

Madabolus_maximus 11111020000000000000---000-00-00-00

Corallobolus_cruentus 00000110000000000000---000-00-00-00

Sanguinobolus_maculosus 00000021100011000011101000-00-00-00

Colossobolus_semicyclus 00000021111110000111011000-00-00-00

Colossoblus_oblongopedus 00000021111110000111011000-00-00-00

A_cowani 00000020000000110011000111100000000

A_erythrocephalus 00000020000000110011000010100001000

A_hova 00???020000000110011000010100001000

A_corallipes 00???020000000111011000000100000000

A_sanguineus 00???020000000110011000111100000000

A_infernalis 00???020000000111011000000011110100

A_diabolicus 00???020000000111011000000001100100

A_aridus 00???020000000111011000000011100101

A_vampyrus 00???020000000111011000000011100101

'A_rubrodorsalis' 00???020000000110011000111100000000

A_sp_12299 00000020000000111011000000001100110

'A_ignipes' 00???020000000110011000010100001000

;

END;

BEGIN ASSUMPTIONS;

TYPESET * UNTITLED = unord: 1 - 35;

END;

BEGIN MESQUITECHARMODELS;

ProbModelSet * UNTITLED = 'Mk1 (est.)': 1 - 35;

END;

Begin MESQUITE;

MESQUITESCRIPTVERSION 2;

TITLE AUTO;

tell ProjectCoordinator;

getEmployee #mesquite.minimal.ManageTaxa.ManageTaxa;

tell It;

setID 0 1769809915475985573;

endTell;

getEmployee #mesquite.charMatrices.ManageCharacters.ManageCharacters;

tell It;

setID 0 8220776410383534510;

tell It;

setDefaultOrder 0 1 2 3 4 5 6 7 8 9 10 11 17 18 20 19 34 35 12 13 14 15 16 22 23 24 25 26 27 28 29 30 31 32 33;

attachments ;

endTell;

checksumv 0 2 2821964619 null;

endTell;

getWindow;

tell It;

suppress;

setResourcesState false false 100;

setPopoutState 400;

setExplanationSize 0;

setAnnotationSize 0;

setFontIncAnnot 0;

setFontIncExp 0;

setSize 1028 690;

setLocation -4 0;

setFont SanSerif;

setFontSize 10;

getToolPalette;

tell It;

endTell;

desuppress;

endTell;

getEmployee #mesquite.minimal.ManageTaxa.ManageTaxa;

tell It;

showTaxa #1769809915475985573 #mesquite.lists.TaxonList.TaxonList;

tell It;

setTaxa #1769809915475985573;

getWindow;

tell It;

newAssistant #mesquite.lists.DefaultTaxaOrder.DefaultTaxaOrder;

newAssistant #mesquite.lists.TaxonListCurrPartition.TaxonListCurrPartition;

getTable;

tell It;

rowNamesWidth 134;

endTell;

setExplanationSize 30;

setAnnotationSize 20;

setFontIncAnnot 0;

setFontIncExp 0;

setSize 928 623;

setLocation -4 0;

setFont SanSerif;

setFontSize 10;

getToolPalette;

tell It;

setTool mesquite.lists.TaxonList.TaxonListWindow.ibeam;

endTell;

endTell;

showWindow;

getEmployee #mesquite.lists.ColorTaxon.ColorTaxon;

tell It;

setColor Red;

removeColor off;

endTell;

getEmployee #mesquite.lists.TaxonListAnnotPanel.TaxonListAnnotPanel;

tell It;

togglePanel off;

endTell;

endTell;

endTell;

getEmployee #mesquite.charMatrices.BasicDataWindowCoord.BasicDataWindowCoord;

tell It;

showDataWindow #8220776410383534510 #mesquite.charMatrices.BasicDataWindowMaker.BasicDataWindowMaker;

tell It;

getWindow;

tell It;

setExplanationSize 30;

setAnnotationSize 20;

setFontIncAnnot 0;

setFontIncExp 0;

setSize 928 623;

setLocation -4 0;

setFont SanSerif;

setFontSize 10;

getToolPalette;

tell It;

setTool mesquite.charMatrices.BasicDataWindowMaker.BasicDataWindow.ibeam;

endTell;

setActive;

setTool mesquite.charMatrices.BasicDataWindowMaker.BasicDataWindow.ibeam;

colorCells #mesquite.charMatrices.ColorByState.ColorByState;

colorRowNames #mesquite.charMatrices.TaxonGroupColor.TaxonGroupColor;

colorColumnNames #mesquite.charMatrices.CharGroupColor.CharGroupColor;

colorText #mesquite.charMatrices.NoColor.NoColor;

setBackground White;

toggleShowNames on;

toggleShowTaxonNames on;

toggleTight off;

toggleThinRows off;

toggleShowChanges on;

toggleSeparateLines off;

toggleShowStates on;

toggleAutoWCharNames on;

toggleShowDefaultCharNames off;

toggleConstrainCW on;

toggleBirdsEye off;

toggleAllowAutosize on;

toggleColorsPanel off;

toggleDiagonal on;

setDiagonalHeight 80;

toggleLinkedScrolling on;

toggleScrollLinkedTables off;

endTell;

showWindow;

getWindow;

tell It;

forceAutosize;

endTell;

getEmployee #mesquite.charMatrices.ColorCells.ColorCells;

tell It;

setColor Red;

removeColor off;

endTell;

getEmployee #mesquite.categ.StateNamesStrip.StateNamesStrip;

tell It;

showStrip off;

endTell;

getEmployee #mesquite.charMatrices.AnnotPanel.AnnotPanel;

tell It;

togglePanel off;

endTell;

getEmployee #mesquite.charMatrices.CharReferenceStrip.CharReferenceStrip;

tell It;

showStrip off;

endTell;

getEmployee #mesquite.charMatrices.QuickKeySelector.QuickKeySelector;

tell It;

autotabOff;

endTell;

getEmployee #mesquite.categ.SmallStateNamesEditor.SmallStateNamesEditor;

tell It;

panelOpen true;

endTell;

endTell;

endTell;

endTell;

end;
