# Supplementary material for: Mountain Refugia Play a Role in Soil Arthropod Speciation on Madagascar: A Case Study of the Endemic Giant Fire-Millipede Genus Aphistogoniulus
Source: PLoS One. 2011 Dec 6;6(12):e28035. doi: 10.1371/journal.pone.0028035 (PMC3232213; doi:10.1371/journal.pone.0028035)
Supplement: Supporting Information S7 — Alignment of the combined (16S+CO1+18S) dataset as nexus file. (DOC) [file pone.0028035.s007.doc]

**Supporting Information S7**: Alignment of the combined (16S+CO1+18S) dataset as nexus file.

#NEXUS

[D:\Artikel\Arbeit\PETER_~1\MOLECU~1\Nexus\COMBIN~2.NEX -- data title]

[Name: Doratogonus Len: 3031 Check: 0]

[Name: Mad_maxASP107_18S Len: 3031 Check: 0]

[Name: Mad_maxBSP108_18S Len: 3031 Check: 0]

[Name: S_simplexSP078_18S Len: 3031 Check: 0]

[Name: S_triareusSP080_18S Len: 3031 Check: 0]

[Name: C_AsemiSP073_Co1 Len: 3031 Check: 0]

[Name: C_BsemiSP074_18S Len: 3031 Check: 0]

[Name: A_AvampyrusSP119_18S Len: 3031 Check: 0]

[Name: A_BvampyrusSP120_18S Len: 3031 Check: 0]

[Name: A_CvampyrusSP121_18S Len: 3031 Check: 0]

[Name: A_DvampyrusSP122_18S Len: 3031 Check: 0]

[Name: A_AinfernalisSP058_18S Len: 3031 Check: 0]

[Name: A_BinfernalisSP059_18S Len: 3031 Check: 0]

[Name: A_CinfernalisSP060_18S Len: 3031 Check: 0]

[Name: A_DinfernalisSP061_18S Len: 3031 Check: 0]

[Name: A_EinfernalisSP071_18S Len: 3031 Check: 0]

[Name: A_AcorallipesSP062_18S Len: 3031 Check: 0]

[Name: A_BcorallipesSP063_18S Len: 3031 Check: 0]

[Name: A_nsp12299SP069_18S Len: 3031 Check: 0]

[Name: A_nsp13962SP070_18S Len: 3031 Check: 0]

[Name: A_AerythrocephalusSP065Co1 Len: 3031 Check: 0]

[Name: A_BerythrocephalusSP066_Co1 Len: 3031 Check: 0]

[Name: A_AhovaSP067_18S Len: 3031 Check: 0]

[Name: A_BhovaSP068_18S Len: 3031 Check: 0]

[Name: A_ignipesSP092_18S Len: 3031 Check: 0]

[Name: A_BcowaniSP112_18S Len: 3031 Check: 0]

[Name: A_CcowaniSP113_Co1 Len: 3031 Check: 0]

[Name: A_DcowaniSP114_18S Len: 3031 Check: 0]

[Name: A_AsanguineusSP115_Co1 Len: 3031 Check: 0]

[Name: A_BsanguineusSP116_Co1 Len: 3031 Check: 0]

[Name: A_CsanguineusSP117_Co1 Len: 3031 Check: 0]

[Name: A_DsanguineusSP118_Co1 Len: 3031 Check: 0]

begin data;

dimensions ntax=32 nchar=3031;

format datatype=dna interleave missing=-;

matrix

Doratogon --TCATATGCTTGTCTCAAA GATTAAGCCATGCATGTCTA AGTACATACCTTAAAAAG-- --GTGAAACCGCGAATGGCT CATTAAATCAGTCATGGTTT

Mad_maxAS --TCATATGCTTGTTTCAAA GATTAAGCCATGCATGCCTA AGTAC-TAACTCAATTAGCA CAGTGAAACCGCAGATGGCT CATTAAATCAGTCACTATTT

Mad_maxBS --TCATATGCTTGTTTCAAA GATTAAGCCATGCATGCCTA AGTAC-TAACTCAATTAGCA CAGTGAAACCGCAGATGGCT CATTAAATCAGTCACTATTT

S_simplex AATTATATGCTTGTCTCAAA GATTAAGCCATGCATGTCTA AGTAC-TAACTCAA--TGAA AAGTGAAACCGCAAATGGCT CATTAAATCAGTCACTATTT

S_triareu AATTATATGCTTGTCTCAAA GATTAAGCCATGCATGTCTA TGTAC-TAACTCAA--TGAA AAGTGAAACCGCAAATGGCT CATTAAATCAGTCACTATTT

C_AsemiSP nnnnnnnnnnnnnnnnnnnn nnnnnnnnnnnnnnnnnnnn nnnnnnnnnnnnnnnnnnnn nnnnnnnnnnnnnnnnnnnn nnnnnnnnnnnnnnnnnnnn

C_BsemiSP AATCATACGCTTGTTTCAAA GATTAAGCCATGCATGTCTA AGTAC-TAACTCAA-AAGCA GAGTGAAACCGCAAATGGCT CATTAAATCAGTCACGATGA

A_Avampyr AATCATATGCTTGTTTCAAA GATTAAGCCATGCATGTCTA AGTAC-TAACTCGA-AAGAC TAGTGAAACCGCAAATGGCT CATTAAATCAGTGACGATTC

A_Bvampyr AATCATATGCTTGTTTCAAA GATTAAGCCATGCATGTCTA AGTAC-TAACTCGA-AAGAC CAGTGAAACCGCAAATGGCT CATTAAATCAGTCACGATTT

A_Cvampyr AATCATATGCTTGTTTCAAA GATTAAGCCATGCATGTCTA AGTAC-TAACTCGA-AAGAC CAGTGAAACCGCAAATGGCT CATTAAATCAGTCACGATTT

A_Dvampyr AATCATATGCTTGTTTCAAA GATTAAGCCATGCATGTCTA AGTAC-TAACTCGA-AAGAC TAGTGAAACCGCAAATGGCT CATTAAATCAGTGACGATTC

A_Ainfern AATCATATGCTTGTTTCAAA GATTAAGCCATGCATGTCTA AGTAC-TAACTCGA-AAGAC TAGTGAAACCGCAAATGGCT CATTAAATCAGTCACGATTT

A_Binfern AATCATATGCTTGTTTCAAA GATTAAGCCATGCATGTCTA AGTAC-TAACTCGA-AAGAC TAGTGAAACCGCAAATGGCT CATTAAATCAGTCACGATTT

A_Cinfern AATCATATGCTTGTTTCAAA GATTAAGCCATGCATGTCTA AGTAC-TAACTCGA-AAGAC TAGTGAAACCGCAAATGGCT CATTAAATCAGTCACGATTT

A_Dinfern AATCATATGCTTGTTTCAAA GATTAAGCCATGCATGTCTA AGTAC-TAACTCGA-AAGAC TAGTGAAACCGCAAATGGCT CATTAAATCAGTCACGATTT

A_Einfern AATCATATGCTTGTTTCAAA GATTAAGCCATGCATGTCTA AGTAC-TAACTCGA-AAGAC TAGTGAAACCGCAAATGGCT CATTAAATCAGTCACGATTT

A_Acorall AATCATACGCTTGTTTCAAA GATTAAGCCATGCATGTCTA AGTAC-TAACTCGA-AAGAC TAGTGAAACCGCAAATGGCT CATTAAATCAGTCACGATTT

A_Bcorall AATCATACGCTTGTTTCAAA GATTAAGCCATGCATGTCTA AGTAC-TAACTCGA-AAGAC TAGTGAAACCGCAAATGGCT CATTAAATCAGTCACGATTT

A_nsp1229 AATCATATGCTTGTTTCAAA GATTAAGCCATGCATGTCTA AGTAC-TAACTCGA-AAGAC TAGTGAAACCGCAAATGGCT CATTAAATCAGTCACGATTT

A_nsp1396 AATCATATGCTTGTTTCAAA GATTAAGCCATGCATGTCTA AGTAC-TAACTCGA-AAGAA GAGTGAAACCGCAAATGGCT CATTAAATCAGTCACGATTT

A_Aerythr nnnnnnnnnnnnnnnnnnnn nnnnnnnnnnnnnnnnnnnn nnnnnnnnnnnnnnnnnnnn nnnnnnnnnnnnnnnnnnnn nnnnnnnnnnnnnnnnnnnn

A_Berythr nnnnnnnnnnnnnnnnnnnn nnnnnnnnnnnnnnnnnnnn nnnnnnnnnnnnnnnnnnnn nnnnnnnnnnnnnnnnnnnn nnnnnnnnnnnnnnnnnnnn

A_AhovaSP AATCATACGCTTGTTTCAAA GATTAAGCCATGCATGTCTA AGTAC-TAACTCGA-AAGAC TAGTGAAACCGCAAATGGCT CATTAAATCAGTCTCGATTT

A_BhovaSP AATCATACGCTTGTTTCAAA GATTAAGCCATGCATGTCTA AGTAC-TAACTCGA-AAGAC TAGTGAAACCGCAAATGGCT CATTAAATCAGTCTCGATTT

A_ignipes AATCATACGCTTGTTTCAAA GATTAAGCCATGCATGTCTA AGTAC-TAACTCGA-AAGAC TAGTGAAACCGCAAATGGCT CATTAAATCAGTCTCGATTT

A_Bcowani AATCATACGCTTGTTACAAA GATTAAGCCATGCATGTCTA AGTAC-TAACTCGA-AAGAA TAGTGAAACCGCAAATGGCT CATTAAATCAGTCTCGATCT

A_Ccowani nnnnnnnnnnnnnnnnnnnn nnnnnnnnnnnnnnnnnnnn nnnnnnnnnnnnnnnnnnnn nnnnnnnnnnnnnnnnnnnn nnnnnnnnnnnnnnnnnnnn

A_Dcowani AATCATACGCTTGTTACAAA GATTAAGCCATGCATGTCTA AGTAC-TAACTCGA-AAGAA TAGTGAAACCGCAAATGGCT CATTAAATCAGTCTCGATCT

A_Asangui nnnnnnnnnnnnnnnnnnnn nnnnnnnnnnnnnnnnnnnn nnnnnnnnnnnnnnnnnnnn nnnnnnnnnnnnnnnnnnnn nnnnnnnnnnnnnnnnnnnn

A_Bsangui nnnnnnnnnnnnnnnnnnnn nnnnnnnnnnnnnnnnnnnn nnnnnnnnnnnnnnnnnnnn nnnnnnnnnnnnnnnnnnnn nnnnnnnnnnnnnnnnnnnn

A_Csangui nnnnnnnnnnnnnnnnnnnn nnnnnnnnnnnnnnnnnnnn nnnnnnnnnnnnnnnnnnnn nnnnnnnnnnnnnnnnnnnn nnnnnnnnnnnnnnnnnnnn

A_Dsangui nnnnnnnnnnnnnnnnnnnn nnnnnnnnnnnnnnnnnnnn nnnnnnnnnnnnnnnnnnnn nnnnnnnnnnnnnnnnnnnn nnnnnnnnnnnnnnnnnnnn

Doratogon ACTAGACCGATCAATCCTAC TTGGATAACTGTGGTAATTC TAGAGCTAATACATGCCAAG AT-GCTCCTACCCTC----G TGGTTCGAGCGCATTTATTA

Mad_maxAS ACAAGA-GACTCAATCATAG TTGGATAACTTTGGAAAATC TAGAGCTAATACATGCTTCA AGTTCTCGCACCACC-TCGG TGGGACGAGCGCACTTATTA

Mad_maxBS ACAAGA-GACTCAATCATAG TTGGATAACTTTGGAAAATC TAGAGCTAATACATGCTTCA AGTTCTCGCACCACC-TCGG TGGGACGAGCGCACTTATTA

S_simplex ACTAGA--CAGTGCT-TGCC TTGGATAACTTTGGCAATTC TAGAGCTAATACATGCCTTG AG-TCTCGGACCCTT---CG GGGGACGAGCGCACTTATTA

S_triareu GCTAGA--CATTGCTTTGCC TTGGATAACTTTGGCAATTC TAGAGCTAATACATGCCTTG AG-TCTCGGACCCTT--CGT GGGGACGAGCGCACTTATTA

C_AsemiSP nnnnnnnnnnnnnnnnnnnn nnnnnnnnnnnnnnnnnnnn nnnnnnnnnnnnnnnnnnnn nnnnnnnnnnnnnnnnnnnn nnnnnnnnnnnnnnnnnnnn

C_BsemiSP GCAGGA--CATGCACATGCC TCGGATAACCTTGGCAATTC TAGAGCTAATACATGCCATG AG-TCTCGGACCCCTCTCAG GGGGACGAGCGCACTTATTA

A_Avampyr GCTAGA--CATGTTC-TGCC TTGGATAACTTTGGCAATTC TAGAGCTAATACATGCCTCG AG-TCTCGGACCTTC----- GGGGACGAGCGCAATTATTA

A_Bvampyr GCTAGA--CATGTTC-TGCC TTGGATAACTTTGGCAATTC TAGAGCTAATACATGCCACG AG-TCTCGGACCTTC----- GGGGACGAGCGCAATTATTA

A_Cvampyr GCTAGA--CATGTTC-TGCC TTGGATAACTTTGGCAATTC TAGAGCTAATACATGCCACG AG-TCTCGGACCTTC----- GGGGACGAGCGCAATTATTA

A_Dvampyr GCTAGA--CATGTTC-TGCC TTGGATAACTTTGGCAATTC TAGAGCTAATACATGCCTCG AG-TCTCGGACCTTC----- GGGGACGAGCGCAATTATTA

A_Ainfern GCTAGA--CATGTTC-TGCC TTGGATAACTTTGGCAATTC TAGAGCTAATACATGCCTCG AG-TCTCGGACCTTC----- GGGGACGAGCGCAATTATTA

A_Binfern GCTAGA--CATGTTC-TGCC TTGGATAACTTTGGCAATTC TAGAGCTAATACATGCCTCG AG-TCTCGGACCTTC----- GGGGACGAGCGCAATTATTA

A_Cinfern GCTAGA--CATGTTC-TGCC TTGGATAACTTTGGCAATTC TAGAGCTAATACATGCCTCG AG-TCTCGGACCTTC----- GGGGACGAGCGCAATTATTA

A_Dinfern GCTAGA--CATGTTC-TGCC TTGGATAACTTTGGCAATTC TAGAGCTAATACATGCCTCG AG-TCTCGGACCTTC----- GGGGACGAGCGCAATTATTA

A_Einfern GCTAGA--CATGTTC-TGCC TTGGATAACTTTGGCAATTC TAGAGCTAATACATGCCTCG AG-TCTCGGACCTTC----- GGGGACGAGCGCAATTATTA

A_Acorall ACTAGA--CATGTTC-TGCC TTGGATAACTTTGGCAATTC TAGAGCTAATACATGCCTTG AG-TCTCGGACCTTC----- GGGGACGAGCGCAATTATTA

A_Bcorall ACTAGA--CATGTTC-TGCC TTGGATAACTTTGGCAATTC TAGAGCTAATACATGCCTTG AG-TCTCGGACCTTC----- GGGGACGAGCGCAATTATTA

A_nsp1229 GCTAGA--CATGTTC-TGCC TTGGATAACTTTGGCAATTC TAGAGCTAATACATGCCTTG AG-TCTCGGACCTTC----- GGGGACGAGCGCAATTATTA

A_nsp1396 GCTAGA--CATGTTC-TGCC TTGGATAACTTTGGCAATTC TAGAGCTAATACATGCCTTG AG-TCTCGGACCTTC----- GGGGACGAGCGCAATTATTA

A_Aerythr nnnnnnnnnnnnnnnnnnnn nnnnnnnnnnnnnnnnnnnn nnnnnnnnnnnnnnnnnnnn nnnnnnnnnnnnnnnnnnnn nnnnnnnnnnnnnnnnnnnn

A_Berythr nnnnnnnnnnnnnnnnnnnn nnnnnnnnnnnnnnnnnnnn nnnnnnnnnnnnnnnnnnnn nnnnnnnnnnnnnnnnnnnn nnnnnnnnnnnnnnnnnnnn

A_AhovaSP -TTAGA--CATAGTC-TGCC TTGGATAACTATGGCAATTC TAGAGCTAATACATGCCTTG AG-TCTCGGACCTTC----- GGGGACGAGCGCAATTATTA

A_BhovaSP -TTAGA--CATAGTC-TGCC TTGGATAACTATGGCAATTC TAGAGCTAATACATGCCTTG AG-TCTCGGACCTTC----- GGGGACGAGCGCAATTATTA

A_ignipes -TTAGA--CATAGTC-TGCC TTGGATAACTATGGCAATTC TAGAGCTAATACATGCCTTG AG-TCTCGGACCTTC----- GGGGACGAGCGCAATTATTA

A_Bcowani -TTAGA--CATTGTC-TGCC TTGGATAACTTTGGCAATTC TAGAGCTAATACATGCCTTG AG-TCTCGGACCTTC----- GGGGACGAGCGCAATTATTA

A_Ccowani nnnnnnnnnnnnnnnnnnnn nnnnnnnnnnnnnnnnnnnn nnnnnnnnnnnnnnnnnnnn nnnnnnnnnnnnnnnnnnnn nnnnnnnnnnnnnnnnnnnn

A_Dcowani -TTAGA--CATTGTC-TGCC TTGGATAACTTTGGCAATTC TAGAGCTAATACATGCCTTG AG-TCTCGGACCTTC----- GGGGACGAGCGCAATTATTA

A_Asangui nnnnnnnnnnnnnnnnnnnn nnnnnnnnnnnnnnnnnnnn nnnnnnnnnnnnnnnnnnnn nnnnnnnnnnnnnnnnnnnn nnnnnnnnnnnnnnnnnnnn

A_Bsangui nnnnnnnnnnnnnnnnnnnn nnnnnnnnnnnnnnnnnnnn nnnnnnnnnnnnnnnnnnnn nnnnnnnnnnnnnnnnnnnn nnnnnnnnnnnnnnnnnnnn

A_Csangui nnnnnnnnnnnnnnnnnnnn nnnnnnnnnnnnnnnnnnnn nnnnnnnnnnnnnnnnnnnn nnnnnnnnnnnnnnnnnnnn nnnnnnnnnnnnnnnnnnnn

A_Dsangui nnnnnnnnnnnnnnnnnnnn nnnnnnnnnnnnnnnnnnnn nnnnnnnnnnnnnnnnnnnn nnnnnnnnnnnnnnnnnnnn nnnnnnnnnnnnnnnnnnnn

Doratogon GACCA-AAACCGATCGGGTG CCTCGGTGCCCGTCAGTTTG TGGTGACTCTGGATAACTTT GAGCAGA-TCGCAC-GGTC- TTTGCGCCGGCGACGTATCT

Mad_maxAS GGCCATAAA---ACCATTGC CCTTA------------AAA TGGCGATTCTGGATAAGAG- --GTAGACTCGTGTGGGTCG AAAGTACCAACGACATGTCT

Mad_maxBS GGCCATAAA---ACCATTGC CCTTA------------AAA TGGCGATTCTGGATAAGAG- --GTAGACTCGTGTGGGTCG AAAGTACCAACGACATGTCT

S_simplex GACCA-AAA---ACCAGTTA CCTGG------------CTA TGGTGACTCTAGATAAAAT- --GCCGATTCGCGCGGGTC- TT-GTACCAGCGACGAGTCT

S_triareu GACCA-AAA---ACCAGTTA CCTGG------------CTA TGGTGACTCTAGATAAATT- --GCCGATTCGCGCGGGTCA T--GTACCAGCGACGAGTCT

C_AsemiSP nnnnnnnnnnnnnnnnnnnn nnnnnnnnnnnnnnnnnnnn nnnnnnnnnnnnnnnnnnnn nnnnnnnnnnnnnnnnnnnn nnnnnnnnnnnnnnnnnnnn

C_BsemiSP GACCA-AAA---ACCAGTAT CCTGG------------CTA TGGTGACTCTAGATAAAATC --GCAGATTCGCGCGGGTC- TCAGCACCAGCGACGAGTCT

A_Avampyr GACCA-AAA---ACCAGTTA CCTGG------------CTA TGGTGACTCTAGATAAAAT- --GCAGATTCGCGCGGGTC- TC-GTACCAGCGACGAGTCT

A_Bvampyr GACCA-AAA---ACCAGTTA CCTGG------------CTA TGGTGACTCTAGATAAAAT- --GCAGATTCGCGCGGGTC- TC-GTACCAGCGACGAGTCT

A_Cvampyr GACCA-AAA---ACCAGTTA CCTGG------------CTA TGGTGACTCTAGATAAAAT- --GCAGATTCGCGCGGGTC- TC-GTACCAGCGACGAGTCT

A_Dvampyr GACCA-AAA---ACCAGTTA CCTGG------------CTA TGGTGACTCTAGATAAAAT- --GCAGATTCGCGCGGGTC- TC-GTACCAGCGACGAGTCT

A_Ainfern GACCA-AAA---ACCAGTTA CCTGG------------CTA TGGTGACTCTAGATAAAAT- --GCAGATTCGCGCGGGTC- TC-GTACCAGCGACGATTCT

A_Binfern GACCA-AAA---ACCAGTTA CCTGG------------CTA TGGTGACTCTAGATAAAAT- --GCAGATTCGCGCGGGTC- TC-GTACCAGCGACGATTCT

A_Cinfern GACCA-AAA---ACCAGTTA CCTGG------------CTA TGGTGACTCTAGATAAAAT- --GCAGATTCGCGCGGGTC- TC-GTACCAGCGACGATTCT

A_Dinfern GACCA-AAA---ACCAGTTA CCTGG------------CTA TGGTGACTCTAGATAAAAT- --GCAGATTCGCGCGGGTC- TC-GTACCAGCGACGATTCT

A_Einfern GACCA-AAA---ACCAGTTA CCTGG------------CTA TGGTGACTCTAGATAAAAT- --GCAGATTCGCGCGGGTC- TC-GTACCAGCGACGATTCT

A_Acorall GACCA-AAA---ACCAGTTA CCTGG------------CTA TGGTGACTCTAGATAAAAT- --GCAGATTCGCGCGGGTC- TC-GTACCAGCGACGAGTCT

A_Bcorall GACCA-AAA---ACCAGTTA CCTGG------------CTA TGGTGACTCTAGATAAAAT- --GCAGATTCGCGCGGGTC- TC-GTACCAGCGACGAGTCT

A_nsp1229 GACCA-AAA---ACCAGTTA CCTGG------------ATA TGGTGACTCTAGATAGAAT- --GCAGATTCGCGCAGGTC- TT-GTACCAGCGACGAGTCT

A_nsp1396 GACCA-AAA---ACCAGTTA CCTGG------------CTA TGGTGACTCTAGATAGAGT- --GCAGATTCGCGCGGGTC- TC-GTACCAGCGACGAGTCT

A_Aerythr nnnnnnnnnnnnnnnnnnnn nnnnnnnnnnnnnnnnnnnn nnnnnnnnnnnnnnnnnnnn nnnnnnnnnnnnnnnnnnnn nnnnnnnnnnnnnnnnnnnn

A_Berythr nnnnnnnnnnnnnnnnnnnn nnnnnnnnnnnnnnnnnnnn nnnnnnnnnnnnnnnnnnnn nnnnnnnnnnnnnnnnnnnn nnnnnnnnnnnnnnnnnnnn

A_AhovaSP GACCA-AAA---ACCAGTTA CCTGG------------CTA TGGTGACTCTAGATAAATT- --GCAGATTCGCGCGGGTC- TC-GTACCAGCGACGAGTCT

A_BhovaSP GACCA-AAA---ACCAGTTA CCTGG------------CTA TGGTGACTCTAGATAAATT- --GCAGATTCGCGCGGGTC- TC-GTACCAGCGACGAGTCT

A_ignipes GACCA-AAA---ACCAGTTA CCTGG------------CTA TGGTGACTCTAGATAAAAT- --GCAGATTCGCGCAGGTC- TC-GTACCAGCGACGAGTCT

A_Bcowani GACCA-AAA---ACCAGTAA CCTGG------------CTA TGGTGACTCTAGATAAATT- --GCAGATTCGCGCAGGTC- TC-GTACCGGCGACGAGTCT

A_Ccowani nnnnnnnnnnnnnnnnnnnn nnnnnnnnnnnnnnnnnnnn nnnnnnnnnnnnnnnnnnnn nnnnnnnnnnnnnnnnnnnn nnnnnnnnnnnnnnnnnnnn

A_Dcowani GACCA-AAA---ACCAGTAA CCTGG------------CTA TGGTGACTCTAGATAAATT- --GCAGATTCGCGCAGGTC- TC-GTACCGGCGACGAGTCT

A_Asangui nnnnnnnnnnnnnnnnnnnn nnnnnnnnnnnnnnnnnnnn nnnnnnnnnnnnnnnnnnnn nnnnnnnnnnnnnnnnnnnn nnnnnnnnnnnnnnnnnnnn

A_Bsangui nnnnnnnnnnnnnnnnnnnn nnnnnnnnnnnnnnnnnnnn nnnnnnnnnnnnnnnnnnnn nnnnnnnnnnnnnnnnnnnn nnnnnnnnnnnnnnnnnnnn

A_Csangui nnnnnnnnnnnnnnnnnnnn nnnnnnnnnnnnnnnnnnnn nnnnnnnnnnnnnnnnnnnn nnnnnnnnnnnnnnnnnnnn nnnnnnnnnnnnnnnnnnnn

A_Dsangui nnnnnnnnnnnnnnnnnnnn nnnnnnnnnnnnnnnnnnnn nnnnnnnnnnnnnnnnnnnn nnnnnnnnnnnnnnnnnnnn nnnnnnnnnnnnnnnnnnnn

Doratogon TTCAAATGTCTGCCTTATCA ACTGTCGATGGTAGGCTACT TGCCTACCATGGTTGTAACG GGTAACGGGGAATCAGGGTT CGATTCCGGAGAGGGAGCCT

Mad_maxAS TATAAGCGTCTGCCTTATCA ACTA--GATGGTAGGGTAAG GGCCTACCATGGTTGTGACG GGTGACGGGGAATCAGGGTT CGATTCCGGAGAGGCAGCCT

Mad_maxBS TATAAGCGTCTGCCTTATCA ACTA--GATGGTAGGGTAAG GGCCTACCATGGTTGTGACG GGTGACGGGGAATCAGGGTT CGATTCCGGAGAGGCAGCCT

S_simplex TATAAGCGTCTGCCTTATCA ACTA--GATGGTAGGGTAAC GGCCTACCATGGTTGTGACG GGTGACGGGGAATCAGGGTT CGATTCCGGAGAGGCAGCCT

S_triareu TATAAACGTCTGCCTTATCA ACTA--GATGGTAGGGTAAC GGCCTACCATGGTTGTGACG GGTGACGGGGAATCAGGGTT CGATTCCGGAGAGGCAGCCT

C_AsemiSP nnnnnnnnnnnnnnnnnnnn nnnnnnnnnnnnnnnnnnnn nnnnnnnnnnnnnnnnnnnn nnnnnnnnnnnnnnnnnnnn nnnnnnnnnnnnnnnnnnnn

C_BsemiSP TGCGAGCGTCTGCCTTATCA ACTA--GATGGTAGGGTAAC GGCCTACCATGGTTGTGACG GGTGACGGGGAATCAGGGTT CGATTCCGGAGAGGCAGCCT

A_Avampyr TATGAGCGTCTGCCTTATCA ACTA--GATGGTAGGGTAAC GGCCTACCATGGTTGTGACG GGTGACGGGGAATCAGGGTT CGATTCCGGAGAGGCAGCCT

A_Bvampyr TGTGAGCGTCTGCCTTATCA ACTA--GATGGTAGGGTAAC GGCCTACCATGGTTGTGACG GGTGACGGGGAATCAGGGTT CGATTCCGGAGAGGCAGCCT

A_Cvampyr TGTGAGCGTCTGCCTTATCA ACTA--GATGGTAGGGTAAC GGCCTACCATGGTTGTGACG GGTGACGGGGAATCAGGGTT CGATTCCGGAGAGGCAGCCT

A_Dvampyr TATGAGCGTCTGCCTTATCA ACTA--GATGGTAGGGTAAC GGCCTACCATGGTTGTGACG GGTGACGGGGAATCAGGGTT CGATTCCGGAGAGGCAGCCT

A_Ainfern TCTGAGCGTCTGCCTTATCA ACTA--GATGGTAGGGTAGC GGCCTACCATGGTTGTGACG GGTGACGGGGAATCAGGGTT CGATTCCGGAGAGGCAGCCT

A_Binfern TCTGAGCGTCTGCCTTATCA ACTA--GATGGTAGGGTAGC GGCCTACCATGGTTGTGACG GGTGACGGGGAATCAGGGTT CGATTCCGGAGAGGCAGCCT

A_Cinfern TCTGAGCGTCTGCCTTATCA ACTA--GATGGTAGGGTAGC GGCCTACCATGGTTGTGACG GGTGACGGGGAATCAGGGTT CGATTCCGGAGAGGCAGCCT

A_Dinfern TCTGAGCGTCTGCCTTATCA ACTA--GATGGTAGGGTAGC GGCCTACCATGGTTGTGACG GGTGACGGGGAATCAGGGTT CGATTCCGGAGAGGCAGCCT

A_Einfern TCTGAGCGTCTGCCTTATCA ACTA--GATGGTAGGGTAGC GGCCTACCATGGTTGTGACG GGTGACGGGGAATCAGGGTT CGATTCCGGAGAGGCAGCCT

A_Acorall TCTGAGCGTCTGCCTTATCA ACTA--GATGGTAGGGTAAC GGCCTACCATGGTTGTGACG GGTGACGGGGAATCAGGGTT CGATTCCGGAGAGGCAGCCT

A_Bcorall TCTGAGCGTCTGCCTTATCA ACTA--GATGGTAGGGTAAC GGCCTACCATGGTTGTGACG GGTGACGGGGAATCAGGGTT CGATTCCGGAGAGGCAGCCT

A_nsp1229 TCTGAGCGTCTGCCTTATCA ACTA--GATGGTAGGGTAAC GGCCTACCATGGTTGTGACG GGTGACGGGGAATCAGGGTT CGATTCCGGAGAGGCAGCCT

A_nsp1396 TCTGAGCGTCTGCCTTATCA ACTA--GATGGTAGGGTAAC GGCCTACCATGGTTGTGACG GGTGACGGGGAATCAGGGTT CGATTCCGGAGAGGCAGCCT

A_Aerythr nnnnnnnnnnnnnnnnnnnn nnnnnnnnnnnnnnnnnnnn nnnnnnnnnnnnnnnnnnnn nnnnnnnnnnnnnnnnnnnn nnnnnnnnnnnnnnnnnnnn

A_Berythr nnnnnnnnnnnnnnnnnnnn nnnnnnnnnnnnnnnnnnnn nnnnnnnnnnnnnnnnnnnn nnnnnnnnnnnnnnnnnnnn nnnnnnnnnnnnnnnnnnnn

A_AhovaSP AAAGAGCGTCTGCCTTATCA ACTA--GATGGTAGGGTAAC GGCCTACCATGGTTGTGACG GGTGACGGGGAATCAGGGTT CGATTCCGGAGAGGCAGCCT

A_BhovaSP AAAGAGCGTCTGCCTTATCA ACTA--GATGGTAGGGTAAC GGCCTACCATGGTTGTGACG GGTGACGGGGAATCAGGGTT CGATTCCGGAGAGGCAGCCT

A_ignipes AAAGAACGTCTGCCTTATCA ACTA--GATGGTAGGGTAAC GGCCTACCATGGTTGTGACG GGTGACGGGGAATCAGGGTT CGATTCCGGAGAGGCAGCCT

A_Bcowani TCTGAGCGTCTGCCTTATCA ACTA--GATGGTAGGGTAAC GGCCTACCATGGTTGTGACG GGTGACGGGGAATCAGGGTT CGATTCCGGAGAGGCAGCCT

A_Ccowani nnnnnnnnnnnnnnnnnnnn nnnnnnnnnnnnnnnnnnnn nnnnnnnnnnnnnnnnnnnn nnnnnnnnnnnnnnnnnnnn nnnnnnnnnnnnnnnnnnnn

A_Dcowani TCTGAGCGTCTGCCTTATCA ACTA--GATGGTAGGGTAAC GGCCTACCATGGTTGTGACG GGTGACGGGGAATCAGGGTT CGATTCCGGAGAGGCAGCCT

A_Asangui nnnnnnnnnnnnnnnnnnnn nnnnnnnnnnnnnnnnnnnn nnnnnnnnnnnnnnnnnnnn nnnnnnnnnnnnnnnnnnnn nnnnnnnnnnnnnnnnnnnn

A_Bsangui nnnnnnnnnnnnnnnnnnnn nnnnnnnnnnnnnnnnnnnn nnnnnnnnnnnnnnnnnnnn nnnnnnnnnnnnnnnnnnnn nnnnnnnnnnnnnnnnnnnn

A_Csangui nnnnnnnnnnnnnnnnnnnn nnnnnnnnnnnnnnnnnnnn nnnnnnnnnnnnnnnnnnnn nnnnnnnnnnnnnnnnnnnn nnnnnnnnnnnnnnnnnnnn

A_Dsangui nnnnnnnnnnnnnnnnnnnn nnnnnnnnnnnnnnnnnnnn nnnnnnnnnnnnnnnnnnnn nnnnnnnnnnnnnnnnnnnn nnnnnnnnnnnnnnnnnnnn

Doratogon GAGAAACGGCTACCACATCC AAGGAAGGCAGCAGGCACGC AAATTACCCACTCCCAGAAC GGG-GAGGTAGTGACGAAAA ATAACAATGCGGGACTCTTA

Mad_maxAS GAGAGACGGCTACCACATCC AAGGAAGGCAGCAGGCACGA AAATTACCCAATCCCCGAAC GGG-GAGGTAGTGACGAGAA ATAACGATGCGGGACTCTTT

Mad_maxBS GAGAGACGGCTACCACATCC AAGGAAGGCAGCAGGCACGA AAATTACCCAATCCCCGAAC GGG-GAGGTAGTGACGAGAA ATAACGATGCGGGACTCTTT

S_simplex GAGAGACGGCTACCACATCC AAGGAAGGCAGCAGGCACGA AAATTACCCAATCCCCGAAC GGG-GAGGTAGTGACGAGAA ATAACAATGCGGGACTCTTA

S_triareu GAGAGACGGCTACCACATCC AAGGAAGGCAGCAGGCACGA AAATTACCCAATCCCCGAAC GGGAGAGGTAGTGACGAGAA ATAACAATGCGGGACTCTTA

C_AsemiSP nnnnnnnnnnnnnnnnnnnn nnnnnnnnnnnnnnnnnnnn nnnnnnnnnnnnnnnnnnnn nnnnnnnnnnnnnnnnnnnn nnnnnnnnnnnnnnnnnnnn

C_BsemiSP GAGAGACGGCTACCACATCC AAGGAAGGCAGCAGGCACGA AAATTACCCAATCCCCGAAC GGG-GAGGTAGTGACGTGGA ATAACAATGCGGGACTCTTA

A_Avampyr GAGAGACGGCTACCACATCC AAGGAAGGCAGCAGGCACGA AAATTACCCAATCCCAGATT GGG-GAGGTAGTGACGTGAA ATAACAATGCGGGACTCTTA

A_Bvampyr GAGAGACGGCTACCACATCC AAGGAAGGCAGCAGGCACGA AAATTACCCAATCCCAGATT GGG-GAGGTAGTGACGTGAA ATAACAATGCGGGACTCTTA

A_Cvampyr GAGAGACGGCTACCACATCC AAGGAAGGCAGCAGGCACGA AAATTACCCAATCCCAGATT GGG-GAGGTAGTGACGTGAA ATAACAATGCGGGACTCTTA

A_Dvampyr GAGAGACGGCTACCACATCC AAGGAAGGCAGCAGGCACGA AAATTACCCAATCCCAGATT GGG-GAGGTAGTGACGTGAA ATAACAATGCGGGACTCTTA

A_Ainfern GAGAGACGGCTACCACATCC AAGGAAGGCAGCAGGCACGA AAATTACCCAATCCCAGATT GGG-GAGGTAGTGACGTGAA ATAACAATGCGGGACTCTTA

A_Binfern GAGAGACGGCTACCACATCC AAGGAAGGCAGCAGGCACGA AAATTACCCAATCCCAGATT GGG-GAGGTAGTGACGTGAA ATAACAATGCGGGACTCTTA

A_Cinfern GAGAGACGGCTACCACATCC AAGGAAGGCAGCAGGCACGA AAATTACCCAATCCCAGATT GGG-GAGGTAGTGACGTGAA ATAACAATGCGGGACTCTTA

A_Dinfern GAGAGACGGCTACCACATCC AAGGAAGGCAGCAGGCACGA AAATTACCCAATCCCAGATT GGG-GAGGTAGTGACGTGAA ATAACAATGCGGGACTCTTA

A_Einfern GAGAGACGGCTACCACATCC AAGGAAGGCAGCAGGCACGA AAATTACCCAATCCCAGATT GGG-GAGGTAGTGACGTGAA ATAACAATGCGGGACTCTTA

A_Acorall GAGAGACGGCTACCACATCC AAGGAAGGCAGCAGGCACGA AAATTACCCAATCCCAGATT GGG-GAGGTAGTGACGTGAA ATAACAATGCGGGACTCTTA

A_Bcorall GAGAGACGGCTACCACATCC AAGGAAGGCAGCAGGCACGA AAATTACCCAATCCCAGATT GGG-GAGGTAGTGACGTGAA ATAACAATGCGGGACTCTTA

A_nsp1229 GAGAGACGGCTACCACATCC AAGGAAGGCAGCAGGCACGA AAATTACCCAATCCCGGATT GGG-GAGGTAGTGACGTGAA ATAACAATGCGGGACTCTTA

A_nsp1396 GAGAGACGGCTACCACATCC AAGGAAGGCAGCAGGCACGA AAATTACCCAATCCCGGATT GGG-GAGGTAGTGACGTGAA ATAACAATGCGGGACTCTTA

A_Aerythr nnnnnnnnnnnnnnnnnnnn nnnnnnnnnnnnnnnnnnnn nnnnnnnnnnnnnnnnnnnn nnnnnnnnnnnnnnnnnnnn nnnnnnnnnnnnnnnnnnnn

A_Berythr nnnnnnnnnnnnnnnnnnnn nnnnnnnnnnnnnnnnnnnn nnnnnnnnnnnnnnnnnnnn nnnnnnnnnnnnnnnnnnnn nnnnnnnnnnnnnnnnnnnn

A_AhovaSP GAGAGACGGCTACCACATCC AAGGAAGGCAGCAGGCACGA AAATTACCCAATCCCGGATT GGG-GAGGTAGTGACGTGAA ATAACAATGCGGGACTCTTA

A_BhovaSP GAGAGACGGCTACCACATCC AAGGAAGGCAGCAGGCACGA AAATTACCCAATCCCGGATT GGG-GAGGTAGTGACGTGAA ATAACAATGCGGGACTCTTA

A_ignipes GAGAGACGGCTACCACATCC AAGGAAGGCAGCAGGCACGA AAATTACCCAATCCCGGATT GGG-GAGGTAGTGACGTGAA ATAACAATGCGGGACTCTTA

A_Bcowani GAGAGACGGCTACCACATCC AAGGAAGGCAGCAGGCACGA AAATTACCCAATCCCAGATT GGG-GAGGTAGTGACGTGAA ATAACAATGCGGGACTCTTA

A_Ccowani nnnnnnnnnnnnnnnnnnnn nnnnnnnnnnnnnnnnnnnn nnnnnnnnnnnnnnnnnnnn nnnnnnnnnnnnnnnnnnnn nnnnnnnnnnnnnnnnnnnn

A_Dcowani GAGAGACGGCTACCACATCC AAGGAAGGCAGCAGGCACGA AAATTACCCAATCCCAGATT GGG-GAGGTAGTGACGTGAA ATAACAATGCGGGACTCTTA

A_Asangui nnnnnnnnnnnnnnnnnnnn nnnnnnnnnnnnnnnnnnnn nnnnnnnnnnnnnnnnnnnn nnnnnnnnnnnnnnnnnnnn nnnnnnnnnnnnnnnnnnnn

A_Bsangui nnnnnnnnnnnnnnnnnnnn nnnnnnnnnnnnnnnnnnnn nnnnnnnnnnnnnnnnnnnn nnnnnnnnnnnnnnnnnnnn nnnnnnnnnnnnnnnnnnnn

A_Csangui nnnnnnnnnnnnnnnnnnnn nnnnnnnnnnnnnnnnnnnn nnnnnnnnnnnnnnnnnnnn nnnnnnnnnnnnnnnnnnnn nnnnnnnnnnnnnnnnnnnn

A_Dsangui nnnnnnnnnnnnnnnnnnnn nnnnnnnnnnnnnnnnnnnn nnnnnnnnnnnnnnnnnnnn nnnnnnnnnnnnnnnnnnnn nnnnnnnnnnnnnnnnnnnn

Doratogon CGAGGCCCCGTAATTGGAAT GAGTCCACTTTAAATCCTTT AA-CGAGGACCTATTGGAGG GCAAGTCTGGTGCCAGCAGC CGCGGTAATTCCAGCTCCAA

Mad_maxAS CGAGGCCCCGTAATTGGAAT GAGAGGAGTTTACAATACTC GCTCGAGAAACTATTGGAGG GCAAGTCTGGTGCCAGCAGC CGCGGTAATTCCAGCTCCAA

Mad_maxBS CGAGGCCCCGTAATTGGAAT GAGAGGAGTTTACAATACTC GCTCGAGAAACTATTGGAGG GCAAGTCTGGTGCCAGCAGC CGCGGTAATTCCAGCTCCAA

S_simplex CGAGGCCCCGTAATTGGAAT GAGAGGAGTTTAAAACTCTT ACTCGAGAAACTATTGGAGG GCAAGTCTGGTGCCAGCAGC CGCGGTAATTCCAGCTCCAA

S_triareu CGAGGCCCCGTAATTGGAAT GAGAGGAG-TCAAAACTCTT ACTCGAGTAACTATTGGAGG GCAAGTGTGGTGCCAGCAGC CGCGGTAATTCCAGCTCCAA

C_AsemiSP nnnnnnnnnnnnnnnnnnnn nnnnnnnnnnnnnnnnnnnn nnnnnnnnnnnnnnnnnnnn nnnnnnnnnnnnnnnnnnnn nnnnnnnnnnnnnnnnnnnn

C_BsemiSP CGAGGCCCCGTAATTGGAAT GAGTGGAGTTCAAAACTCTC ACTCGAGAAACTATTGGAGG GCAAGTCTGGTGCCAGCAGC CGCGGTAATTCCAGCTCCAA

A_Avampyr CGAGGCCCCGTAATTGGAAT GAGTGGAGTTTAAAACTCTC ACTCTAGAAACTATTGGAGG GCAAGTCTGGTGCCAGCAGC CGCGGTAATTCCAGCTCCAA

A_Bvampyr CGAGGCCCCGTAATTGGAAT GAGTGGAGTTTAAAACTCTC ACTCTAGAAACTATTGGAGG GCAAGTCTGGTGCCAGCAGC CGCGGTAATTCCAGCTCCAA

A_Cvampyr CGAGGCCCCGTAATTGGAAT GAGTGGAGTTTAAAACTCTC ACTCTAGAAACTATTGGAGG GCAAGTCTGGTGCCAGCAGC CGCGGTAATTCCAGCTCCAA

A_Dvampyr CGAGGCCCCGTAATTGGAAT GAGTGGAGTTTAAAACTCTC ACTCTAGAAACTATTGGAGG GCAAGTCTGGTGCCAGCAGC CGCGGTAATTCCAGCTCCAA

A_Ainfern CGAGGCCCCGTAATTGGAAT GAGTGGAGTTTAAAACTCTC ACTCTAGAAACTATTGGAGG GCAAGTCTGGTGCCAGCAGC CGCGGTAATTCCAGCTCCAA

A_Binfern CGAGGCCCCGTAATTGGAAT GAGTGGAGTTTAAAACTCTC ACTCTAGAAACTATTGGAGG GCAAGTCTGGTGCCAGCAGC CGCGGTAATTCCAGCTCCAA

A_Cinfern CGAGGCCCCGTAATTGGAAT GAGTGGAGTTTAAAACTCTC ACTCTAGAAACTATTGGAGG GCAAGTCTGGTGCCAGCAGC CGCGGTAATTCCAGCTCCAA

A_Dinfern CGAGGCCCCGTAATTGGAAT GAGTGGAGTTTAAAACTCTC ACTCTAGAAACTATTGGAGG GCAAGTCTGGTGCCAGCAGC CGCGGTAATTCCAGCTCCAA

A_Einfern CGAGGCCCCGTAATTGGAAT GAGTGGAGTTTAAAAATCTC ACTCTAGAAACTATTGGAGG GCAAGTCTGGTGCCAGCAGC CGCGGTAATTCCAGCTCCAA

A_Acorall CGAGGCCCCGTAATTGGAAT GAGTGGAGTTTAAAACTCTC ACTCAAGAAACTATTGGAGG GCAAGTCTGGTGCCAGCAGC CGCGGTAATTCCAGCTCCAA

A_Bcorall CGAGGCCCCGTAATTGGAAT GAGTGGAGTTTAAAACTCTC ACTCAAGAAACTATTGGAGG GCAAGTCTGGTGCCAGCAGC CGCGGTAATTCCAGCTCCAA

A_nsp1229 CGAGGCCCCGTAATTGGAAT GAGTGGAGTTCAAAACTCTC ACTCTAGAAACTATTGGAGG GCAAGTCTGGTGCCAGCAGC CGCGGTAATTCCAGCTCCAA

A_nsp1396 CGAGGCCCCGTAATTGGAAT GAGTGGAGTTTAAAACTCTC ACTCTAGAAACTATTGGAGG GCAAGTCTGGTGCCAGCAGC CGCGGTAATTCCAGCTCCAA

A_Aerythr nnnnnnnnnnnnnnnnnnnn nnnnnnnnnnnnnnnnnnnn nnnnnnnnnnnnnnnnnnnn nnnnnnnnnnnnnnnnnnnn nnnnnnnnnnnnnnnnnnnn

A_Berythr nnnnnnnnnnnnnnnnnnnn nnnnnnnnnnnnnnnnnnnn nnnnnnnnnnnnnnnnnnnn nnnnnnnnnnnnnnnnnnnn nnnnnnnnnnnnnnnnnnnn

A_AhovaSP CGAGGCCCCGTAATTGGAAT GAGTGGAGTTTAAAACTCTC ACTCAAGAAACTATTGGAGG GCAAGTCTGGTGCCAGCAGC CGCGGTAATTCCAGCTCCAA

A_BhovaSP CGAGGCCCCGTAATTGGAAT GAGTGGAGTTTAAAACTCTC ACTCAAGAAACTATTGGAGG GCAAGTCTGGTGCCAGCAGC CGCGGTAATTCCAGCTCCAA

A_ignipes CGAGGCCCCGTAATTGGAAT GAGTGGAGTTTAAAACTCTC ACTCAAGAAACTATTGGAGG GCAAGTCTGGTGCCAGCAGC CGCGGTAATTCCAGCTCCAA

A_Bcowani CGAGGCCCCGTAATTGGAAT GAGTGGAGTTTAAAACTCTC ACTCTAGAAACTATTGGAGG GCAAGTCTGGTGCCAGCAGC CGCGGTAATTCCAGCTCCAA

A_Ccowani nnnnnnnnnnnnnnnnnnnn nnnnnnnnnnnnnnnnnnnn nnnnnnnnnnnnnnnnnnnn nnnnnnnnnnnnnnnnnnnn nnnnnnnnnnnnnnnnnnnn

A_Dcowani CGAGGCCCCGTAATTGGAAT GAGTGGAGTTTAAAACTCTC ACTCTAGAAACTATTGGAGG GCAAGTCTGGTGCCAGCAGC CGCGGTAATTCCAGCTCCAA

A_Asangui nnnnnnnnnnnnnnnnnnnn nnnnnnnnnnnnnnnnnnnn nnnnnnnnnnnnnnnnnnnn nnnnnnnnnnnnnnnnnnnn nnnnnnnnnnnnnnnnnnnn

A_Bsangui nnnnnnnnnnnnnnnnnnnn nnnnnnnnnnnnnnnnnnnn nnnnnnnnnnnnnnnnnnnn nnnnnnnnnnnnnnnnnnnn nnnnnnnnnnnnnnnnnnnn

A_Csangui nnnnnnnnnnnnnnnnnnnn nnnnnnnnnnnnnnnnnnnn nnnnnnnnnnnnnnnnnnnn nnnnnnnnnnnnnnnnnnnn nnnnnnnnnnnnnnnnnnnn

A_Dsangui nnnnnnnnnnnnnnnnnnnn nnnnnnnnnnnnnnnnnnnn nnnnnnnnnnnnnnnnnnnn nnnnnnnnnnnnnnnnnnnn nnnnnnnnnnnnnnnnnnnn

Doratogon TAGCGTATATTAAAGTTGTT GCGGTTAAAAAGCTCGTAGT TGGATGTC--GTTGCGGGCG GGTGGTCC--ACCGTCTCGG TGGCTTA--CTACCCG---T

Mad_maxAS TGGCGTATATTAAAGCTGCT GCGGTTAAAAAGCTCGTAGT TGGATGTC-AGTCTCGGGCG AGCGGTCC--ACCCTCCGCG GTGGCGA--CTGCACG---C

Mad_maxBS TGGCGTATATTAAAGCTGCT GCGGTTAAAAAGCTCGTAGT TGGATGTC-AGTCTCGGGCG AGCGGTCC--ACCCTCCGCG GTGGCGA--CTGCACG---C

S_simplex TAGCATATGTTAAAGCTGCT GCGGTTAAAAAGCTCGTAGT TGGATGTC-AGTCTCGGTCG TGCGGTCC--ACCTTAAGGG GTGGTGA--CTGCTCG---T

S_triareu TAGCATATGTTAAAGCTGTT GCGGTTAAAAAGCTCGTAGT TGGATGTC-AGTCTCGGTCG TGCGGTCC--ACCTTAAGGG GTGGTGA--CTGCTCG---T

C_AsemiSP nnnnnnnnnnnnnnnnnnnn nnnnnnnnnnnnnnnnnnnn nnnnnnnnnnnnnnnnnnnn nnnnnnnnnnnnnnnnnnnn nnnnnnnnnnnnnnnnnnnn

C_BsemiSP TAGCGTATACTAAAGCTGCT GCGGTTAAAAAGCTCGTAGT TGGATGTC-AGTTCGGTCCG GGAGGTCCCACCCCTCCGGG GTGGAGACCCTACCCGAGAC

A_Avampyr TAGCATATACTAAAGCTGCT GCGGTTAAAAAGCTCGTAGT TGGATGTC-AGTTTCGTGCT GGCGGTCCC-CCCCTCCGGG GCGGCGA--CTGCTTG---T

A_Bvampyr TAGCATATACTAAAGCTGCT GCGGTTAAAAAGCTCGTAGT TGGATGTC-AGTTTCGTGCT GGCGGTCCC-CCCCTCCGGG GCGGCGA--CTGCTTG---T

A_Cvampyr TAGCATATACTAAAGCTGCT GCGGTTAAAAAGCTCGTAGT TGGATGTC-AGTTTCGTGCT GGCGGTCCC-CCCCTCCGGG GCGGCGA--CTGCTTG---T

A_Dvampyr TAGCATATACTAAAGCTGCT GCGGTTAAAAAGCTCGTAGT TGGATGTC-AGTTTCGTGCT GGCGGTCCC-CCCCTCCGGG GCGGCGA--CTGCTTG---T

A_Ainfern TAGCATATACTAAAGCTGCT GCGGTTAAAAAGCTCGTAGT TGGATGTC-AGTTTCGTGCT GGCGGTCCC-CCCCTCCGGG GCGGCGA--CTGCTTG---T

A_Binfern TAGCATATACTAAAGCTGCT GCGGTTAAAAAGCTCGTAGT TGGATGTC-AGTTTCGTGCT GGCGGTCCC-CCCCTCCGGG GCGGCGA--CTGCTTG---T

A_Cinfern TAGCATATACTAAAGCTGCT GCGGTTAAAAAGCTCGTAGT TGGATGTC-AGTTTCGTGCT GGCGGTCCC-CCCCTCCGGG GCGGCGA--CTGCTTG---T

A_Dinfern TAGCATATACTAAAGCTGCT GCGGTTAAAAAGCTCGTAGT TGGATGTC-AGTTTCGTGCT GGCGGTCCC-CCCCTCCGGG GCGGCGA--CTGCTTG---T

A_Einfern TAGCATATACTAAAGCTGCT GCGGTTAAAAAGCTCGTAGT TGGATGTC-AGTTTCGTGCT GGCGGTCCC-CCCCTCCGGG GCGGCGA--CTGCTTG---T

A_Acorall TAGCGTATACTAAAGCTGCT GCGGTTAAAAAGCTCGTAGT TGGATGTC-AGTTTCGTGCT GGCGGTCCC-CCCCTCCGGG GCGGCGA--CTGCTTG---T

A_Bcorall TAGCGTATACTAAAGCTGCT GCGGTTAAAAAGCTCGTAGT TGGATGTC-AGTTTCGTGCT GGCGGTCCC-CCCCTCCGGG GCGGCGA--CTGCTTG---T

A_nsp1229 TAGCATATACTAAAGCTGCT GCGGTTAAAAAGCTCGTAGT TGGATGTC-AGTCTCGTGCT TGCGGTCCC-CCCCTCCGGG GCGGCGA--CTGCTTG---T

A_nsp1396 TAGCATATACTAAAGCTGCT GCGGTTAAAAAGCTCGTAGT TGGACGTC-AGTCTCGTGCG TGCGGTCCC-CCCCTCCGGG GCGGCGA--CTGCTCG---T

A_Aerythr nnnnnnnnnnnnnnnnnnnn nnnnnnnnnnnnnnnnnnnn nnnnnnnnnnnnnnnnnnnn nnnnnnnnnnnnnnnnnnnn nnnnnnnnnnnnnnnnnnnn

A_Berythr nnnnnnnnnnnnnnnnnnnn nnnnnnnnnnnnnnnnnnnn nnnnnnnnnnnnnnnnnnnn nnnnnnnnnnnnnnnnnnnn nnnnnnnnnnnnnnnnnnnn

A_AhovaSP TAGCATATACTAAAGCTGCT GCGGTTAAAAAGCTCGTAGT TGGAAATCAAGTTTCGAGTC GGCGGTCC--CCCCTCCGGG GCGGCGA--CTGCCGG---T

A_BhovaSP TAGCATATACTAAAGCTGCT GCGGTTAAAAAGCTCGTAGT TGGAAATCAAGTTTCGAGTC GGCGGTCC--CCCCTCCGGG GCGGCGA--CTGCCGG---T

A_ignipes TAGCATATACTAAAGCTGCT GCGGTTAAAAAGCTCGTAGT TGGAAATC-AAGTTCGAGTC GGCGGTCC--CCCCTCCGGG GCGGCGA--CTGCCGG--TC

A_Bcowani TAGCGTATACTAAACTTGCT GTGGTTAAAAAGCTCGTAGT TGGATGTC-AGTTTCGTGCT GGCGGTCCC-CCCCTCCGGG GTGGCGA--CTGCCGG---T

A_Ccowani nnnnnnnnnnnnnnnnnnnn nnnnnnnnnnnnnnnnnnnn nnnnnnnnnnnnnnnnnnnn nnnnnnnnnnnnnnnnnnnn nnnnnnnnnnnnnnnnnnnn

A_Dcowani TAGCGTATACTAAACTTGCT GTGGTTAAAAAGCTCGTAGT TGGATGTC-AGTTTCGTGCT GGCGGTCCC-CCCCTCCGGG GTGGCGA--CTGCCGG---T

A_Asangui nnnnnnnnnnnnnnnnnnnn nnnnnnnnnnnnnnnnnnnn nnnnnnnnnnnnnnnnnnnn nnnnnnnnnnnnnnnnnnnn nnnnnnnnnnnnnnnnnnnn

A_Bsangui nnnnnnnnnnnnnnnnnnnn nnnnnnnnnnnnnnnnnnnn nnnnnnnnnnnnnnnnnnnn nnnnnnnnnnnnnnnnnnnn nnnnnnnnnnnnnnnnnnnn

A_Csangui nnnnnnnnnnnnnnnnnnnn nnnnnnnnnnnnnnnnnnnn nnnnnnnnnnnnnnnnnnnn nnnnnnnnnnnnnnnnnnnn nnnnnnnnnnnnnnnnnnnn

A_Dsangui nnnnnnnnnnnnnnnnnnnn nnnnnnnnnnnnnnnnnnnn nnnnnnnnnnnnnnnnnnnn nnnnnnnnnnnnnnnnnnnn nnnnnnnnnnnnnnnnnnnn

Doratogon CCCGCTACCTACCA-TCCGG CCCCTCCCGCTATGCTCTTC ACCGGGT--GTGGCGGG--T GGCCGGAACGTTTACTTTGA AAAAATTAGAGTGCTCAAAG

Mad_maxAS ATCGGACTGCGTGG---TGG GCCTGCCCTCGGTGCTCTTA ACCGGGT--GCCGCTGGGT- GGCCCCTGGCCGTACTGTGA GAAAATCAGGGTGCTCAAAG

Mad_maxBS ATCGGACTGCGTGG---TGG GCCTGCCCTCGGTGCTCTTA ACCGGGT--GCCGCTGGGT- GGCCCCTGGCCGTACTGTGA GAAAATCAGGGTGCTCAAAG

S_simplex TTCGGACTGAGTTG--CCGG --CTGCCCTCGATGCTCTTG ACCGGGT--GTCGTTGGGCG TGCTGGAGACTGTACTTTGA AAAAATCAAAGTGATCAAAA

S_triareu TTCGGACTGAGTTG--CCGG --CTGCCCTCGATGCTCTTG ACCGGGT--GTCGTTGGGCA TGCTGGAGACTGTACTTTGA AAAAATCAAAGTGATCAAAA

C_AsemiSP nnnnnnnnnnnnnnnnnnnn nnnnnnnnnnnnnnnnnnnn nnnnnnnnnnnnnnnnnnnn nnnnnnnnnnnnnnnnnnnn nnnnnnnnnnnnnnnnnnnn

C_BsemiSP TCCGGACTCGGTTAGTCCGG --CGGCCCTCCGTGCCCTTG ACCGGGTGAGGAGCGTTGCC TGCCGGAGACCGTACTTTGA AAAAATCAAAGTGATCAAAA

A_Avampyr CTCGAACTCAGTTG--CCGG --CTGCCCTCGATGCTCTTG ACCGGGT--GTCGCTGGGCG TGCCGGAGACTGTACTTTGA AAAAATCAAAGTGATCAAAA

A_Bvampyr CTCGAACTCAGTTG--CCGG --CTGCCCTCGATGCTCTTG ACCGGGT--GTCGCTGGGCG TGCCGGAGACTGTACTTTGA AAAAATCAAAGTGATCAAAA

A_Cvampyr CTCGAACTCAGTTG--CCGG --CTGCCCTCGATGCTCTTG ACCGGGT--GTCGCTGGGCG TGCCGGAGACTGTACTTTGA AAAAATCAAAGTGATCAAAA

A_Dvampyr CTCGAACTCAGTTG--CCGG --CTGCCCTCGATGCTCTTG ACCGGGT--GTCGCTGGGCG TGCCGGAGACTGTACTTTGA AAAAATCAAAGTGATCAAAA

A_Ainfern CTCGAACTCAGTTG--CCGG --CTGCCCTCGATGCTCTTG ACCGGGT--GTCGCTGGGCG TGCCGGAGACTGTACTTTGA AAAAATCAAAGTGATCAAAA

A_Binfern CTCGAACTCAGTTG--CCGG --CTGCCCTCGATGCTCTTG ACCGGGT--GTCGCTGGGCG TGCCGGAGACTGTACTTTGA AAAAATCAAAGTGATCAAAA

A_Cinfern CTCGAACTCAGTTG--CCGG --CTGCCCTCGATGCTCTTG ACCGGGT--GTCGCTGGGCG TGCCGGAGACTGTACTTTGA AAAAATCAAAGTGATCAAAA

A_Dinfern CTCGAACTCAGTTG--CCGG --CTGCCCTCGATGCTCTTG ACCGGGT--GTCGCTGGGCG TGCCGGAGACTGTACTTTGA AAAAATCAAAGTGATCAAAA

A_Einfern CTCGAACTCAGTTG--CCGG --CTGCCCTCGATGCTCTTG ACCGGGT--GTCGCTGGGCG TGCCGGAGACTGTACTTTGA AAAAATCAAAGTGATCAAAA

A_Acorall CTCGAACTCAGTTG--CCGG --CTGCCCTCGATGCTCTTG ACCGGGT--GTCGCTGGGCG TGCCGGAGACTGTACTTTGA AAAAATCAAAGTGATCAAAA

A_Bcorall CTCGAACTCAGTTG--CCGG --CTGCCCTCGATGCTCTTG ACCGGGT--GTCGCTGGGCG TGCCGGAGACTGTACTTTGA AAAAATCAAAGTGATCAAAA

A_nsp1229 CTCGAACTCAGTTG--CCGG --CTGCCCTCGATGCTCTTG ACCGGGT--GTCGCTGGGCG TGCCGGAGACTGTACTTTGA AAAAATCAAAGTGATCAAAA

A_nsp1396 CTCGGACTCGTTTG--CCGG --CTGCCCTCGATGCTCTTG ACCGGGT--GTCGCTGGGCG TGCCGGAGACTGTACTTTGA AAAAATCAAAGTGATCAAAA

A_Aerythr nnnnnnnnnnnnnnnnnnnn nnnnnnnnnnnnnnnnnnnn nnnnnnnnnnnnnnnnnnnn nnnnnnnnnnnnnnnnnnnn nnnnnnnnnnnnnnnnnnnn

A_Berythr nnnnnnnnnnnnnnnnnnnn nnnnnnnnnnnnnnnnnnnn nnnnnnnnnnnnnnnnnnnn nnnnnnnnnnnnnnnnnnnn nnnnnnnnnnnnnnnnnnnn

A_AhovaSP CTCGAGCTCAGTTG--CCGG --CTGCCCTCGATGCTCTTT GCCGGGT--GTCGCTGGGCG TGCCGGAGACTGTACTTTGA AAAAATCAAAGTGATCAAAA

A_BhovaSP CTCGAGCTCAGTTG--CCGG --CTGCCCTCGATGCTCTTT GCCGGGT--GTCGCTGGGCG TGCCGGAGACTGTACTTTGA AAAAATCAAAGTGATCAAAA

A_ignipes CTCGAGCTCAGTTG--CCGG --CTGCCCTCGATGCTCTTC GCCGGGT--GTCGCTGGGCG TGCCGGAGACTGTACTTTGA AAAAATCAAAGTGATCAAAA

A_Bcowani TTCGAACTCAGTTG--CCGG --CTGCCCTCGATGCTCTTG ACCGGGT--GTCGCTGGGCG TGCCGGAGACTGTACTTTGA AAAAATCAAAGTGATCAAAA

A_Ccowani nnnnnnnnnnnnnnnnnnnn nnnnnnnnnnnnnnnnnnnn nnnnnnnnnnnnnnnnnnnn nnnnnnnnnnnnnnnnnnnn nnnnnnnnnnnnnnnnnnnn

A_Dcowani TTCGAACTCAGTTG--CCGG --CTGCCCTCGATGCTCTTG ACCGGGT--GTCGCTGGGCG TGCCGGAGACTGTACTTTGA AAAAATCAAAGTGATCAAAA

A_Asangui nnnnnnnnnnnnnnnnnnnn nnnnnnnnnnnnnnnnnnnn nnnnnnnnnnnnnnnnnnnn nnnnnnnnnnnnnnnnnnnn nnnnnnnnnnnnnnnnnnnn

A_Bsangui nnnnnnnnnnnnnnnnnnnn nnnnnnnnnnnnnnnnnnnn nnnnnnnnnnnnnnnnnnnn nnnnnnnnnnnnnnnnnnnn nnnnnnnnnnnnnnnnnnnn

A_Csangui nnnnnnnnnnnnnnnnnnnn nnnnnnnnnnnnnnnnnnnn nnnnnnnnnnnnnnnnnnnn nnnnnnnnnnnnnnnnnnnn nnnnnnnnnnnnnnnnnnnn

A_Dsangui nnnnnnnnnnnnnnnnnnnn nnnnnnnnnnnnnnnnnnnn nnnnnnnnnnnnnnnnnnnn nnnnnnnnnnnnnnnnnnnn nnnnnnnnnnnnnnnnnnnn

Doratogon CAGGCATGTCTTGCC--TGA ATATCTCAGCATGGAATGAT GGAACAGGACCTCGGTT-CT ATTTTGTCGGTTTTCGGAAG TCCGAGGTAATGATTAATAG

Mad_maxAS CAGGCGATA-AGGCCAATGA ATATGTAAGCATGGAATATG AGAACACGATCTCGGATCCT GTTTTATTGGTTTCTGGAA- -CCGAGGTAATGATTAATTG

Mad_maxBS CAGGCGATA-AGGCCAATGA ATATGTAAGCATGGAATATG AGAACACGATCTCGGATCCT GTTTTATTGGTTTCTGGAA- -CCGAGGTAATGATTAATTG

S_simplex CAGGTCT---GTGGCCTTGA ATATCTGAGCATGGAATATG AAAATAAGACCTCGAGT-CT ATTTTGTTGGTTTCTGGAA- -CCGAGGTAATGATTAATTG

S_triareu CAGGTCT---GTGGCCATGA ATATCTGAGCATGGAATATG AGAATAAGACCTCGAGT-CT ATTTTGATGTTTTCTGGAA- -CCGAGGTAATGATTAATTG

C_AsemiSP nnnnnnnnnnnnnnnnnnnn nnnnnnnnnnnnnnnnnnnn nnnnnnnnnnnnnnnnnnnn nnnnnnnnnnnnnnnnnnnn nnnnnnnnnnnnnnnnnnnn

C_BsemiSP CGGGCCTTACAGGGCCTCGA ATGTCTGAGCATGGAATATG GAAAGACGACCTCGAGT-CT ATTTTGTTGGTTTCTGGAA- -ACGAGGTAATGATTAATAG

A_Avampyr CAGGTCT---GTGGCCGTGA ATGTCTGAGCATGGAATATG AAAAGATGACCTCGAGT-CT ATTTTGATGGTTTCTTGAA- -CCGAGGTAATGATTAATAG

A_Bvampyr CAGGTCT---GTGGCCGTGA ATGTCTGAGCATGGAATATG AAAAGATGACCTCGAGT-CT ATTTTGATGGTTTCTTGAA- -CCGAGGTAATGATTAATAG

A_Cvampyr CAGGTCT---GTGGCCGTGA ATGTCTGAGCATGGAATATG AAAAGATGACCTCGAGT-CT ATTTTGATGGTTTCTTGAA- -CCGAGGTAATGATTAATAG

A_Dvampyr CAGGTCT---GTGGCCGTGA ATGTCTGAGCATGGAATATG AAAAGATGACCTCGAGT-CT ATTTTGATGGTTTCTTGAA- -CCGAGGTAATGATTAATAG

A_Ainfern CAGGTCT---GTGGCCGTGA ATGTCTGAGCATGGAATATG AAAAGATGACCTCGAGT-CT ATTTTGATGGTTTCTTGAA- -CCGAGGTAATGATTAATAG

A_Binfern CAGGTCT---GTGGCCGTGA ATGTCTGAGCATGGAATATG AAAAGATGACCTCGAGT-CT ATTTTGATGGTTTCTTGAA- -CCGAGGTAATGATTAATAG

A_Cinfern CAGGTCT---GTGGCCGTGA ATGTCTGAGCATGGAATATG AAAAGATGACCTCGAGT-CT ATTTTGATGGTTTCTTGAA- -CCGAGGTAATGATTAATAG

A_Dinfern CAGGTCT---GTGGCCGTGA ATGTCTGAGCATGGAATATG AAAAGATGACCTCGAGT-CT ATTTTGATGGTTTCTTGAA- -CCGAGGTAATGATTAATAG

A_Einfern CAGGTCT---GTGGCCGTGA ATGTCTGAGCATGGAATATG AAAAGATGACCTCGAGT-CT ATTTTGATGGTTTCTTGAA- -CCGAGGTAATGATTAATAG

A_Acorall CAGGTCT---GTGGCCGTGA ATGTCTGAGCATGGAATATG AAAAGATGACCTCGAGT-CT ATTTTGATGGTTTCTTGAA- -CCGAGGTAATGATTAATAG

A_Bcorall CAGGTCT---GTGGCCGTGA ATGTCTGAGCATGGAATATG AAAAGATGACCTCGAGT-CT ATTTTGATGGTTTCTTGAA- -CCGAGGTAATGATTAATAG

A_nsp1229 CAGGTCT---GTGGCCGTGA ATGCCTGAGCATGGAATATG AAAAGATGACCTCGAGT-CT ATTTTGTTGGTTTCTTGAA- -CCGAGGTAATGATTAATAG

A_nsp1396 CAGGTCT---GTGGCCGTGA ATGTCTGAGCATGGAATATG AAAAGATGACCTCGAGT-CT ATTTTGTTGGTTTCTTGAA- -CCGAGGTAATGATTAATAG

A_Aerythr nnnnnnnnnnnnnnnnnnnn nnnnnnnnnnnnnnnnnnnn nnnnnnnnnnnnnnnnnnnn nnnnnnnnnnnnnnnnnnnn nnnnnnnnnnnnnnnnnnnn

A_Berythr nnnnnnnnnnnnnnnnnnnn nnnnnnnnnnnnnnnnnnnn nnnnnnnnnnnnnnnnnnnn nnnnnnnnnnnnnnnnnnnn nnnnnnnnnnnnnnnnnnnn

A_AhovaSP CAGGTCT---GTGGCCCTGA ATGTCTGAGCATGGAATATG AAAAGATGACCTCGAGT-CA TTTTTATTGGTTTCTTGGA- -ACGAGGTAATGATTAATAG

A_BhovaSP CAGGTCT---GTGGCCCTGA ATGTCTGAGCATGGAATATG AAAAGATGACCTCGAGT-CA TTTTTATTGGTTTCTTGGA- -ACGAGGTAATGATTAATAG

A_ignipes CAGGTCT---GTGGCCCTGA ATGTCTGAGCATGGAATATG AAAAGATGACCTCGAGT-CA TTTTTATTGGTTTCTTGGA- -ACGAGGTAATGATTAATAG

A_Bcowani CAGGTCT---GTGGCCGTGA ATGTCTGAGCATGGAATATG AAAAGACGACCTCGAGT-CG ATTTTGTTGGTTTCTTGAA- -CCGAGGTAATGATTAATAG

A_Ccowani nnnnnnnnnnnnnnnnnnnn nnnnnnnnnnnnnnnnnnnn nnnnnnnnnnnnnnnnnnnn nnnnnnnnnnnnnnnnnnnn nnnnnnnnnnnnnnnnnnnn

A_Dcowani CAGGTCT---GTGGCCGTGA ATGTCTGAGCATGGAATATG AAAAGACGACCTCGAGT-CG ATTTTGTTGGTTTCTTGAA- -CCGAGGTAATGATTAATAG

A_Asangui nnnnnnnnnnnnnnnnnnnn nnnnnnnnnnnnnnnnnnnn nnnnnnnnnnnnnnnnnnnn nnnnnnnnnnnnnnnnnnnn nnnnnnnnnnnnnnnnnnnn

A_Bsangui nnnnnnnnnnnnnnnnnnnn nnnnnnnnnnnnnnnnnnnn nnnnnnnnnnnnnnnnnnnn nnnnnnnnnnnnnnnnnnnn nnnnnnnnnnnnnnnnnnnn

A_Csangui nnnnnnnnnnnnnnnnnnnn nnnnnnnnnnnnnnnnnnnn nnnnnnnnnnnnnnnnnnnn nnnnnnnnnnnnnnnnnnnn nnnnnnnnnnnnnnnnnnnn

A_Dsangui nnnnnnnnnnnnnnnnnnnn nnnnnnnnnnnnnnnnnnnn nnnnnnnnnnnnnnnnnnnn nnnnnnnnnnnnnnnnnnnn nnnnnnnnnnnnnnnnnnnn

Doratogon GGACGGACGGGGGCATTCGT ATTGCGGCGCTAGAGGTGAA ATTCTTGGACCGTCGCAAGA CGAACTAAAGCGAAAGCATT TGCCAAGAATGTTTTCATTA

Mad_maxAS GGACTGCCGGGGGCATACGT ATTGCGAGGCGAGAGGTGAA ATTCTTGGACCCTTGCAAGA CGACCAAGAGCGAAGGCATT TGCCAAGAATGTCTTCATTA

Mad_maxBS GGACTGCCGGGGGCATACGT ATTGCGAGGCGAGAGGTGAA ATTCTTGGACCCTTGCAAGA CGACCAAGAGCGAAGGCATT TGCCAAGAATGTCTTCATTA

S_simplex GGACTGCCGGGGGCATACGT ATTGCGAGGCGAGAGGTGAA ATTCTTGGACCCCCGCAAGA CGACCTAAAGCGAAAGCATT TGCCAAGAATGTTTTCATTA

S_triareu GNACTGCCGGGGGCATACCT ATTGCGAGGCGAGAGGTGAA ATTCTTGGACCCCCGCAAGA CGACCTAAAGCGAAAGCATT TGCCAAGAATGTTTTCATTA

C_AsemiSP nnnnnnnnnnnnnnnnnnnn nnnnnnnnnnnnnnnnnnnn nnnnnnnnnnnnnnnnnnnn nnnnnnnnnnnnnnnnnnnn nnnnnnnnnnnnnnnnnnnn

C_BsemiSP GGACTGCCGGGGGCATACGT ATTGCAGGGCGAGAGGTGAA ATTCATGGACCCTTGCAAGA CGACCAAGAGCGAAAGCATT TGCCAAGAATGTTTTCATTA

A_Avampyr GGCCTGCCGGGGGCATACGT ATTGCGAGGCGAGAGGTGAA ATTCTTGGACCCTTGCAAGA CGACCAAGAGCGAAAGCATT TGCCAAGAATGGTTTCGTTA

A_Bvampyr GGCCTGCCGGGGGCATACGT ATTGCGAGGCGAGAGGTGAA ATTCTTGGACCCTTGCAAGA CGACCAAGAGCGAAAGCATT TGCCAAGAATGGTTTCGTTA

A_Cvampyr GGCCTGCCGGGGGCATACGT ATTGCGAGGCGAGAGGTGAA ATTCTTGGACCCTTGCAAGA CGACCAAGAGCGAAAGCATT TGCCAAGAATGGTTTCGTTA

A_Dvampyr GGCCTGCCGGGGGCATACGT ATTGCGAGGCGAGAGGTGAA ATTCTTGGACCCTTGCAAGA CGACCAAGAGCGAAAGCATT TGCCAAGAATGGTTTCGTTA

A_Ainfern GGCCTGCCGGGGGCATACGT ATTGCGAGGCGAGAGGTGAA ATTCTTGGACCCTTGCAAGA CGACCAAGAGCGAAAGCATT TGCCAAGAATGGTTTCGTTA

A_Binfern GGCCTGCCGGGGGCATACGT ATTGCGAGGCGAGAGGTGAA ATTCTTGGACCCTTGCAAGA CGACCAAGAGCGAAAGCATT TGCCAAGAATGGTTTCGTTA

A_Cinfern GGCCTGCCGGGGGCATACGT ATTGCGAGGCGAGAGGTGAA ATTCTTGGACCCTTGCAAGA CGACCAAGAGCGAAAGCATT TGCCAAGAATGGTTTCGTTA

A_Dinfern GGCCTGCCGGGGGCATACGT ATTGCGAGGCGAGAGGTGAA ATTCTTGGACCCTTGCAAGA CGACCAAGAGCGAAAGCATT TGCCAAGAATGGTTTCGTTA

A_Einfern GGCCTGCCGGGGGCATACGT ATTGCGAGGCGAGAGGTGAA ATTCTTGGACCCTTGCAAGA CGACCAAGAGCGAAAGCATT TGCCAAGAATGGTTTCGTTA

A_Acorall GGCCTGCCGGGGGCATACGT ATTGCGAGGCGAGAGGTGAA ATTCTTGGACCCTTGCAAGA CGACCAAGAGCGAAAGCATT TGCCAAGAATGGTTTCGTTA

A_Bcorall GGCCTGCCGGGGGCATACGT ATTGCGAGGCGAGAGGTGAA ATTCTTGGACCCTTGCAAGA CGACCAAGAGCGAAAGCATT TGCCAAGAATGGTTTCGTTA

A_nsp1229 GGCCTGCCGGGGGCATACGT ATTGCGAGGCGAGAGGTGAA ATTCTTGGACCCTTGCAAGA CGACCAAGAGCGAAAGCATT TGCCAAGAATGGTTTCGTTA

A_nsp1396 GGCCTGCCGGGGGCATACGT ATTGCGAGGCGAGAGGTGAA ATTCTTGGACCCTTGCAAGA CGACCGAGAGCGAAAGCATT TGCCAAGAATGGTTTCGTTA

A_Aerythr nnnnnnnnnnnnnnnnnnnn nnnnnnnnnnnnnnnnnnnn nnnnnnnnnnnnnnnnnnnn nnnnnnnnnnnnnnnnnnnn nnnnnnnnnnnnnnnnnnnn

A_Berythr nnnnnnnnnnnnnnnnnnnn nnnnnnnnnnnnnnnnnnnn nnnnnnnnnnnnnnnnnnnn nnnnnnnnnnnnnnnnnnnn nnnnnnnnnnnnnnnnnnnn

A_AhovaSP GGCCTGCCGGGGGCATACGT ATTGCTTGGCGAGAGGTGAA ATTCTTGGACCCTTGCAAGA CGACCGAAAGCGAAAGCATT TGCCAAGAATGGTTTCGTTA

A_BhovaSP GGCCTGCCGGGGGCATACGT ATTGCTTGGCGAGAGGTGAA ATTCTTGGACCCTTGCAAGA CGACCGAAAGCGAAAGCATT TGCCAAGAATGGTTTCGTTA

A_ignipes GGCCTGCCGGGGGCATACGT ATTGCTTGGCGAGAGGTGAA ATTCTTGGACCCTTGCAAGA CGGCCGAAAGCGAAAGCATT TGCCAAGAATGGCTTCGTTA

A_Bcowani GGCCTGCCGGGGGCATACGT ATTGCGAGGCGAGAGGTGAA ATTCTTGGACCCTTGCAAGA CGACCGAGAGCGAAAGCATT TGCCAAGAATGGTTTCGTTA

A_Ccowani nnnnnnnnnnnnnnnnnnnn nnnnnnnnnnnnnnnnnnnn nnnnnnnnnnnnnnnnnnnn nnnnnnnnnnnnnnnnnnnn nnnnnnnnnnnnnnnnnnnn

A_Dcowani GGCCTGCCGGGGGCATACGT ATTGCGAGGCGAGAGGTGAA ATTCTTGGACCCTTGCAAGA CGACCGAGAGCGAAAGCATT TGCCAAGAATGGTTTCGTTA

A_Asangui nnnnnnnnnnnnnnnnnnnn nnnnnnnnnnnnnnnnnnnn nnnnnnnnnnnnnnnnnnnn nnnnnnnnnnnnnnnnnnnn nnnnnnnnnnnnnnnnnnnn

A_Bsangui nnnnnnnnnnnnnnnnnnnn nnnnnnnnnnnnnnnnnnnn nnnnnnnnnnnnnnnnnnnn nnnnnnnnnnnnnnnnnnnn nnnnnnnnnnnnnnnnnnnn

A_Csangui nnnnnnnnnnnnnnnnnnnn nnnnnnnnnnnnnnnnnnnn nnnnnnnnnnnnnnnnnnnn nnnnnnnnnnnnnnnnnnnn nnnnnnnnnnnnnnnnnnnn

A_Dsangui nnnnnnnnnnnnnnnnnnnn nnnnnnnnnnnnnnnnnnnn nnnnnnnnnnnnnnnnnnnn nnnnnnnnnnnnnnnnnnnn nnnnnnnnnnnnnnnnnnnn

Doratogon ATCAAGAACGAAAGTTAGAG GTTCGAAGGCGATCAGATAC CGCCCTAGTTCTAACCATAA ACGATGCCAACCAGCGATCC GCCGGAGTTCCTCCAATGAC

Mad_maxAS ATCAAGAACGAAAGTTAGAG GTTCGAAGGCGATCAGATAC CGCCCTAGTTCTAACTATAA ACGATGTCAACCGGCAATTA GAGTGCGTTCCTCCAATGAC

Mad_maxBS ATCAAGAACGAAAGTTAGAG GTTCGAAGGCGATCAGATAC CGCCCTAGTTCTAACTATAA ACGATGTCAACCGGCAATTA GAGTGCGTTCCTCCAATGAC

S_simplex ATCAAGAACGAAAGTTAGAG GTTCGAAGGCGATCAGATAC CGCCCTAGTTCTAACTATAA ACGATGCCAACCAGCGATCA GTATGCGTTCCTCAAATGAC

S_triareu ATCAAGAACGAAAGTTAGAG GTTMGAAGGCGATCAGATAC CGCCCTAGTTCTAACTATAA ACGATGCCAACCAGCGATCA GTATGCGTTCCTCAAATGAC

C_AsemiSP nnnnnnnnnnnnnnnnnnnn nnnnnnnnnnnnnnnnnnnn nnnnnnnnnnnnnnnnnnnn nnnnnnnnnnnnnnnnnnnn nnnnnnnnnnnnnnnnnnnn

C_BsemiSP ATCAAGAACGAAAGTTAGAG GTTCGAAGGCGATCAGATAC CGCCCTAGTTCTAACCATAA ACGATGTTAACCAGCGATCA GTGTGCGTTCCTCCAATGAC

A_Avampyr ATCAAGAACGAAAGTTAGAG GTTCGAAGGCGATCAGATAC CGCCCTAGTTCTAACCATAA ACGATGTTAACCAGCGATCA GCGTGCGTTCCTCCAATGAC

A_Bvampyr ATCAAGAACGAAAGTTAGAG GTTCGAAGGCGATCAGATAC CGCCCTAGTTCTAACCATAA ACGATGTTAACCAGCGATCA GCGTGCGTTCCTCCAATGAC

A_Cvampyr ATCAAGAACGAAAGTTAGAG GTTCGAAGGCGATCAGATAC CGCCCTAGTTCTAACCATAA ACGATGTTAACCAGCGATCA GCGTGCGTTCCTCCAATGAC

A_Dvampyr ATCAAGAACGAAAGTTAGAG GTTCGAAGGCGATCAGATAC CGCCCTAGTTCTAACCATAA ACGATGTTAACCAGCGATCA GCGTGCGTTCCTCCAATGAC

A_Ainfern ATCAAGAACGAAAGTTAGAG GTTCGAAGGCGATCAGATAC CGCCCTAGTTCTAACCATAA ACGATGTTAACCAGCGATCA GCGTGCGTTCCTCCAATGAC

A_Binfern ATCAAGAACGAAAGTTAGAG GTTCGAAGGCGATCAGATAC CGCCCTAGTTCTAACCATAA ACGATGTTAACCAGCGATCA GCGTGCGTTCCTCCAATGAC

A_Cinfern ATCAAGAACGAAAGTTAGAG GTTCGAAGGCGATCAGATAC CGCCCTAGTTCTAACCATAA ACGATGTTAACCAGCGATCA GCGTGCGTTCCTCCAATGAC

A_Dinfern ATCAAGAACGAAAGTTAGAG GTTCGAAGGCGATCAGATAC CGCCCTAGTTCTAACCATAA ACGATGTTAACCAGCGATCA GCGTGCGTTCCTCCAATGAC

A_Einfern ATCAAGAACGAAAGTTAGAG GTTCGAAGGCGATCAGATAC CGCCCTAGTTCTAACCATAA ACGATGTTAACCAGCGATCA GCGTGCGTTCCTCCAATGAC

A_Acorall ATCAAGAACGAAAGTTAGAG GTTCGAAGGCGATCAGATAC CGCCCTAGTTCTAACCATAA ACGATGTTAACCAGCGATCA GCGTGCGTTCCTCCAATGAC

A_Bcorall ATCAAGAACGAAAGTTAGAG GTTCGAAGGCGATCAGATAC CGCCCTAGTTCTAACCATAA ACGATGTTAACCAGCGATCA GCGTGCGTTCCTCCAATGAC

A_nsp1229 ATCAAGAACGAAAGTTAGAG GTTCGAAGGCGATCAGATAC CGCCCTAGTTCTAACCATAA ACGATGTTAACCAGCGATCA GCGTGCGTTCCTCCAATGAC

A_nsp1396 ATCAAGAACGAAAGTTAGAG GTTCGAAGGCGATCAGATAC CGCCCTAGTTCTAACCATAA ACGATGTTAACCAGCGATCA GCGTGCGTTCCTCCAATGAC

A_Aerythr nnnnnnnnnnnnnnnnnnnn nnnnnnnnnnnnnnnnnnnn nnnnnnnnnnnnnnnnnnnn nnnnnnnnnnnnnnnnnnnn nnnnnnnnnnnnnnnnnnnn

A_Berythr nnnnnnnnnnnnnnnnnnnn nnnnnnnnnnnnnnnnnnnn nnnnnnnnnnnnnnnnnnnn nnnnnnnnnnnnnnnnnnnn nnnnnnnnnnnnnnnnnnnn

A_AhovaSP ATCAAGAACGAAAGTTAGAG GTTCGAAGGCGATCAGATAC CGCCCTAGTTCTAACCATAA ACGATGTTAACCAGCGATCA GTGTGCGTTCCTCCAATGAC

A_BhovaSP ATCAAGAACGAAAGTTAGAG GTTCGAAGGCGATCAGATAC CGCCCTAGTTCTAACCATAA ACGATGTTAACCAGCGATCA GTGTGCGTTCCTCCAATGAC

A_ignipes ATCAAGAACGAAAGTTAGAG GTTCGAAGGCGATCAGATAC CGCCCTAGTTCTAACCATAA ACGATGTTAACCAGCGATCA GTGTGCGTTCCTCCAATGAC

A_Bcowani ATCAAGAACGAAAGTTAGAG GTTCGAAGGCGATCAGATAC CGCCCTAGTTCTAACCATAA ACGATGTTAACCAGCGATCA GCATGCGTTCCTCCAATGAC

A_Ccowani nnnnnnnnnnnnnnnnnnnn nnnnnnnnnnnnnnnnnnnn nnnnnnnnnnnnnnnnnnnn nnnnnnnnnnnnnnnnnnnn nnnnnnnnnnnnnnnnnnnn

A_Dcowani ATCAAGAACGAAAGTTAGAG GTTCGAAGGCGATCAGATAC CGCCCTAGTTCTAACCATAA ACGATGTTAACCAGCGATCA GCATGCGTTCCTCCAATGAC

A_Asangui nnnnnnnnnnnnnnnnnnnn nnnnnnnnnnnnnnnnnnnn nnnnnnnnnnnnnnnnnnnn nnnnnnnnnnnnnnnnnnnn nnnnnnnnnnnnnnnnnnnn

A_Bsangui nnnnnnnnnnnnnnnnnnnn nnnnnnnnnnnnnnnnnnnn nnnnnnnnnnnnnnnnnnnn nnnnnnnnnnnnnnnnnnnn nnnnnnnnnnnnnnnnnnnn

A_Csangui nnnnnnnnnnnnnnnnnnnn nnnnnnnnnnnnnnnnnnnn nnnnnnnnnnnnnnnnnnnn nnnnnnnnnnnnnnnnnnnn nnnnnnnnnnnnnnnnnnnn

A_Dsangui nnnnnnnnnnnnnnnnnnnn nnnnnnnnnnnnnnnnnnnn nnnnnnnnnnnnnnnnnnnn nnnnnnnnnnnnnnnnnnnn nnnnnnnnnnnnnnnnnnnn

Doratogon CCGGCCGGCAGCTT-CCGGG AAACCAAAGTATTTGGGTTC CGGGGGAAGTATGGTTGCAA AGCTGAAACTTAAAGGAATT GACGGAAGGGCACCACCAGG

Mad_maxAS GCCTCTGGCAGCTC-CCGGG AAACCAGAGTCTCTGGGTTC CTGGGGAAGTATGGTTGCAA AGCTGAAACTTAAAGGAATT GACGGAAGGGCACCACCAGG

Mad_maxBS GCCTCTGGCAGCTC-CCGGG AAACCAGAGTCTCTGGGTTC CTGGGGAAGTATGGTTGCAA AGCTGAAACTTAAAGGAATT GACGGAAGGGCACCACCAGG

S_simplex GCCGCTGGCAGCCC-CCGGA AAACCAGAGTGTCTGGGTTC CGGGGGTAGTATGGTTGCAA AGCTGAAACTTAAAGGAATT GACGGAAGGGCACCACCAGG

S_triareu GCCGCTGGCAGCCC-CCGGA AAACCAGAGTGTCTGGGTTC CGGGGGTAGTATGGTTGCAA AGCTGAAACTTAAAGGAATT GACGGAAGGGCACCACCAGG

C_AsemiSP nnnnnnnnnnnnnnnnnnnn nnnnnnnnnnnnnnnnnnnn nnnnnnnnnnnnnnnnnnnn nnnnnnnnnnnnnnnnnnnn nnnnnnnnnnnnnnnnnnnn

C_BsemiSP GCCGCTGGCAGCTCCCCGGG AAACCAGAGTCTCTGGATTC CTGGGGTAGTATGGTTGCAA AACTGAAACTTAAAGGAATT GACGGAAGGGCACCACCAGG

A_Avampyr GCCGCTGGCAGCTCCCCGGG AAACCAGAGTCTCTGGATTC CTGGGGTAGTATGGTTGCAA AACTGAAACTTAAAGGAATT GACGGAAGGGCACCACCAGG

A_Bvampyr GCCGCTGGCAGCTCCCCGGG AAACCAGAGTCTCTGGATTC CTGGGGTAGTATGGTTGCAA AACTGAAACTTAAAGGAATT GACGGAAGGGCACCACCAGG

A_Cvampyr GCCGCTGGCAGCTCCCCGGG AAACCAGAGTCTCTGGATTC CTGGGGTAGTATGGTTGCAA AACTGAAACTTAAAGGAATT GACGGAAGGGCACCACCAGG

A_Dvampyr GCCGCTGGCAGCTCCCCGGG AAACCAGAGTCTCTGGATTC CTGGGGTAGTATGGTTGCAA AACTGAAACTTAAAGGAATT GACGGAAGGGCACCACCAGG

A_Ainfern GCCGCTGGCAGCTCCCCGGG AAACCAGAGTCTCTGGATTC CTGGGGTAGTATGGTTGCAA AACTGAAACTTAAAGGAATT GACGGAAGGGCACCACCAGG

A_Binfern GCCGCTGGCAGCTCCCCGGG AAACCAGAGTCTCTGGATTC CTGGGGTAGTATGGTTGCAA AACTGAAACTTAAAGGAATT GACGGAAGGGCACCACCAGG

A_Cinfern GCCGCTGGCAGCTCCCCGGG AAACCAGAGTCTCTGGATTC CTGGGGTAGTATGGTTGCAA AACTGAAACTTAAAGGAATT GACGGAAGGGCACCACCAGG

A_Dinfern GCCGCTGGCAGCTCCCCGGG AAACCAGAGTCTCTGGATTC CTGGGGTAGTATGGTTGCAA AACTGAAACTTAAAGGAATT GACGGAAGGGCACCACCAGG

A_Einfern GCCGCTGGCAGCTCCCCGGG AAACCAGAGTCTCTGGATTC CTGGGGTAGTATGGTTGCAA AACTGAAACTTAAAGGAATT GACGGAAGGGCACCACCAGG

A_Acorall GCCGCTGGCAGCTCCCCGGG AAACCAGAGTCTCTGGATTC CTGGGGTAGTATGGTTGCAA AACTGAAACTTAAAGGAATT GACGGAAGGGCACCACCAGG

A_Bcorall GCCGCTGGCAGCTCCCCGGG AAACCAGAGTCTCTGGATTC CTGGGGTAGTATGGTTGCAA AACTGAAACTTAAAGGAATT GACGGAAGGGCACCACCAGG

A_nsp1229 GCCGCTGGCAGCTCCCCGGG AAACCAGAGTCTCTGGATTC CTGGGGTAGTATGGTTGCAA AACTGAAACTTAAAGGAATT GACGGAAGGGCACCACCAGG

A_nsp1396 GCCGCTGGCAGCTCCCCGGG AAACCAGAGTCTCTGGATTC CTGGGGTAGTATGGTTGCAA AACTGAAACTTAAAGGAATT GACGGAAGGGCACCACCAGG

A_Aerythr nnnnnnnnnnnnnnnnnnnn nnnnnnnnnnnnnnnnnnnn nnnnnnnnnnnnnnnnnnnn nnnnnnnnnnnnnnnnnnnn nnnnnnnnnnnnnnnnnnnn

A_Berythr nnnnnnnnnnnnnnnnnnnn nnnnnnnnnnnnnnnnnnnn nnnnnnnnnnnnnnnnnnnn nnnnnnnnnnnnnnnnnnnn nnnnnnnnnnnnnnnnnnnn

A_AhovaSP GCCGCTGGCAGCTTCCCGGG AAACCAGAGTCTCTGGATTC CTGGGGTAGTATGGTTGCAA AAGTGAAACTTAAAGGAATT GACGGAAGGGCACCACCAGG

A_BhovaSP GCCGCTGGCAGCTTCCCGGG AAACCAGAGTCTCTGGATTC CTGGGGTAGTATGGTTGCAA AAGTGAAACTTAAAGGAATT GACGGAAGGGCACCACCAGG

A_ignipes GCCGCTGGCAGCTCCCCGGG AAACCAGAGTCTCTGGATTC CTGGGGTAGTATGGTTGCAA AAGTGAAACTTAAAGGAATT GACGGAAGGGCACCACCAGG

A_Bcowani GCTGCTGGCAGCTCTCCGGG AAACCAGAGTCTCTGGATTC CTGGGGTAGTATGGTTGCAA AACTGAAACTTAAAGGAATT GACGGAAGGGCACCACCAGG

A_Ccowani nnnnnnnnnnnnnnnnnnnn nnnnnnnnnnnnnnnnnnnn nnnnnnnnnnnnnnnnnnnn nnnnnnnnnnnnnnnnnnnn nnnnnnnnnnnnnnnnnnnn

A_Dcowani GCTGCTGGCAGCTCTCCGGG AAACCAGAGTCTCTGGATTC CTGGGGTAGTATGGTTGCAA AACTGAAACTTAAAGGAATT GACGGAAGGGCACCACCAGG

A_Asangui nnnnnnnnnnnnnnnnnnnn nnnnnnnnnnnnnnnnnnnn nnnnnnnnnnnnnnnnnnnn nnnnnnnnnnnnnnnnnnnn nnnnnnnnnnnnnnnnnnnn

A_Bsangui nnnnnnnnnnnnnnnnnnnn nnnnnnnnnnnnnnnnnnnn nnnnnnnnnnnnnnnnnnnn nnnnnnnnnnnnnnnnnnnn nnnnnnnnnnnnnnnnnnnn

A_Csangui nnnnnnnnnnnnnnnnnnnn nnnnnnnnnnnnnnnnnnnn nnnnnnnnnnnnnnnnnnnn nnnnnnnnnnnnnnnnnnnn nnnnnnnnnnnnnnnnnnnn

A_Dsangui nnnnnnnnnnnnnnnnnnnn nnnnnnnnnnnnnnnnnnnn nnnnnnnnnnnnnnnnnnnn nnnnnnnnnnnnnnnnnnnn nnnnnnnnnnnnnnnnnnnn

Doratogon AGTGGAGCCTGCGGCTTAAT TTGACTCAACACGGGAAAAC TCACCCGGCCCGGACACTGG AAGGATTGACAGATTGAGAG CTCTTTCTTGATTCAGTGGG

Mad_maxAS AGTGGAGCCTGCGGCTTAAT TTGACTCAACACGGGGCAGC TCACTCGGCCCGGACACTCG AAGGATTGACAGATTGAGAG CTCTTTCTCGATTAAGTGGT

Mad_maxBS AGTGGAGCCTGCGGCTTAAT TTGACTCAACACGGGGCAGC TCACTCGGCCCGGACACTCG AAGGATTGACAGATTGAGAG CTCTTTCTCGATTAAGTGGT

S_simplex AGTGGAGCCTGCGGCTTAAT TTGACTCAACACGGGGCAGC TCACTCGGCCCGGACACTCG AAGGATTGACAGATTGAGAG CTCTTTCTTGATCTAATGGC

S_triareu AGTGGAGCCTGCGGCTTAAT TTGACTCAACACGGGGCAGC TCACTCGGCCCGGACACTCG AAGGATTGACAGATTGAGAG CTCTTTCTTGATCTAATGGC

C_AsemiSP nnnnnnnnnnnnnnnnnnnn nnnnnnnnnnnnnnnnnnnn nnnnnnnnnnnnnnnnnnnn nnnnnnnnnnnnnnnnnnnn nnnnnnnnnnnnnnnnnnnn

C_BsemiSP AGTGGAGCCTGCGGCTTAAT TTGACTCAACACGGGGCAGC TCACTCGGCCCGGACACTCG AAGGATTGACAGATTGAGAG CTCTTTCTTGATCTAGCGGC

A_Avampyr AGTGGAGCCTGCGGCTTAAT TTGACTCAACACGGGGCAGC TCACTCGGCCCGGACACTCG AAGGATTGACAGATTGAGAG CTCTTTCTTGATCTAATGGC

A_Bvampyr AGTGGAGCCTGCGGCTTAAT TTGACTCAACACGGGGCAGC TCACTCGGCCCGGACACTCG AAGGATTGACAGATTGAGAG CTCTTTCTTGATCTAATGGC

A_Cvampyr AGTGGAGCCTGCGGCTTAAT TTGACTCAACACGGGGCAGC TCACTCGGCCCGGACACTCG AAGGATTGACAGATTGAGAG CTCTTTCTTGATCTAATGGC

A_Dvampyr AGTGGAGCCTGCGGCTTAAT TTGACTCAACACGGGGCAGC TCACTCGGCCCGGACACTCG AAGGATTGACAGATTGAGAG CTCTTTCTTGATCTAATGGC

A_Ainfern AGTGGAGCCTGCGGCTTAAT TTGACTCAACACGGGGCAGC TCACTCGGCCCGGACACTCG AAGGATTGACAGATTGAGAG CTCTTTCTTGATCTAATGGC

A_Binfern AGTGGAGCCTGCGGCTTAAT TTGACTCAACACGGGGCAGC TCACTCGGCCCGGACACTCG AAGGATTGACAGATTGAGAG CTCTTTCTTGATCTAATGGC

A_Cinfern AGTGGAGCCTGCGGCTTAAT TTGACTCAACACGGGGCAGC TCACTCGGCCCGGACACTCG AAGGATTGACAGATTGAGAG CTCTTTCTTGATCTAATGGC

A_Dinfern AGTGGAGCCTGCGGCTTAAT TTGACTCAACACGGGGCAGC TCACTCGGCCCGGACACTCG AAGGATTGACAGATTGAGAG CTCTTTCTTGATCTAATGGC

A_Einfern AGTGGAGCCTGCGGCTTAAT TTGACTCAACACGGGGCAGC TCACTCGGCCCGGACACTCG AAGGATTGACAGATTGAGAG CTCTTTCTTGATCTAATGGC

A_Acorall AGTGGAGCCTGCGGCTTAAT TTGACTCAACACGGGGCAGC TCACTCGGCCCGGACACTCG AAGGATTGACAGATTGAGAG CTCTTTCTTGATCTAATGGC

A_Bcorall AGTGGAGCCTGCGGCTTAAT TTGACTCAACACGGGGCAGC TCACTCGGCCCGGACACTCG AAGGATTGACAGATTGAGAG CTCTTTCTTGATCTAATGGC

A_nsp1229 AGTGGAGCCTGCGGCTTAAT TTGACTCAACACGGGGCAGC TCACTCGGCCCGGACACTCG AAGGATTGACAGATTGAGAG CTCTTTCTTGATCTAATGGC

A_nsp1396 AGTGGAGCCTGCGGCTTAAT TTGACTCAACACGGGGCAGC TCACTCGGCCCGGACACTCG AAGGATTGACAGATTGAGAG CTCTTTCTTGATCTAATGGC

A_Aerythr nnnnnnnnnnnnnnnnnnnn nnnnnnnnnnnnnnnnnnnn nnnnnnnnnnnnnnnnnnnn nnnnnnnnnnnnnnnnnnnn nnnnnnnnnnnnnnnnnnnn

A_Berythr nnnnnnnnnnnnnnnnnnnn nnnnnnnnnnnnnnnnnnnn nnnnnnnnnnnnnnnnnnnn nnnnnnnnnnnnnnnnnnnn nnnnnnnnnnnnnnnnnnnn

A_AhovaSP AGTGGAGCCTGCGGCTTAAT TTGACTCAACACGGGGCAGC TCACTCGGCCCGGACATTCG AAGGATTGACAGATTGAGAG CTCTTTCTTGATCTAATGGC

A_BhovaSP AGTGGAGCCTGCGGCTTAAT TTGACTCAACACGGGGCAGC TCACTCGGCCCGGACATTCG AAGGATTGACAGATTGAGAG CTCTTTCTTGATCTAATGGC

A_ignipes AGTGGAGCCTGCGGCTTAAT TTGACTCAACACGGGGCAGC TCACTCGGCCCGGACATTCG AAGGATTGACAGATTGAGAG CTCTTTCTTGATCTAATGGC

A_Bcowani AGTGGAGCCTGCGGCTTAAT TTGACTCAACACGGGGCAGC TCACTCGGCCCGGACACTCG AAGGATTGACAGATTGAGAG CTCTTTCTTGATCTAATGGC

A_Ccowani nnnnnnnnnnnnnnnnnnnn nnnnnnnnnnnnnnnnnnnn nnnnnnnnnnnnnnnnnnnn nnnnnnnnnnnnnnnnnnnn nnnnnnnnnnnnnnnnnnnn

A_Dcowani AGTGGAGCCTGCGGCTTAAT TTGACTCAACACGGGGCAGC TCACTCGGCCCGGACACTCG AAGGATTGACAGATTGAGAG CTCTTTCTTGATCTAATGGC

A_Asangui nnnnnnnnnnnnnnnnnnnn nnnnnnnnnnnnnnnnnnnn nnnnnnnnnnnnnnnnnnnn nnnnnnnnnnnnnnnnnnnn nnnnnnnnnnnnnnnnnnnn

A_Bsangui nnnnnnnnnnnnnnnnnnnn nnnnnnnnnnnnnnnnnnnn nnnnnnnnnnnnnnnnnnnn nnnnnnnnnnnnnnnnnnnn nnnnnnnnnnnnnnnnnnnn

A_Csangui nnnnnnnnnnnnnnnnnnnn nnnnnnnnnnnnnnnnnnnn nnnnnnnnnnnnnnnnnnnn nnnnnnnnnnnnnnnnnnnn nnnnnnnnnnnnnnnnnnnn

A_Dsangui nnnnnnnnnnnnnnnnnnnn nnnnnnnnnnnnnnnnnnnn nnnnnnnnnnnnnnnnnnnn nnnnnnnnnnnnnnnnnnnn nnnnnnnnnnnnnnnnnnnn

Doratogon TGGTGGTGCATGGCCGTTCT TAGTTGGTGGAGCGATTTGT CTGGTTAATTCCGATAACGA ACGAGACTCTAGCCTGCTAA ATAGGTGGTCGATTCTCTGA

Mad_maxAS TGGTGGTGCATGGCCGTTCT TAGTTGGTGGAGTGATTTGT CTGGTTAATTCCGATAACGA ACGAGACTCTGGCCTACTAA ATAGAAAGTCGATAGGTC--

Mad_maxBS TGGTGGTGCATGGCCGTTCT TAGTTGGTGGAGTGATTTGT CTGGTTAATTCCGATAACGA ACGAGACTCTGGCCTACTAA ATAGAAAGTCGATAGGTC--

S_simplex TGGTGGTGCATGGCCGTTCT TAGTTGGTGGAGTGATTTGT CTGGTTAATTCCGATAACGA ACGAGACTCTGGCCTACTAA ATAGAAAGTCGATAGCCCAA

S_triareu TGGTGGTGCATGGCCGTTCT TAGTTGGTGGAGTGATTTGT CTGGTTAATTCCGATAACGA ACGAGACTCTGGCCTACTAA ATAGAAAGTCGATAGCCCAA

C_AsemiSP nnnnnnnnnnnnnnnnnnnn nnnnnnnnnnnnnnnnnnnn nnnnnnnnnnnnnnnnnnnn nnnnnnnnnnnnnnnnnnnn nnnnnnnnnnnnnnnnnnnn

C_BsemiSP TGGTGGTGCATGGCCGTTCT TAGTTGGTGGAGTGATTTGT CTGGTTAATTCCGATAACGA ACGAGACTCTGGCCTACTAA ATAGAAAGTCGATAATCCAA

A_Avampyr TGGTGGTGCATGGCCGTTCT TAGTTGGTGGAGTGATTTGT CTGGTTAATTCCGATAACGA ACGAGACTCTGGCCTACTAA ATAGAAAGTCGATT-CCCAA

A_Bvampyr TGGTGGTGCATGGCCGTTCT TAGTTGGTGGAGTGATTTGT CTGGTTAATTCCGATAACGA ACGAGACTCTGGCCTACTAA ATAGAAAGTCGATT-CCCAA

A_Cvampyr TGGTGGTGCATGGCCGTTCT TAGTTGGTGGAGTGATTTGT CTGGTTAATTCCGATAACGA ACGAGACTCTGGCCTACTAA ATAGAAAGTCGATT-CCCAA

A_Dvampyr TGGTGGTGCATGGCCGTTCT TAGTTGGTGGAGTGATTTGT CTGGTTAATTCCGATAACGA ACGAGACTCTGGCCTACTAA ATAGAAAGTCGATT-CCCAA

A_Ainfern TGGTGGTGCATGGCCGTTCT TAGTTGGTGGAGTGATTTGT CTGGTTAATTCCGATAACGA ACGAGACTCTGGCCTACTAA ATAGAAAGTCGATT-CCCAA

A_Binfern TGGTGGTGCATGGCCGTTCT TAGTTGGTGGAGTGATTTGT CTGGTTAATTCCGATAACGA ACGAGACTCTGGCCTACTAA ATAGAAAGTCGATT-CCCAA

A_Cinfern TGGTGGTGCATGGCCGTTCT TAGTTGGTGGAGTGATTTGT CTGGTTAATTCCGATAACGA ACGAGACTCTGGCCTACTAA ATAGAAAGTCGATT-CCCAA

A_Dinfern TGGTGGTGCATGGCCGTTCT TAGTTGGTGGAGTGATTTGT CTGGTTAATTCCGATAACGA ACGAGACTCTGGCCTACTAA ATAGAAAGTCGATT-CCCAA

A_Einfern TGGTGGTGCATGGCCGTTCT TAGTTGGTGGAGTGATTTGT CTGGTTAATTCCGATAACGA ACGAGACTCTGGCCTACTAA ATAGAAAGTCGATT-CCCAA

A_Acorall TGGTGGTGCATGGCCGTTCT TAGTTGGTGGAGTGATTTGT CTGGTTAATTCCGATAACGA ACGAGACTCTGGCCTGCTAA ATAGAAAGTCGATT-CCCAA

A_Bcorall TGGTGGTGCATGGCCGTTCT TAGTTGGTGGAGTGATTTGT CTGGTTAATTCCGATAACGA ACGAGACTCTGGCCTGCTAA ATAGAAAGTCGATT-CCCAA

A_nsp1229 TGGTGGTGCATGGCCGTTCT TAGTTGGTGGAGTGATTTGT CTGGTTAATTCCGATAACGA ACGAGACTCTGGCCTACTAA ATAGAAAGTCGATTGCCC-A

A_nsp1396 TGGTGGTGCATGGCCGTTCT TAGTTGGTGGAGTGATTTGT CTGGTTAATTCCGATAACGA ACGAGACTCTGGCCTACTAA ATAGAAAGTCGATTGCCC-A

A_Aerythr nnnnnnnnnnnnnnnnnnnn nnnnnnnnnnnnnnnnnnnn nnnnnnnnnnnnnnnnnnnn nnnnnnnnnnnnnnnnnnnn nnnnnnnnnnnnnnnnnnnn

A_Berythr nnnnnnnnnnnnnnnnnnnn nnnnnnnnnnnnnnnnnnnn nnnnnnnnnnnnnnnnnnnn nnnnnnnnnnnnnnnnnnnn nnnnnnnnnnnnnnnnnnnn

A_AhovaSP TGGTGGTGCATGGCCGTTCT TAGTTGGTGGAGTGATTTGT CTGGTTAATTCCGATAACGA ACGAGACTCTGGCCTACTAA ATAGAAAGTCGATTGCCCAA

A_BhovaSP TGGTGGTGCATGGCCGTTCT TAGTTGGTGGAGTGATTTGT CTGGTTAATTCCGATAACGA ACGAGACTCTGGCCTACTAA ATAGAAAGTCGATTGCCCAA

A_ignipes TGGTGGTGCATGGCCGTTCT TAGTTGGTGGAGTGATTTGT CTGGTTAATTCCGATAACGA ACGAGACTCTGGCCTACTAA ATAGAAAGTCGATT-CCCAA

A_Bcowani TGGTGGTGCATGGCCGTTCT TAGTTGGTGGAGTGATTTGT CTGGTTAATTCCGATAACGA ACGAGACTCTGGCCTACTAA ATAGAAAGTCGATTGCCC-A

A_Ccowani nnnnnnnnnnnnnnnnnnnn nnnnnnnnnnnnnnnnnnnn nnnnnnnnnnnnnnnnnnnn nnnnnnnnnnnnnnnnnnnn nnnnnnnnnnnnnnnnnnnn

A_Dcowani TGGTGGTGCATGGCCGTTCT TAGTTGGTGGAGTGATTTGT CTGGTTAATTCCGATAACGA ACGAGACTCTGGCCTACTAA ATAGAAAGTCGATTGCCC-A

A_Asangui nnnnnnnnnnnnnnnnnnnn nnnnnnnnnnnnnnnnnnnn nnnnnnnnnnnnnnnnnnnn nnnnnnnnnnnnnnnnnnnn nnnnnnnnnnnnnnnnnnnn

A_Bsangui nnnnnnnnnnnnnnnnnnnn nnnnnnnnnnnnnnnnnnnn nnnnnnnnnnnnnnnnnnnn nnnnnnnnnnnnnnnnnnnn nnnnnnnnnnnnnnnnnnnn

A_Csangui nnnnnnnnnnnnnnnnnnnn nnnnnnnnnnnnnnnnnnnn nnnnnnnnnnnnnnnnnnnn nnnnnnnnnnnnnnnnnnnn nnnnnnnnnnnnnnnnnnnn

A_Dsangui nnnnnnnnnnnnnnnnnnnn nnnnnnnnnnnnnnnnnnnn nnnnnnnnnnnnnnnnnnnn nnnnnnnnnnnnnnnnnnnn nnnnnnnnnnnnnnnnnnnn

Doratogon AAGTCGAC--CTGCTCTTCT TAGAGGGACAAGCGGCGCTC AGCCGCACGAA--ATTGAGC AATAACAGGTCTGTGATGCC CTTAGATGTCCGGGGCCGCA

Mad_maxAS TGGTCGACTGATAATCTTCT TAGAGGGACAGGCGGCTCTC AGCCGCAAGAAG--TGGAGC GATAACAGGTCTGTGATGCC CTTAGATGTCCGAGGCTGCA

Mad_maxBS TGGTCGACTGATAATCTTCT TAGAGGGACAGGCGGCTCTC AGCCGCAAGAAG--TGGAGC GATAACAGGTCTGTGATGCC CTTAGATGTCCGAGGCTGCA

S_simplex --GTCGACTACTG-TCTTCT TAGAGGGACAGGCGGAACAT AGCCGCAAGAAGTAAAGAGC GATAACAGGTCTGTGATGCC CCTAGATGTCCGAGGCTGCA

S_triareu --GTCGACTACTG-TCTTCT TAGAGGGACAGGCGGAACAT AGCCGCAAGAAGTAAAGAGC GATAACAGGTCTGTGATGCC CCTAGATGTCCGAGGCTGCA

C_AsemiSP nnnnnnnnnnnnnnnnnnnn nnnnnnnnnnnnnnnnnnnn nnnnnnnnnnnnnnnnnnnn nnnnnnnnnnnnnnnnnnnn nnnnnnnnnnnnnnnnnnnn

C_BsemiSP GGGTCGACTGCCG-TCTTCT TAGAGGGACAGGCGGATTAT AGCCGCAAGAAGTATAGAGC GATAACAGGTCTGTGATGCC CCTAGATGTCCGAGGCTGCA

A_Avampyr TGGTCGACTGCTG-TCTTCT TAGAGGGACAGGCGGATTAT AGCCGCAAGAAGAACAGAGC GATAACAGGTCTGTGATGCC CCTAGATGTCCGAGGCTGCA

A_Bvampyr TGGTCGACTGCTG-TCTTCT TAGAGGGACAGGCGGATTAT AGCCGCAAGAAGAACAGAGC GATAACAGGTCTGTGATGCC CCTAGATGTCCGAGGCTGCA

A_Cvampyr TGGTCGACTGCTG-TCTTCT TAGAGGGACAGGCGGATTAT AGCCGCAAGAAGAACAGAGC GATAACAGGTCTGTGATGCC CCTAGATGTCCGAGGCTGCA

A_Dvampyr TGGTCGACTGCTG-TCTTCT TAGAGGGACAGGCGGATTAT AGCCGCAAGAAGAACAGAGC GATAACAGGTCTGTGATGCC CCTAGATGTCCGAGGCTGCA

A_Ainfern TGGTCGACTGCTG-TCTTCT TAGAGGGACAGGCGGATTAT AGCCGCAAGAAGAACAGAGC GATAACAGGTCTGTGATGCC CCTAGATGTCCGAGGCTGCA

A_Binfern TGGTCGACTGCTG-TCTTCT TAGAGGGACAGGCGGATTAT AGCCGCAAGAAGAACAGAGC GATAACAGGTCTGTGATGCC CCTAGATGTCCGAGGCTGCA

A_Cinfern TGGTCGACTGCTG-TCTTCT TAGAGGGACAGGCGGATTAT AGCCGCAAGAAGAACAGAGC GATAACAGGTCTGTGATGCC CCTAGATGTCCGAGGCTGCA

A_Dinfern TGGTCGACTGCTG-TCTTCT TAGAGGGACAGGCGGATTAT AGCCGCAAGAAGAACAGAGC GATAACAGGTCTGTGATGCC CCTAGATGTCCGAGGCTGCA

A_Einfern TGGTCGACTGCTG-TCTTCT TAGAGGGACAGGCGGATTAT AGCCGCAAGAAGAACAGAGC GATAACAGGTCTGTGATGCC CCTAGATGTCCGAGGCTGCA

A_Acorall TGGTCGACTGCTG-TCTTCT TAGAGGGACAGGCGGATTAT AGCCGCAAGAAGAACAGAGC GATAACAGGTCTGTGATGCC CCTAGATGTCCGAGGCTGCA

A_Bcorall TGGTCGACTGCTG-TCTTCT TAGAGGGACAGGCGGATTAT AGCCGCAAGAAGAACAGAGC GATAACAGGTCTGTGATGCC CCTAGATGTCCGAGGCTGCA

A_nsp1229 TGGTCGACTGCTG-TCTTCT TAGAGGGACAGGCGGATTAT AGCCGCAAGAAGAACAGAGC GATAACAGGTCTGTGATGCC CCTAGATGTCCGAGGCTGCA

A_nsp1396 TGGTCGACTGCCG-TCTTCT TAGAGGGACAGGCGGATTAT AGCCGCAAGAAGAACAGAGC GATAACAGGTCTGTGATGCC CCTAGATGTCCGAGGCTGCA

A_Aerythr nnnnnnnnnnnnnnnnnnnn nnnnnnnnnnnnnnnnnnnn nnnnnnnnnnnnnnnnnnnn nnnnnnnnnnnnnnnnnnnn nnnnnnnnnnnnnnnnnnnn

A_Berythr nnnnnnnnnnnnnnnnnnnn nnnnnnnnnnnnnnnnnnnn nnnnnnnnnnnnnnnnnnnn nnnnnnnnnnnnnnnnnnnn nnnnnnnnnnnnnnnnnnnn

A_AhovaSP TGGTCGACTGCTG-TCTTCT TAGAGGGACAGGCGGATTAT AGCCGCAAGAAGAACAGAGC GATAACAGGTCTGTGATGCC CCTAGATGTCCGAGGCTGCA

A_BhovaSP TGGTCGACTGCTG-TCTTCT TAGAGGGACAGGCGGATTAT AGCCGCAAGAAGAACAGAGC GATAACAGGTCTGTGATGCC CCTAGATGTCCGAGGCTGCA

A_ignipes TGGTCGACTGCTG-TCTTCT TAGAGGGACAGGCGGATTAT AGCCGCAAGAAGAACAGAGC GATAACAGGTCTGTGATGCC CCTAGATGTCCGAGGCTGCA

A_Bcowani TGGTCGACTGCTG-TCTTCT TAGAGGGACAGGCGGATTAT AGCCGCAAGAAGAACAGAGC GATAACAGGTCTGTGATGCC CCTAGATGTCCGAGGCTGCA

A_Ccowani nnnnnnnnnnnnnnnnnnnn nnnnnnnnnnnnnnnnnnnn nnnnnnnnnnnnnnnnnnnn nnnnnnnnnnnnnnnnnnnn nnnnnnnnnnnnnnnnnnnn

A_Dcowani TGGTCGACTGCTG-TCTTCT TAGAGGGACAGGCGGATTAT AGCCGCAAGAAGAACAGAGC GATAACAGGTCTGTGATGCC CCTAGATGTCCGAGGCTGCA

A_Asangui nnnnnnnnnnnnnnnnnnnn nnnnnnnnnnnnnnnnnnnn nnnnnnnnnnnnnnnnnnnn nnnnnnnnnnnnnnnnnnnn nnnnnnnnnnnnnnnnnnnn

A_Bsangui nnnnnnnnnnnnnnnnnnnn nnnnnnnnnnnnnnnnnnnn nnnnnnnnnnnnnnnnnnnn nnnnnnnnnnnnnnnnnnnn nnnnnnnnnnnnnnnnnnnn

A_Csangui nnnnnnnnnnnnnnnnnnnn nnnnnnnnnnnnnnnnnnnn nnnnnnnnnnnnnnnnnnnn nnnnnnnnnnnnnnnnnnnn nnnnnnnnnnnnnnnnnnnn

A_Dsangui nnnnnnnnnnnnnnnnnnnn nnnnnnnnnnnnnnnnnnnn nnnnnnnnnnnnnnnnnnnn nnnnnnnnnnnnnnnnnnnn nnnnnnnnnnnnnnnnnnnn

Doratogon CGCGCGCTACACTGAAGGAA TCAACGTGTGTTTGCCCCCG T---CCGGAAGGACAGGGTA ATCCGTTGAACCTCCTTCGT GATAGGGACTGGGGCTTGAA

Mad_maxAS CGCGGGCTACACTGAAGGGG TCAGCGTGTCGATAGGCCCG G-GACCGCGAGGTCTGGGTA ATCCGCTGAACTTCCTTCGT GAAAGGGACTGGGGCTTGAA

Mad_maxBS CGCGGGCTACACTGAAGGGG TCAGCGTGTCGATAGGCCCG G-GACCGCGAGGTCTGGGTA ATCCGCTGAACTTCCTTCGT GAAAGGGACTGGGGCTTGAA

S_simplex CGCGGGCTACACTGAAGGAG TCAGCGTGTCGAGAACCCCT T-GACCGCGAGGTCGGGGTA ATCCGCTGAACTTCCTTCGT GAAAGGGACTGGGGCTTGAA

S_triareu CGCGGGCTACACTGAAGGAG TCAGCGTGTCGAGAACCCCT T-GACCGCGAGGTCGGGGTA ATCCGCTGAACTTCCTTCGT GAAAGGGACTGGGGCTTGAA

C_AsemiSP nnnnnnnnnnnnnnnnnnnn nnnnnnnnnnnnnnnnnnnn nnnnnnnnnnnnnnnnnnnn nnnnnnnnnnnnnnnnnnnn nnnnnnnnnnnnnnnnnnnn

C_BsemiSP CGCGGGCTACACTGAAGGAG TCAGCGTGTCGAGAATCCCG T-GACCGCGAGGTCTGGGTA ATCCGCTGAACTTCCTTCGT GAAAGGGACTGGGGCTTGAA

A_Avampyr CGCGGGCTACACTGAAGGAG TCAGCGTGTCGAGAATCCCG TCGACCGCGAGGTCTGGGTA ATCCGCTGAACTTCCTTCGT GAAAGGGACTGGGGCTTGAA

A_Bvampyr CGCGGGCTACACTGAAGGAG TCAGCGTGTCGAGAATCCCG TCGACCGCGAGGTCTGGGTA ATCCGCTGAACTTCCTTCGT GAAAGGGACTGGGGCTTGAA

A_Cvampyr CGCGGGCTACACTGAAGGAG TCAGCGTGTCGAGAATCCCG TCGACCGCGAGGTCTGGGTA ATCCGCTGAACTTCCTTCGT GAAAGGGACTGGGGCTTGAA

A_Dvampyr CGCGGGCTACACTGAAGGAG TCAGCGTGTCGAGAATCCCG TCGACCGCGAGGTCTGGGTA ATCCGCTGAACTTCCTTCGT GAAAGGGACTGGGGCTTGAA

A_Ainfern CGCGGGCTACACTGAAGGAG TCAGCGTGTCGAGAATCCCG TCGACCGCGAGGTCTGGGTA ATCCGCTGAACTTCCTTCGT GAAAGGGACTGGGGCTTGAA

A_Binfern CGCGGGCTACACTGAAGGAG TCAGCGTGTCGAGAATCCCG TCGACCGCGAGGTCTGGGTA ATCCGCTGAACTTCCTTCGT GAAAGGGACTGGGGCTTGAA

A_Cinfern CGCGGGCTACACTGAAGGAG TCAGCGTGTCGAGAATCCCG TCGACCGCGAGGTCTGGGTA ATCCGCTGAACTTCCTTCGT GAAAGGGACTGGGGCTTGAA

A_Dinfern CGCGGGCTACACTGAAGGAG TCAGCGTGTCGAGAATCCCG TCGACCGCGAGGTCTGGGTA ATCCGCTGAACTTCCTTCGT GAAAGGGACTGGGGCTTGAA

A_Einfern CGCGGGCTACACTGAAGGAG TCAGCGTGTCGAGAATCCCG TCGACCGCGAGGTCTGGGTA ATCCGCTGAACTTCCTTCGT GAAAGGGACTGGGGCTTGAA

A_Acorall CGCGGGCTACACTGAAGGAG TCAGCGTGTCGAGAATCCCG TCGACCGCGAGGTCTGGGTA ATCCGCTGAACTTCCTTCGT GAAAGGGACTGGGGCTTGAA

A_Bcorall CGCGGGCTACACTGAAGGAG TCAGCGTGTCGAGAATCCCG TCGACCGCGAGGTCTGGGTA ATCCGCTGAACTTCCTTCGT GAAAGGGACTGGGGCTTGAA

A_nsp1229 CGCGGGCTACACTGAAGGAG TCAGCGTGTCGAGAATCCCG TCGACCGCGAGGTCTGGGTA ATCCGCTGAACTTCCTTCGT GAAAGGGACTGGGGCTTGAA

A_nsp1396 CGCGGGCTACACTGAAGGAG TCAGCGTGTCGAGAATCCCG TCGACCGCGAGGTCTGGGTA ATCCGCTGAACTTCCTTCGT GAAAGGGACTGGGGCTTGAA

A_Aerythr nnnnnnnnnnnnnnnnnnnn nnnnnnnnnnnnnnnnnnnn nnnnnnnnnnnnnnnnnnnn nnnnnnnnnnnnnnnnnnnn nnnnnnnnnnnnnnnnnnnn

A_Berythr nnnnnnnnnnnnnnnnnnnn nnnnnnnnnnnnnnnnnnnn nnnnnnnnnnnnnnnnnnnn nnnnnnnnnnnnnnnnnnnn nnnnnnnnnnnnnnnnnnnn

A_AhovaSP CGCGGGCTACACTGAAGGAG TCAGCGTGTCGAGAATCCCG TCGACCGCGAGGTCCGGGTA ATCCGCTGAACTTCCTTCGT GAAAGGGACTGGGGCTTGAA

A_BhovaSP CGCGGGCTACACTGAAGGAG TCAGCGTGTCGAGAATCCCG TCGACCGCGAGGTCCGGGTA ATCCGCTGAACTTCCTTCGT GAAAGGGACTGGGGCTTGAA

A_ignipes CGCGGGCTACAATGAAGGAG TCAGCGTGTCGAGAA-CCCG TCGACCGCGAGGTCCGGGTA ATCCGCTGAACTTCCTTCGT GAAAGGGACTGGGGCTTGAA

A_Bcowani CGCGGGCTACACTGAAGGAG TCAGCGTGTCGAGAATCCCG TCGACCGCGAGGTCTGGGTA ATCCGCTGAACTTCCTTCGT GAAAGGGACTGGGGCTTGAA

A_Ccowani nnnnnnnnnnnnnnnnnnnn nnnnnnnnnnnnnnnnnnnn nnnnnnnnnnnnnnnnnnnn nnnnnnnnnnnnnnnnnnnn nnnnnnnnnnnnnnnnnnnn

A_Dcowani CGCGGGCTACACTGAAGGAG TCAGCGTGTCGAGAATCCCG TCGACCGCGAGGTCTGGGTA ATCCGCTGAACTTCCTTCGT GAAAGGGACTGGGGCTTGAA

A_Asangui nnnnnnnnnnnnnnnnnnnn nnnnnnnnnnnnnnnnnnnn nnnnnnnnnnnnnnnnnnnn nnnnnnnnnnnnnnnnnnnn nnnnnnnnnnnnnnnnnnnn

A_Bsangui nnnnnnnnnnnnnnnnnnnn nnnnnnnnnnnnnnnnnnnn nnnnnnnnnnnnnnnnnnnn nnnnnnnnnnnnnnnnnnnn nnnnnnnnnnnnnnnnnnnn

A_Csangui nnnnnnnnnnnnnnnnnnnn nnnnnnnnnnnnnnnnnnnn nnnnnnnnnnnnnnnnnnnn nnnnnnnnnnnnnnnnnnnn nnnnnnnnnnnnnnnnnnnn

A_Dsangui nnnnnnnnnnnnnnnnnnnn nnnnnnnnnnnnnnnnnnnn nnnnnnnnnnnnnnnnnnnn nnnnnnnnnnnnnnnnnnnn nnnnnnnnnnnnnnnnnnnn

Doratogon ATTCTTGCCCACGAACGAGG AATTCCCAGTAAGCGCGAGT CATAAGCTCGTGTTGATTAC GTCCCTGCCCTTTGTACACA CCGCCCGTCGCTACTACCGA

Mad_maxAS ACTATAGCCCACGAACGAGG AATTCCTGGTAAGGGTGAGT CATCAACTCGCCCTGACTAC GTCCCTGCCCTTTGTACACA CCGCCCGTCGCTACTACCGA

Mad_maxBS ACTATAGCCCACGAACGAGG AATTCCTGGTAAGGGTGAGT CATCAACTCGCCCTGACTAC GTCCCTGCCCTTTGTACACA CCGCCCGTCGCTACTACCGA

S_simplex ACTATAGCCCACGAACGAGG AATTCCTGGTAAGGGTGAGT CACCAACTCGCCCTGACTAT GTCCCTGCCCTTTGTACACA CCGCCCGTCGCTACTACCGA

S_triareu ACTATAGCCCACGAACGAGG AATTCCTGGTAAGGGTGAGT CACCAACTCGCCCTGACTAT GTCCCTGCCCTTTGTACACA CCGCCCGTCGCTACTACCGA

C_AsemiSP nnnnnnnnnnnnnnnnnnnn nnnnnnnnnnnnnnnnnnnn nnnnnnnnnnnnnnnnnnnn nnnnnnnnnnnnnnnnnnnn nnnnnnnnnnnnnnnnnnnn

C_BsemiSP ACTGTAGCCCACGAACGAGG AATTCCTGGTAAGGGCGAGT CACCAACTCGCCCTGACTAT GTCCCTGCCCTTTGTACACA CCGCCCGTCGCTACTACCGA

A_Avampyr ACTATAGCCCACAAACGAGG AATTCCTGGTAAGGGCGAGT CACCAACTCGCCCTGACTAT GTCCCTGCCCTTTGTACACA CCGCCCGTCGCTACTACCGA

A_Bvampyr ACTATAGCCCACAAACGAGG AATTCCTGGTAAGGGCGAGT CACCAACTCGCCCTGACTAT GTCCCTGCCCTTTGTACACA CCGCCCGTCGCTACTACCGA

A_Cvampyr ACTATAGCCCACAAACGAGG AATTCCTGGTAAGGGCGAGT CACCAACTCGCCCTGACTAT GTCCCTGCCCTTTGTACACA CCGCCCGTCGCTACTACCGA

A_Dvampyr ACTATAGCCCACAAACGAGG AATTCCTGGTAAGGGCGAGT CACCAACTCGCCCTGACTAT GTCCCTGCCCTTTGTACACA CCGCCCGTCGCTACTACCGA

A_Ainfern ACTATAGCCCACAAACGAGG AATTCCTGGTAAGGGCGAGT CACCAACTCGCCCTGACTAT GTCCCTGCCCTTTGTACACA CCGCCCGTCGCTACTACCGA

A_Binfern ACTATAGCCCACAAACGAGG AATTCCTGGTAAGGGCGAGT CACCAACTCGCCCTGACTAT GTCCCTGCCCTTTGTACACA CCGCCCGTCGCTACTACCGA

A_Cinfern ACTATAGCCCACAAACGAGG AATTCCTGGTAAGGGCGAGT CACCAACTCGCCCTGACTAT GTCCCTGCCCTTTGTACACA CCGCCCGTCGCTACTACCGA

A_Dinfern ACTATAGCCCACAAACGAGG AATTCCTGGTAAGGGCGAGT CACCAACTCGCCCTGACTAT GTCCCTGCCCTTTGTACACA CCGCCCGTCGCTACTACCGA

A_Einfern ACTATAGCCCACAAACGAGG AATTCCTGGTAAGGGCGAGT CACCAACTCGCCCTGACTAT GTCCCTGCCCTTTGTACACA CCGCCCGTCGCTACTACCGA

A_Acorall ACTATAGCCCACAAACGAGG AATTCCTGGTAAGGGCGAGT CACCAACTCGCCCTGACTAT GTCCCTGCCCTTTGTACACA CCGCCCGTCGCTACTACCGA

A_Bcorall ACTATAGCCCACAAACGAGG AATTCCTGGTAAGGGCGAGT CACCAACTCGCCCTGACTAT GTCCCTGCCCTTTGTACACA CCGCCCGTCGCTACTACCGA

A_nsp1229 ACTATAGCCCACAAACGAGG AATTCCTGGTAAGGGCGAGT CACCAACTCGCCCTGACTAT GTCCCTGCCCTTTGTACACA CCGCCCGTCGCTACTACCGA

A_nsp1396 ACTATAGCCCACGAACGAGG AATTCCTGGTAAGGGCGAGT CACCAACTCGCCCTGACTAT GTCCCTGCCCTTTGTACACA CCGCCCGTCGCTACTACCGA

A_Aerythr nnnnnnnnnnnnnnnnnnnn nnnnnnnnnnnnnnnnnnnn nnnnnnnnnnnnnnnnnnnn nnnnnnnnnnnnnnnnnnnn nnnnnnnnnnnnnnnnnnnn

A_Berythr nnnnnnnnnnnnnnnnnnnn nnnnnnnnnnnnnnnnnnnn nnnnnnnnnnnnnnnnnnnn nnnnnnnnnnnnnnnnnnnn nnnnnnnnnnnnnnnnnnnn

A_AhovaSP ACTATAGCCCACGAACGAGG AATTCCTGGTAAGGGTGAGT CACCAACTCGCCCTGACTAT GTCCCTGCCCTTTGTACACA CCGCCCGTCGCTACTACCGA

A_BhovaSP ACTATAGCCCACGAACGAGG AATTCCTGGTAAGGGTGAGT CACCAACTCGCCCTGACTAT GTCCCTGCCCTTTGTACACA CCGCCCGTCGCTACTACCGA

A_ignipes ACTGTAGCCCACGAACGAGG AATTCCTGGTAAGGGTGAGT CACCAACTCGCCCTGACTAT GTCCCTGCCCTTTGTACACA CCGCCCGTCGCTACTACCGA

A_Bcowani ACTATAGCCCACGAACGAGG AATTCCTGGTAAGGGCGAGT CACCAACTCGCCCTGACTAT GTCCCTGCCCTTTGTACACA CCGCCCGTCGCTACTACCGA

A_Ccowani nnnnnnnnnnnnnnnnnnnn nnnnnnnnnnnnnnnnnnnn nnnnnnnnnnnnnnnnnnnn nnnnnnnnnnnnnnnnnnnn nnnnnnnnnnnnnnnnnnnn

A_Dcowani ACTATAGCCCACGAACGAGG AATTCCTGGTAAGGGCGAGT CACCAACTCGCCCTGACTAT GTCCCTGCCCTTTGTACACA CCGCCCGTCGCTACTACCGA

A_Asangui nnnnnnnnnnnnnnnnnnnn nnnnnnnnnnnnnnnnnnnn nnnnnnnnnnnnnnnnnnnn nnnnnnnnnnnnnnnnnnnn nnnnnnnnnnnnnnnnnnnn

A_Bsangui nnnnnnnnnnnnnnnnnnnn nnnnnnnnnnnnnnnnnnnn nnnnnnnnnnnnnnnnnnnn nnnnnnnnnnnnnnnnnnnn nnnnnnnnnnnnnnnnnnnn

A_Csangui nnnnnnnnnnnnnnnnnnnn nnnnnnnnnnnnnnnnnnnn nnnnnnnnnnnnnnnnnnnn nnnnnnnnnnnnnnnnnnnn nnnnnnnnnnnnnnnnnnnn

A_Dsangui nnnnnnnnnnnnnnnnnnnn nnnnnnnnnnnnnnnnnnnn nnnnnnnnnnnnnnnnnnnn nnnnnnnnnnnnnnnnnnnn nnnnnnnnnnnnnnnnnnnn

Doratogon TTGAATGATTTAGTGAGGTC TTCGGACTGGGGCCCGGCGA GA----------CCTCCCTC ------GTGGG--------- -------GTCTCGCCGGTTC

Mad_maxAS TTGAATGGTTCAGTGAGGCC TATGGACAGGAGCCGGGCGA GACGGCCGGTGCCTTCCCCC TTAACCGGGTGGTGGTGCGC CTGGTCGGCCGAGCCTGCC-

Mad_maxBS TTGAATGGTTCAGTGAGGCC TATGGACAGGAGCCGGGCGA GACGGCCGGTGCCTTCCCCC TTAACCGGGTGGTGGTGCGC CTGGTCGGCCGAGCCTGCC-

S_simplex TTGAATGGTTCAGTGAGGCC TTTGGACAGGAGCCTGGCGA GG----------CGGCTCCT -----CGTGAGTT------- -------GCCGAGCCTGCT-

S_triareu TTGAATGGTTCAGTGAGGCC TTTGGACAGGAGCCTGGCGA GG----------CGGCTCTT -----CGTGAGTT------- -------GCCGAGCCTGCT-

C_AsemiSP nnnnnnnnnnnnnnnnnnnn nnnnnnnnnnnnnnnnnnnn nnnnnnnnnnnnnnnnnnnn nnnnnnnnnnnnnnnnnnnn nnnnnnnnnnnnnnnnnnnn

C_BsemiSP TTGAATGGTTCAGTGAGGCC TCCGGACGGGAGCCTGGCGA GA----------CCTCCCTC GCT------GGTC------- -------GTCGAGCCTGCT-

A_Avampyr TTGAATGGTTCAGTGAGGCC TCCGGACGGGAGCCTGGCGA GA----------CTTCCCCC TAAACCGGGGGTC------- -------GTCGAGCCTGCT-

A_Bvampyr TTGAATGGTTCAGTGAGGCC TCCGGACGGGAGCCTGGCGA GA----------CTTCCCCC ---ACCCGGGGTC------- -------GTCGAGCCTGCT-

A_Cvampyr TTGAATGGTTCAGTGAGGCC TCCGGACGGGAGCCTGGCGA GA----------CTTCCCCC ---ACCCGGGGTC------- -------GTCGAGCCTGCT-

A_Dvampyr TTGAATGGTTCAGTGAGGCC TCCGGACGGGAGCCTGGCGA GA----------CTTCCCCC TAAACCGGGGGTC------- -------GTCGAGCCTGCT-

A_Ainfern TTGAATGGTTCAGTGAGGCC TCCGGACGGGAGCCTGGCGA GA----------CTTCCCCC ---ACCCGGGGTC------- -------GTCGAGCCTGCT-

A_Binfern TTGAATGGTTCAGTGAGGCC TCCGGACGGGAGCCTGGCGA GA----------CTTCCCCC ---ACCCGGGGTC------- -------GTCGAGCCTGCT-

A_Cinfern TTGAATGGTTCAGTGAGGCC TCCGGACGGGAGCCTGGCGA GA----------CTTCCCCC ---ACCCGGGGTC------- -------GTCGAGCCTGCT-

A_Dinfern TTGAATGGTTCAGTGAGGCC TCCGGACGGGAGCCTGGCGA GA----------CTTCCCCC ---ACCCGGGGTC------- -------GTCGAGCCTGCT-

A_Einfern TTGAATGGTTCAGTGAGGCC TCCGGACGGGAGCCTGGCGA GA----------CTTCCCCC ---ACCCGGGGTC------- -------GTCGAGCCTGCT-

A_Acorall TTGAATGGTTCAGTGAGGCC TCCGGACGGGAGCCTGGCGA GA----------CTTCCCCC CAACAAGGGAGTC------- -------GTCGAGCCTGCT-

A_Bcorall TTGAATGGTTCAGTGAGGCC TCCGGACGGGAGCCTGGCGA GA----------CTTCCCCC CAACAAGGGAGTC------- -------GTCGAGCCTGCT-

A_nsp1229 TTGAATGGTTCAGTGAGGCC TCCGGACGGGAGCCTGGCGA GA----------CTTCCCCA ---ACCCGGGGTC------- -------GTCGAGCCTGCT-

A_nsp1396 TTGAATGGTTCAGTGAGGCC TCCGGACGGGAGCCTGGCGA GA----------CTTCCCCG CCA---CGGGGTC------- -------GTCGAGCCTGCT-

A_Aerythr nnnnnnnnnnnnnnnnnnnn nnnnnnnnnnnnnnnnnnnn nnnnnnnnnnnnnnnnnnnn nnnnnnnnnnnnnnnnnnnn nnnnnnnnnnnnnnnnnnnn

A_Berythr nnnnnnnnnnnnnnnnnnnn nnnnnnnnnnnnnnnnnnnn nnnnnnnnnnnnnnnnnnnn nnnnnnnnnnnnnnnnnnnn nnnnnnnnnnnnnnnnnnnn

A_AhovaSP TTGAATGGTTCAGTGAGGCC TCCGGACGGGAGCCTGGCGA GA----------CTTCCCCC ------CGGGGTC------- -------GTCGAGCCTGCT-

A_BhovaSP TTGAATGGTTCAGTGAGGCC TCCGGACGGGAGCCTGGCGA GA----------CTTCCCCC ------CGGGGTC------- -------GTCGAGCCTGCT-

A_ignipes TTGAATGGTTCAGTGAGGCC TCCGGACGGGAGCCTGGCGA GA----------CTTCCCCC TTG--CCGGGGTC------- -------GTCGAGCCTGCT-

A_Bcowani TTGAATGGTTCAGTGAGGCC TCCGGACGGGAGCCTGGCGA GA----------CTTCCCCC TTG--CCGGGGTC------- -------GTCGAGCCTGCT-

A_Ccowani nnnnnnnnnnnnnnnnnnnn nnnnnnnnnnnnnnnnnnnn nnnnnnnnnnnnnnnnnnnn nnnnnnnnnnnnnnnnnnnn nnnnnnnnnnnnnnnnnnnn

A_Dcowani TTGAATGGTTCAGTGAGGCC TCCGGACGGGAGCCTGGCGA GA----------CTTCCCCC TTG--CCGGGGTC------- -------GTCGAGCCTGCT-

A_Asangui nnnnnnnnnnnnnnnnnnnn nnnnnnnnnnnnnnnnnnnn nnnnnnnnnnnnnnnnnnnn nnnnnnnnnnnnnnnnnnnn nnnnnnnnnnnnnnnnnnnn

A_Bsangui nnnnnnnnnnnnnnnnnnnn nnnnnnnnnnnnnnnnnnnn nnnnnnnnnnnnnnnnnnnn nnnnnnnnnnnnnnnnnnnn nnnnnnnnnnnnnnnnnnnn

A_Csangui nnnnnnnnnnnnnnnnnnnn nnnnnnnnnnnnnnnnnnnn nnnnnnnnnnnnnnnnnnnn nnnnnnnnnnnnnnnnnnnn nnnnnnnnnnnnnnnnnnnn

A_Dsangui nnnnnnnnnnnnnnnnnnnn nnnnnnnnnnnnnnnnnnnn nnnnnnnnnnnnnnnnnnnn nnnnnnnnnnnnnnnnnnnn nnnnnnnnnnnnnnnnnnnn

Doratogon GTCTCGGAAAGATGACCGAA CTTGACCATTTAGAGGAAGT AAAAGTCGTAACAAGGTTTC C--AATAAATGTTGATATAA GATTGGATCTCCTCCCCCTG

Mad_maxAS GCACCTGGAAGCTGACCGAA CTTGACCACTTAGAGGAAGT AAAAGTCGTAACAAGGTAAC CGTAATAAATGTTGGTATAA AATAGGGTCTCCACCNCCAG

Mad_maxBS GCACCTGGAAGCTGACCGAA CTTGACCACTTAGAGGAAGT AAAAGTCGTAACAAGGTAAC CGTAATAAATGTTGGTATAA AATAGGGTCTCCACCNCCAG

S_simplex GCTTCTGGAAGCTGACCGAA CTTGACCATTTAGAGGAAGT AAAAGTCGTAACAAGGTAAC CGTAAGAGATGTTGATATAA AATAGGGTCACCACCTCCTG

S_triareu GCTTCTGAAAGCTGACCGAA CTTGACCATTTAGAGGAAGT AAAAGTCGTAACAAGGTAAC CGTAATAAGTGTTGGTATAA AATAGGGTCTCCTCCACCTG

C_AsemiSP nnnnnnnnnnnnnnnnnnnn nnnnnnnnnnnnnnnnnnnn nnnnnnnnnnnnnnnnnnnn nnnAAGAGATGTTGATATAA AATAGGATCTCCTCCTCCTG

C_BsemiSP GCTCCCGGAAGCTGACCGAA CTTGACCATTTAGAGGAAGT AAAAGTCGTAACAAGGTAAC CGTAAGAGATGTTGATATAA AAAAGGATCTCCTCCTCCGG

A_Avampyr GTTCCCGGAAGCTGACCGAA CTTGACCATTTAGAGGAAGT AAAAGTCGTAACAAGGTAAC CGTnnnnnnnnnnnnnnnnn nnnnnnnnnnnnnnnnnnnn

A_Bvampyr GTTCCCGGAAGCTGACCGAA CTTGACCA-TTAGAGGAAGT AAAAGTCGTAACAAGGTAAC CGTAATAAATGTTGATAAAG AATAGGGTCACCTCCTCCAG

A_Cvampyr GTTCCCGGAAGCTGACCGAA CTTGACCATTTAGAGGAAGT AAAAGTCGTAACAAGGTAAC CGTAATAAATGTTGATAAAG AATAGGATCTCCTCCTCCAG

A_Dvampyr GTTCCCGGAAGCTGACCGAA CTTGACCATTTAGAGGAAGT AAAAGTCGTAACAAGGTAAC CGTnnnnnnnnnnnnnnnnn nnnnnnnnnnnnnnnnnnnn

A_Ainfern GTTCCCGGAAGCTGACCGAA CTTGACCATTTAGAGGAAGT AAAAGTCGTAACAAGGTAAC CGTAATAAATGTTGATAAAG AATAGGGTCTCCTCCCCCTG

A_Binfern GTTCCCGGAAGCTGACCGAA CTTGACCATTTAGAGGAAGT AAAAGTCGTAACAAGGTAAC CGTAATAAATGTTGATAAAG AATAGGGTCTCCTCCCCCTG

A_Cinfern GTTCCCGGAAGCTGACCGAA CTTGACCATTTAGAGGAAGT AAAAGTCGTAACAAGGTAAC CGTAATAAATGTTGATAGAG AATAGGGTCTCCTCCTCCTG

A_Dinfern GTTCCCGGAAGCTGACCGAA CTTGACCATTTAGAGGAAGT AAAAGTCGTAACAAGGTAAC CGTnnnnnnnnnnnnnnnnn nnnnnnnnnnnnnnnnnnnn

A_Einfern GTTCCCGGAAGCTGACCGAA CTTGACCATTTAGAGGAAGT AAAAGTCGTAACAAGGTAAC CGTAATAAATGTTGATAGAG AATAGGGTCTCCTCCCCCTG

A_Acorall GTTCCCGGAAGCTGACCGAA CTTGACCATTTAGAGGAAGT AAAAGTCGTAACAAGGTAAC CGTAATAAATGATGATAAAG AATAGGATCTCCACCTCCAG

A_Bcorall GTTCCCGGAAGCTGACCGAA CTTGACCATTTAGAGGAAGT AAAAGTCGTAACAAGGTAAC CGTATTAAATGATGATAAAG AATAGGATCTCCACCTCCAG

A_nsp1229 GCTCCCGGAAGCTGACCGAA CTTGACCATTTAGAGGAAGT AAAAGTCGTAACAAGGTAAC CGTAATAAATGTTGATAAAG AATAGGATCTCCTCCTCCGG

A_nsp1396 GCTCCCGGAAGCTGACCGAA CTTGACCATTTAGAGGAAGT AAAAGTCGTAACAAGGTAAC CGTAATAAATGTTGATAGAG AATAGGATCTCCCCCGCCAG

A_Aerythr nnnnnnnnnnnnnnnnnnnn nnnnnnnnnnnnnnnnnnnn nnnnnnnnnnnnnnnnnnnn nnnAAGAGATGTTGATAAAG AATAGGATCTCCCCCTCCTG

A_Berythr nnnnnnnnnnnnnnnnnnnn nnnnnnnnnnnnnnnnnnnn nnnnnnnnnnnnnnnnnnnn nnnAAGAGATGTTGATAAAG AATAGGATCTCCTCCTCCTG

A_AhovaSP GCACCCGAAAGCTGACCGAA CTTGACCATTTAGAGGAAGT AAAAGTCGTAACAAGGTAAC CGTAATAAATGTTGATAGAG AATAGGATCCCCTCCTCCAG

A_BhovaSP GCACCCGAAAGCTGACCGAA CTTGACCATTTAGAGGAAGT AAAAGTCGTAACAAGGTAAC CGTAATAAATGTTGATAGAG AATAGGATCCCCTCCTCCAG

A_ignipes GCACCCGAAAGCTGACCGAA CTTGACCATTTAGAGGAAGT AAAAGTCGTAACAAGGTAAC CGT----------------- --------------------

A_Bcowani GCTCCCGGAAGCTGACCGAA CTTGACCATTTAGAGGAAGT AAAAGTCGTAACAAGGTAAC CGTAATAAGTGTTGATATAA AATAGGATCTCCACCTCCAG

A_Ccowani nnnnnnnnnnnnnnnnnnnn nnnnnnnnnnnnnnnnnnnn nnnnnnnnnnnnnnnnnnnn nnnAATAAGTGTTGATATAA AATAGGATCTCCACCTCCAG

A_Dcowani GCTCCCGGAAGCTGACCGAA CTTGACCATTTAGAGGAAGT AAAAGTCGTAACAAGGTAAC CGTAATAAGTGTTGATATAA AATAGGATCTCCACCTCCAG

A_Asangui nnnnnnnnnnnnnnnnnnnn nnnnnnnnnnnnnnnnnnnn nnnnnnnnnnnnnnnnnnnn nnnAATAAGTGTTGATATAA AATAGGATCTCCACCTCCAG

A_Bsangui nnnnnnnnnnnnnnnnnnnn nnnnnnnnnnnnnnnnnnnn nnnnnnnnnnnnnnnnnnnn nnnAATAAGTGTTGATATAA AATAGGATCTCCACCTCCAG

A_Csangui nnnnnnnnnnnnnnnnnnnn nnnnnnnnnnnnnnnnnnnn nnnnnnnnnnnnnnnnnnnn nnnAATAAGTGTTGATATAA AATAGGATCTCCACCTCCAG

A_Dsangui nnnnnnnnnnnnnnnnnnnn nnnnnnnnnnnnnnnnnnnn nnnnnnnnnnnnnnnnnnnn nnnAATAAGTGTTGATATAA AATAGGATCTCCACCTCCAG

Doratogon CAGGGTCGAAAAAGGAGGTA TTAAAATTACGATCTGTTAA TAGTATTGTAATTGCTCCTG CTAAGACTGGGAGGGAAAGA AGAAGTAAAATAGCTGTAAT

Mad_maxAS AAGGGTCAAAGAATGATGTA TTAAAGTTTCGGTCAGTGAG AAGTATTGTGATAGCACCTG CTAGAACTGGGAGGGAAAGA AGAAGAAGAATTGCAGTGAT

Mad_maxBS AAGGGTCAAAGAATGATGTA TTAAAGTTTCGGTCAGTGAG AAGTATTGTGATAGCACCTG CTAGAACTGGGAGGGAAAGA AGAAGAAGAATTGCAGTGAT

S_simplex CCGGATCAAAAAATGAAGTA TTGAAGTTGCGGTCAGTTAG TAGCATTGTAATAGCTCCTG CTAGCACAGGGAGGGAGAGT AGCAGTAGAATTGTCGTGAT

S_triareu CGGGGTCAAAAAATGAAGTA TTGAAATTACGGTCTGTTAA AAGTATGGTAATAGCGCCTG CTAAAACGGGTAAAGATAAG AGTAGGAGGATAGCAGTAAG

C_AsemiSP AAGGGTCAAAGAAGGAAGTG TTAAAGTTTCGATCAGTTAA TAATATTGTAATGGCACCTG CTAATACCGGTAAAGAAAGT AACAATAAGATTGCAGTAAT

C_BsemiSP AAGGGTCAAAGAAGGAAGTG TTAAAGTTTCGATCAGTTAA TAATATTGTAATGGCACCTG CTAATACCGGTAAAGAAAGT AACAATAAGATTGCAGTAAT

A_Avampyr nnnnnnnnnnnnnnnnnnnn nnnnnnnnnnnnnnnnnnnn nnnnnnnnnnnnnnnnnnnn nnnnnnnnnnnnnnnnnnnn nnnnnnnnnnnnnnnnnnnn

A_Bvampyr ATGGATCAAAAAAGGAAGTA TTAAAATTNCGATCAGTTAA TAATATAGTAATAGCTCCTG CTAATACTGGTAAAGATAGA AGTAATAGAATTGCGGNAAT

A_Cvampyr ATGGATCAAAAAAGGAGGTA TTAAAATTACGATCAGTTAA TAATATAGTAATAGCTCCTG CTAATACTGGTAAAGATAGA AGTAATAAAATTGCGGTATT

A_Dvampyr nnnnnnnnnnnnnnnnnnnn nnnnnnnnnnnnnnnnnnnn nnnnnnnnnnnnnnnnnnnn nnnnnnnnnnnnnnnnnnnn nnnnnnnnnnnnnnnnnnnn

A_Ainfern ACGGATCAAAAAAGGAAGTA TTAAAATTACGATCAGTTAA TAATATAGTAATAGCTCCTG CTAATACTGGTAATGATAGG AGAAGTAAAATTGCAGTAAT

A_Binfern ATGGATCAAAAAAGGAAGTA TTAAAATTACGATCAGTTAA TAATATAGTAATAGCTCCTG CTAATACTGGTAATGATAGG AGAAGTAAAATTGCAGTAAT

A_Cinfern AGGGATCAAAAAAGGAAGTA TTAAAGTTACGATCAGTTAA TAGTATAGTAATAGCTCCTG CTAATACTGGTAATGATAGG AGGAGTAAAATTGCAGTAAT

A_Dinfern nnnnnnnnnnnnnnnnnnnn nnnnnnnnnnnnnnnnnnnn nnnnnnnnnnnnnnnnnnnn nnnnnnnnnnnnnnnnnnnn nnnnnnnnnnnnnnnnnnnn

A_Einfern AGGGATCAAAAAAGGAAGTA TTAAAATTACGATCAGTTAA TAGTATAGTAATAGCTCCTG CTAATACTGGTAATGAGAGG AGGAGTAAAATTGCAGTAAT

A_Acorall AAAGATTGAAGAAGGATGTA TTTAAGTTGCGATCAGTTAA TAATATAGTAATAGCCCCCG CTAGTACTGGTAAAGATAAA AGTAATAAAATTGCAGTGAT

A_Bcorall AAAGATTGAAGAAGGATGTA TTTAAGTTGCGATCAGTTAA TAATATAGTAATAGCCCCCG CTAGTACTGGTAAAGATAAA AGTAATAAAATTGCAGTGAT

A_nsp1229 AAGGATCAAAAAATGAAGTA TTAAAGTTTCGATCAGTTAA TAATATGGTAATAGCTCCTG CTAATACTGGTAAAGAGAGA AGCAATAAAATTGCAGTAAT

A_nsp1396 AAGGGTCAAAGAAAGAAGTA TTAAAGTTACGATCAGTTAA TAATATAGTAATAGCTCCTG CTAATACTGGTAAGGAGAGA AGTAATAAAATTGCAGTAAT

A_Aerythr AAGGATCAAAAAAAGATGTA TTAAAATTTCGATCAGTTAA TAGTATGGTAATAGCTCCGG CTAATACAGGTAATGATAGG AGTAGTAGGATTGCAGTAAT

A_Berythr AGGGGTCAAAAAAAGATGTA TTAAAATTTCGATCAGTCAA TAATATAGTAATGGCTCCGG CTAATACAGGCAATGATAAA AGTAATAAGATTGCAGTAAT

A_AhovaSP AGGGATCAAAAAAAGAGGTA TTAAAATTACGATCAGTTAA TAATATAGTAATAGCTCCAG CTAATACAGGTAATGATAAA AGTAATAAGATTGCAGTAAT

A_BhovaSP AGGGATCAAAAAATGAGGTA TTAAAATTACGATCAGTTAA TAGTATGGTAATAGCCCCGG CTAATACAGGTAATGATAAA AGTAATAAAATTGCAGTAAT

A_ignipes -----------------GTG TTGAAGTTAcGATCATTTAT TAATATGGTAATAGCCCCAG CTAAGACAGGTAATGATAAA AGTAATAAAATTGCAGTAAT

A_Bcowani AAGGATCAAAGAAGGATGTA TTAAAATTACGATCAGTCAG TAATATAGTAATAGCTCCAG CTAAGACAGGTAATGATAAA AGTAATAAAATTGCAGTGAT

A_Ccowani AAGGATCAAAGAAGGACGTA TTAAAATTACGATCAGTCAG TAATATAGTAATAGCTCCAG CTAAGACAGGCAATGATAAA AGTAATAAAATTGCAGTGAT

A_Dcowani AAGGATCAAAGAAGGACGTA TTAAAATTACGATCAGTCAG TAATATAGTAATAGCTCCAG CTAAGACAGGCAATGATAAA AGTAATAAAATTGCAGTGAT

A_Asangui AGGGATCAAAGAATGAGGTA TTAAAATTACGATCAGTTAA TAGTATAGTAATAGCTCCGG CTAGAACAGGTAATGATAGG AGTAATAAAATTGCAGTAAT

A_Bsangui AGGGATCAAAGAATGAGGTA TTAAAATTACGATCAGTTAA TAGTATAGTAATAGCTCCGG CTAGAACAGGTAATGATAGG AGTAATAAAATTGCAGTAAT

A_Csangui AGGGGTCAAAGAATGAGGTA TTAAAATTACGATCAGTTAA TAGTATAGTAATAGCTCCGG CTAGAACAGGTAATGATAGG AGTAATAAAATTGCAGTAAT

A_Dsangui AGGGATCAAAGAATGAGGTA TTAAAATTACGATCAGTTAA TAGTATAGTAATAGCTCCGG CTAGAACAGGTAATGATAGG AGTAATAAAATTGCAGTAAT

Doratogon TTTTACTGCTCATACAAACA AAGGTATTTGTTCAAATAGT ATACCTGCTGTCCGTATATT AATAATTGTAGTAATAAAAT TAATGGCTCCTAAAATTGAA

Mad_maxAS TTTTACGGCTCAGACAAATA AAGGTATTTGTTCAAATAAT ATTCCATTTGAGCGTATATT AATAATTGTGGTAATAAAGT TGATTGCACCTAGAATAGAT

Mad_maxBS TTTTACGGCTCAGACAAATA AAGGTATTTGTTCAAATAAT ATTCCATTTGAGCGTATATT AATAATTGTGGTAATAAAGT TGATTGCACCTAGAATAGAT

S_simplex TATAATTGAGCACACAAATA ATGGAACACGTTCAAGCAGT ATACCTCTTGATCGTATGTT AATAATAGTTGTAATAAAGT TAATGGCCCCTAAGATGGAT

S_triareu TTTTACAGCTCAAACAAATA AGGGCATTTGTTCAAATAAC ATGCCATTAGATCGTATATT AATGATTGTTGTAATAAAAT TAATGGCTCCTAAAATTGAG

C_AsemiSP TTTAACGGCTCATACAAATA AAGGTATTTGTTCAAATAAT ATACCATTAGATCGTATATT AATGATGGTAGTAATAAAAT TAATTGCTCCTAGAATTGAA

C_BsemiSP TTTAACGGCTCATACAAATA AAGGTATTTGTTCAAATAAT ATACCATTAGATCGTATATT AATGATGGTAGTAATAAAAT TAATTGCTCCTAGAATTGAA

A_Avampyr nnnnnnnnnnnnnnnnnnnn nnnnnnnnnnnnnnnnnnnn nnnnnnnnnnnnnnnnnnnn nnnnnnnnnnnnnnnnnnnn nnnnnnnnnnnnnnnnnnnn

A_Bvampyr TTTAACAGCTCATACAAATA AAGGTATTTGTTCAAATAAT ATACCATTAGATCGTATGTT AATAATAGTAGTAATAAAAT TAATAGCACCTAGAATTGAA

A_Cvampyr TTTAACAGCTCATACAAATA AAGGTATTTGTTCAAATAAT ATGCCATTAGATCGTATGTT AATAATAGTAGTAATAAAAT TAATAGCACCTAGAATTGAA

A_Dvampyr nnnnnnnnnnnnnnnnnnnn nnnnnnnnnnnnnnnnnnnn nnnnnnnnnnnnnnnnnnnn nnnnnnnnnnnnnnnnnnnn nnnnnnnnnnnnnnnnnnnn

A_Ainfern TTTAACAGCTCATACAAATA AAGGTATTTGTTCAAATAAT ATACCATTAGATCGTATATT AATGATGGTAGTAATAAAAT TAATGGCTCCTAAAATTGAG

A_Binfern TTTAACAGCTCATACAAATA AAGGTATTTGTTCAAATAAT ATACCATTAGATCGTATATT AATGATGGTAGTAATAAAAT TAATGGCTCCTAAAATTGAG

A_Cinfern TTTAACAGCTCATACAAATA AAGGTATTTGTTCAAATAAT ATACCATTAGATCGCATATT AATGATGGTAGTGATAAAAT TAATAGCCCCTAAAATTGAA

A_Dinfern nnnnnnnnnnnnnnnnnnnn nnnnnnnnnnnnnnnnnnnn nnnnnnnnnnnnnnnnnnnn nnnnnnnnnnnnnnnnnnnn nnnnnnnnnnnnnnnnnnnn

A_Einfern TTTAACAGCTCATACAAATA AAGGTATTTGTTCAAATAAT ATACCATTAGATCGTATATT AATGATGGTAGTGATAAAAT TAATAGCTCCTAAAATTGAA

A_Acorall TTTAACAGCCCATACAAATA ATGGTATTTGTTAAAATAAT ATACCATTAGATCGTATATT GATAATAGTAGTAAAAAAAT TAATAGCACCTTAAATGGAT

A_Bcorall TTTAACAGCCCATACAAATA ATGGTATTTGTTAAAATAAT ATACCATTAGATCGTATATT GATAATAGTAGTAAAAAAAT TAATAGCACCTTAAATGGAT

A_nsp1229 TTTAACGGCTCATACAAACA GAGGTATTTGTTCAAATAAT ATTCCGTTAGATCGTATGTT AACAATGGTAGTAATAAAAT TAATAGCACCTAAAATTGAA

A_nsp1396 TTTAACGGCTCATACAAATA AAGGTATTTGTTCAAATAAT ATTCCATTAGATCGCATATT AATAATGGTAGTAATAAAAT TAATGGCTCCTAAAATTGAG

A_Aerythr TTTTACTGCTCATACAAATA GAGGCATTTGTTCAAATAGT ATACCATTAGAGCGTATATT AATGATGGTAGTAATGAAAT TAATAGCTCCTAAAATTGAA

A_Berythr TTTTACTGCCCATACAAATA GAGGTATTTGTTCAAATAAT ATTCCATTAGAACGTATATT AATAATAGTGGTAATAAAAT TGATAGCTCCTAAAATTGAA

A_AhovaSP TTTTACTGCTCACACAAATA GAGGTATTTGTTCAAATAGC ATACCATTGGATCGTATATT AATGATGGTAGTAATAAAAT TGATTGCTCCTAAAATTGAA

A_BhovaSP CTTTACTGCTCATACAAATA GAGGTATTTGTTCAAATAAT ATACCATTAGACCGTATGTT AATGATAGTAGTAATAAAAT TGATTGCTCCTAGAATTGAA

A_ignipes TTTTACTGCTCATACAAATA AAGGTATTTGTTCAAACAAT ATACCATTAGATCGTATGTT GACAATAGTAGTAATAAAAT TAATTGCCCCTAAAATTGAA

A_Bcowani TTTAACTGCTCACACAAATA AAGGTATTTGCTCAAATAGC ATCCCGTTAGATCGTATATT AATAATTGTGGTAATAAAAT TAATTGCCCCTAAAATGGAG

A_Ccowani TTTAACTGCTCACACAAATA AAGGTATTTGCTCAAATAGC ATCCCGTTAGATCGTATATT AATAATTGTGGTAATAAAAT TAATTGCCCCTAAAATGGAG

A_Dcowani TTTAACTGCTCACACAAATA AAGGTATTTGCTCGAATAGC ATCCCGTTAGATCGTATATT AATAATTGTGGTAATAAAAT TAATTGCCCCTAAAATGGAG

A_Asangui TTTAACTGCTCATACAAATA AAGGTATTTGTTCAAATAAC ATTCCATTAGATCGTATATT AATAATTGTAGTAATAAAGT TAATGGCACCTAAAATTGAA

A_Bsangui TTTAACTGCTCATACAAATA AAGGTATTTGTTCAAATAAC ATTCCATTAGATCGTATATT AATAATTGTAGTAATAAAGT TAATGGCACCTAAAATTGAA

A_Csangui TTTAACTGCTCATACAAATA AAGGTATTTGTTCAAATAAC ATTCCATTAGATCGTATATT AATAATTGTAGTAATAAAGT TAATGGCACCTAAAATTGAA

A_Dsangui TTTAACTGCTCATACAAATA AAGGTATTTGTTCAAATAAC ATTCCATTAGATCGTATATT AATAATTGTAGTAATAAAGT TAATGGCACCTAAAATTGAA

Doratogon GAGGCCCCTGCAAGATGTAA TGAAAAAATAGCTATATCTA CGGAAGGACCTGCATGAGCT AAGGTGGATGCTAAAGGAGG ATAGACTGTTCATCCTGTTC

Mad_maxAS GAAGCTCCTGCTAGATGTAA TGAGAAAATAGCTATATCTA CGGAGGGTCCAGCATGGGCT AGGTTAGAGGCTAGAGGGGG ATATACTGTTCATCCAGTAC

Mad_maxBS GAAGCTCCTGCTAGATGTAA TGAGAAAATAGCTATATCTA CGGAGGGTCCAGCATGGGCT AGGTTAGAGGCTAGAGGGGG ATATACTGTTCATCCAGTAC

S_simplex GAAACACCTGCTAAGTGTAA GGAGAAAATTGCTATATCTA CGGCAGGGCCGGCATGGGCA ATTCTAGCCGCAAGCGGGGG GTATACAGTTCAACCTGTTC

S_triareu GAGGCTCCTGCCAAGTGTAG GGAAAAGATCGCTATATCTA CGGAGGGCCCACTGTGGGCT AAGTTGGATGCTAAAGGGGG GTACACTGTCCATCCTGTAC

C_AsemiSP GAAGCACCTGCTAGGTGTAA TGAAAAGATTGCTATATCAA CAGAAGGACCAGAGTGGGCT AGATTTGAGGCTAAAGGGGG ATATACTGTTCAGCCTGTAC

C_BsemiSP GAAGCCCCTGCTAGGTGTAA TGAAAAGATTGCTATATCAA CAGAAGGACCAGAGTGGGCT AGATTTGAGGCTAAAGGGGG ATATACTGTTCAGCCTGTAC

A_Avampyr nnnnnnnnnnnnnnnnnnnn nnnnnnnnnnnnnnnnnnnn nnnnnnnnnnnnnnnnnnnn nnnnnnnnnnnnnnnnnnnn nnnnnnnnnnnnnnnnnnnn

A_Bvampyr GAGGCACCTGCTAGATGTAA AGAGAAAATTGCTATATCTA CAGAGGGGCCTGAATGAGCA AGATTTGAGGCTAAAGGAGG ATAGACAGTCCATCCTGTTC

A_Cvampyr GAGGCACCTGCTAGATGTAA AGAGAAAATTGCTATATCTA CAGAAGGGCCTGAATGAGCA AGATTTGAGGCTAAAGGAGG ATAAACAGTTCATCCTGTTC

A_Dvampyr nnnnnnnnnnnnnnnnnnnn nnnnnnnnnnnnnnnnnnnn nnnnnnnnnnnnnnnnnnnn nnnnnnnnnnnnnnnnnnnn nnnnnnnnnnnnnnnnnnnn

A_Ainfern GAAGCACCTGCTAGATGTAG AGAAAAAATTGCTATGTCTA CAGAGGGACCTGAATGAGCA AGATTTGAAGCTAGAGGGGG ATAAACAGTTCATCCTGTTC

A_Binfern GAAGCACCTGCTAGATGTAG AGAAAAAATTGCTATGTCTA CAGAGGGACCTGAATGAGCA AGATTTGAAGCTAGAGGGGG ATAAACAGTTCATCCTGTTC

A_Cinfern GAGGCACCTGCTAGATGTAG AGAAAAAATTGCTATGTCTA CAGAGGGACCTGAATGGGCA AGATTTGAAGCTAGAGGAGG GTAAACAGTTCATCCTGTTC

A_Dinfern nnnnnnnnnnnnnnnnnnnn nnnnnnnnnnnnnnnnnnnn nnnnnnnnnnnnnnnnnnnn nnnnnnnnnnnnnnnnnnnn nnnnnnnnnnnnnnnnnnnn

A_Einfern GAGGCACCTGCTAGATGTAG AGAAAAAATTGCTATGTCTA CAGAGGGACCTGAATGAGCA AGATTTGAAGCTAGAGGGGG ATAAACAGTTCATCCTGTTC

A_Acorall GAGGCACCTGCTAGATGTAA AGAAAAAATTGCTATGTCTA CNGAGGAACCTGAGTGGGGA AGATTTGACGCTAAAGGAGG ATAAAGTGTTCATCCTGTTC

A_Bcorall GAGGCACCTGCTAGATGTAA AGAAAAAATTGCTATGTCTA CAGAGGAACCTGAGTGGGGA AGATTTGACGCTAAAGGAGG ATAAAGTGTTCATCCTGTTC

A_nsp1229 GAAGCTCCGGCTAGATGTAA AGAAAAAATTGCTATATCTA CAGAAGGGCCTGAGTGAGCA AAATTTGAGGCCAAGGGAGG ATAAACAGTTCATCCTGTTC

A_nsp1396 GAAGCACCTGCTAAGTGTAA GGAAAAAATTGCTATGTCTA CAGAAGGTCCTGAATGGGCA AGATTTGAAGCTAAAGGAGG ATAAACAGTTCATCCTGTTC

A_Aerythr GAGGCTCCTGCTAAGTGTAA GGAAAAAATAGCTATATCTA CAGAAGGTCCTGAGTGAGCT AAATTAGAAGCTAAAGGAGG ATAAACAGTCCAGCCTGTCC

A_Berythr GAAGCTCCTGCTAAGTGTAA GGAAAAAATTGCTATATCTA CAGAAGGGCCTGAGTGAGCT AAATTAGAGGCTAAAGGAGG ATAGACAGTCCATCCTGTCC

A_AhovaSP GAAGCTCCTGCTAAATGTAA GGAAAAAATTGCTATATCTA CTGAAGGTCCTGAATGGGCT AAGTTAGAAGCTAAAGGGGG GTAGACTGTTCATCCAGTTC

A_BhovaSP GAAGCTCCTGCTAGATGTAA AGAAAAAATTGCTATATCTA CTGAGGGTCCTGAGTGAGCT AAATTAGAGGCTAAAGGGGG ATATACTGTTCATCCAGTTC

A_ignipes GAAGCTCCTGCCAAATGTAA AGAAAAAATTGCTATGTCTA CTGAGGGSCATGAGTGGGCT AGGTTAGAAGATARAGGGGG ATAAACCGTCCATCCAGTTC

A_Bcowani GAAGCTCCTGCTAAATGTAG TGAAAAAATTGCTATGTCTA CAGAAGGACCTGAGTGGGCT AGATTAGAAGCTAAAGGAGG ATAAACAGTTCATCCTGTCC

A_Ccowani GAAGCTCCTGCTAAATGTAG TGAAAAAATTGCTATGTCTA CAGAAGGACCTGAGTGGGCT AGATTAGAAGCTAAAGGAGG ATAAACAGTTCATCCTGTCC

A_Dcowani GAAGCTCCTGCTAAATGTAG TGAAAAAATTGCTATGTCTA CAGAAGGACCTGAGTGGGCT AGATTAGAAGCTAAAGGAGG ATAAACAGTTCATCCTGTCC

A_Asangui GAGGCTCCTGCTAAATGTAA AGAAAAAATTGCTATGTCTA CGGAAGGACCTGAGTGAGCT ATATTGGAAGCTAAAGGGGG ATAAACAGTTCACCCTGTTC

A_Bsangui GAGGCTCCTGCTAAATGTAA AGAAAAAATTGCTATGTCTA CGGAAGGACCTGAGTGAGCT ATATTGGAAGCTAAAGGGGG ATAAACAGTTCACCCTGTTC

A_Csangui GAGGCTCCTGCTAAATGTAA AGAAAAAATTGCTATGTCTA CGGAAGGACCTGAGTGAGCT ATATTGGAAGCTAAAGGGGG ATAAACAGTTCACCCTGTTC

A_Dsangui GAGGCTCCTGCTAAATGTAA AGAAAAAATTGCTATGTCTA CGGAAGGACCTGAGTGAGCT ATATTGGAAGCTAAAGGGGG ATAAACAGTTCACCCTGTTC

Doratogon CAGCCCCTTTTTCTACTGCA GATGAAGCTAGTAATAAAAA TAAAGCTGGTGGAAGTAGTC AGAATCTTAGATTGTTTATT CGTGGAAAGGCTATATCAGG

Mad_maxAS CAGCTCCCTTGTCAACTAGT GAGGAAGAGATTAAAAGGAA GAATGCAGGAGGAAGAAGCC AAAAGCTTATATTGTTTAGA CGTGGGAAGGCTATATCTGG

Mad_maxBS CAGCTCCCTTGTCAACTAGT GAGGAAGAGATTAAAAGGAA GAATGCAGGAGGAAGAAGCC AAAAGCTTATATTGTTTAGA CGTGGGAAGGCTATATCTGG

S_simplex CTGGGCCATCTTCGACGATA GCGGATGAGATTAAGAGGAT AAGAGCTGGGGGGAGCAGTC AGAAACTAAGATTATTGAGT CGTGGGAAGGCTATGTCTGG

S_triareu CAGCGCCTTTATCTACAAGA GAGGAAGAAATCAGTAGAAA AAATGCAGGTGGTAATAATC AGAATCTTATATTATTTATA CGAGGGAAAGCTATGTCGGG

C_AsemiSP CGGCTCCTTTATCTACAAGA GCAGAAGAAATTAATAGGAA AAATGCAGGGGGGAGTAATC AAAATCTCATGTTATTTATT CGAGGAAAGGCTATGTCTGG

C_BsemiSP CGGCTCCTTTATCTACAAGA GCAGAAGAAATTAATAGGAA AAATGCAGGGGGGAGTAATC AAAATCTTATGTTATTTATT CGAGGAAAGGCTATGTCTGG

A_Avampyr nnnnnnnnnnnnnnnnnnnn nnnnnnnnnnnnnnnnnnnn nnnnnnnnnnnnnnnnnnnn nnnnnnnnnnnnnnnnnnnn nnnnnnnnnnnnnnnnnnnn

A_Bvampyr CGGCTCCTTTATCTACAAGG GAGGAAGAGATAAGTAAGAA GAAAGCAGGAGGTAATAATC AAAATCTTATGTTATTTATT CGAGGAAAGGCTATATCAGG

A_Cvampyr CAGCCCCTTTATCTACAAGG GAGGAAGAGATAAGTAAGAA GAAAGCAGGAGGTAATAATC AAAATCTCATGTTATTTATT CGAGGTAAGGCTATATCAGG

A_Dvampyr nnnnnnnnnnnnnnnnnnnn nnnnnnnnnnnnnnnnnnnn nnnnnnnnnnnnnnnnnnnn nnnnnnnnnnnnnnnnnnnn nnnnnnnnnnnnnnnnnnnn

A_Ainfern CAACTCCTTTATCTACAAGA GAAGAAGAAATGAGCAGGAA AAAAGCGGGAGGTAATAATC AAAATCTTATATTATTTATT CGAGGAAAAGCTATATCAGG

A_Binfern CAACTCCTTTATCTACAAGA GAAGAAGAAATGAGCAGGAA AAAAGCGGGAGGTAATAATC AAAATCTTATATTATTTATT CGAGGAAAAGCTATATCAGG

A_Cinfern CGGCTCCTTTATCTACAAGA GAAGAAGAAATGAGCAAGAA AAAAGCAGGAGGTAATAGTC AAAATCTTATATTATTTATT CGAGGAAAAGCTATATCAGG

A_Dinfern nnnnnnnnnnnnnnnnnnnn nnnnnnnnnnnnnnnnnnnn nnnnnnnnnnnnnnnnnnnn nnnnnnnnnnnnnnnnnnnn nnnnnnnnnnnnnnnnnnnn

A_Einfern CTGCTCCTTTATCTACAAGA GAAGAAGAAATGAGCAGGAA AAAAGCAGGAGGTAATAGTC AAAATCTTATATTATTTATT CGAGGAAAAGCTATATCAGG

A_Acorall CAGCCCCTTTATCTACAAGT GAGGAAGAAATAAGTAAGAA AAAAGCTGGGGGTAATAATC AAAATTTTATATTGTTTATT CGAGGGAAAGCTATATCAGG

A_Bcorall CAGCCCCTTTATCTACAAGT GAGGAAGAAATAAGTAAGAA AAAAGCTGGGGGTAATAATC AAAATTTTATATTGTTTATT CGAGGGAAAGCTATATCAGG

A_nsp1229 CTGCTCCTTTATCTACAAGA GACGAAGAAATTAATAAAAA AAAAGCAGGGGGTAACAATC AAAATCTTATATTATTTATT CGAGGAAAGGCTATGTCAGG

A_nsp1396 CAGCTCCTTTGTCTACGAGG GATGAGGAAATTAATAAGAA AAAGGCAGGAGGTAATAATC AAAATCTTATATTATTTATT CGAGGGAAGGCTATATCAGG

A_Aerythr CAGCACCTTTGTCTACAAGA GAAGAAGAAATTAATAAAAA GAATGCAGGAGGTAATAATC AAAATCTTATATTATTTATT CGAGGAAAAGCTATATCAGG

A_Berythr CAACACCTTTATCAACAAGA GAAGAAGAAATTAATAAAAA GAATGCAGGAGGTAGTAGCC AAAATCTTATATTATTTATT CGAGGAAAGGCTATATCAGG

A_AhovaSP CAGCACCTTTATCTACAAGA GAGGAGGAGATTAATAAAAA GAATGCAGGAGGGAGTAACC AGAATCTTATATTATTTATT CGAGGAAAAGCTATGTCGGG

A_BhovaSP CAGCACCTTTATCTACAAGA GATGAAGAGATTAATAGGAA AAATGCAGGAGGAAGTAACC AAAATCTTATGTTATTTATT CGAGGGAAGGCTATATCTGG

A_ignipes CAGCACCTTTGTCTACAAGA GAAGAAGAAATTAATAAAAG AAATGCAGGAGGAAGTAACC AGAATCTTATAATGTTTATT cGAGGGAAGGCTATATCAGG

A_Bcowani CGGCTCCTTTATCTACTAGT GAAGAGGAAATTAGTAAAAA GAATGCAGGGGGAAGTAGTC AAAAGCTTATATTATTTATT CGAGGGAATGCTATATCAGG

A_Ccowani CGGCTCCTTTATCTACTAGT GAAGAGGAAATTAGTAAAAA GAATGCAGGGGGAAGTAGTC AAAAGCTTATATTATTTATT CGAGGGAATGCTATATCAGG

A_Dcowani CGGCTCCTTTATCTACTAGT GAAGAGGAAATTAGTAAAAA GAATGCAGGGGGAAGTAGTC AAAAGCTTATATTATTTATT CGAGGGAATGCTATATCAGG

A_Asangui CAGCTCCTTTATCTACTAAT GAAGAGGAAATTAATAAGAA AAATGCGGGAGGGAGTAGTC AAAAGCTTATATTATTTATT CGGGGAAAGGCTATATCAGG

A_Bsangui CAGCTCCTTTATCTACTAAT GAAGAGGAAATTAATAAGAA AAATGCGGGAGGGAGTAGTC AAAAGCTTATATTATTTATT CGGGGAAAGGCTATATCAGG

A_Csangui CAGCTCCTTTATCTACTAAT GAAGAGGAAATTAATAAGAA AAATGCGGGAGGGAGTAGTC AAAAGCTTATATTATTTATT CGGGGAAAGGCTATATCAGG

A_Dsangui CAGCTCCTTTATCTACTAAT GAAGAGGAAATTAATAAGAA AAATGCGGGAGGGAGTAGTC AAAAGCTTATATTATTTATT CGGGGAAAGGCTATATCAGG

Doratogon AGCACCTAATATTAAAGGAA CAAGTCAGTTACCAAAGCCT CCAATTATAATAGGTATTAC TATAAAGAAGATTATGACGA AAGCGTGAGCAGTAACAATG

Mad_maxAS AGCTCCCAGTATTAAAGGGA CAAGTCAGTTCCCAAATCCT CCAATTATAACCGGTATTAC TATAAAGAAAATTATTACAA AGGCATGAGCTGTAACGATT

Mad_maxBS AGCTCCCAGTATTAAAGGGA CAAGTCAGTTCCCAAATCCT CCAATTATAACCGGTATTAC TATAAAGAAAATTATTACAA AGGCATGAGCTGTAACGATT

S_simplex GGCCCCCAATATTAATGGCA CTAATCAATTACCAAAACCT CCAATTATAATAGGCATTAC CATAAAGAAAATTATAACAA AGGCATGCGCAGTAACAATT

S_triareu AGCTCCTAACATTAAAGGGA CTAGTCAGTTTCCAAAGCCA CCAATTATAATAGGTATTAC TATGAAAAAAATTATTACAA AAGCATGAGCTGTTACAATT

C_AsemiSP AGCACCTAATATTAGAGGGA CAAGTCAATTTCCAAAACCT CCAATCATAATAGGTATAAC TATAAAGAAAATTATAACGA AAGCATGAGCTGTTACAATT

C_BsemiSP AGCACCTAATATTAAAGGGA CAAGTCAATTTCCAAAACCT CCAATCATAATAGGTATAAC TATAAAGAAAATTATAACGA AAGCATGAGCTGTTACAATT

A_Avampyr nnnnnnnnnnnnnnnnnnnn nnnnnnnnnnnnnnnnnnnn nnnnnnnnnnnnnnnnnnnn nnnnnnnnnnnnnnnnnnnn nnnnnnnnnnnnnnnnnnnn

A_Bvampyr TGCACCTAATATTAAAGGAA TTAATCAATTTCCAAAGCCT CCAATTATAATAGGTATAAC TATGAAGAAAATTATGACAA AAGCATGGGCTGTTACAATT

A_Cvampyr TGCACCTAATATTAAAGAAA TTAATCAATTTCCAAAGCCT CCAATTATAATAGGTATAAC TATGAAGAAAATTATAACAA AAGCATGAGCTGTTACAATT

A_Dvampyr nnnnnnnnnnnnnnnnnnnn nnnnnnnnnnnnnnnnnnnn nnnnnnnnnnnnnnnnnnnn nnnnnnnnnnnnnnnnnnnn nnnnnnnnnnnnnnnnnnnn

A_Ainfern AGCACCTAATATTAAAGGGA TTAATCAGTTTCCAAAACCC CCAATTATAATGGGTATAAC TATAAAAAAGATTATAACAA AAGCATGGGCTGTTACAATA

A_Binfern AGCACCTAATATTAAAGGGA TTAATCAGTTTCCAAAACCC CCAATTATAATGGGTATAAC TATAAAAAAGATTATAACAA AAGCATGGGCTGTTACAATA

A_Cinfern AGCACCCAATATTAAAGGAA TTAATCAGTTTCCAAAACCT CCAATCATAATAGGTATAAC TATAAAAAAGATTATAACAA AAGCATGAGCTGTTACAATA

A_Dinfern nnnnnnnnnnnnnnnnnnnn nnnnnnnnnnnnnnnnnnnn nnnnnnnnnnnnnnnnnnnn nnnnnnnnnnnnnnnnnnnn nnnnnnnnnnnnnnnnnnnn

A_Einfern AGCACCCAATATTAAAGGGA TTAATCAGTTTCCAAAACCT CCAATTATAATGGGTATAAC TATAAAAAAGATTATAACAA AAGCATGAGCCGTTACAATA

A_Acorall AGCACCTANTATTTAAAgAA NTAATTAATTTCCAAAGCCT CTAATTGTAATAGGTATGAC TATAAAAAAAATTATAAAAA AAGCATGAGCTGTTGCAATT

A_Bcorall AGCACCTAATATTTAAAgAA CTAATTAATTTCCAAAGCCT CTAATTGTAATAGGTATGAC TATAAAAAAAATTATAAAAA AAGCATGAGCTGTTGCAATT

A_nsp1229 AGCTCCTAATATTAAAGGAA CTAGTCAGTTTCCAAAGCCT CCAATTATAATAGGTATTAC TATAAAAAAAATTATTACAA ATGCATGAGCTGTTACAATA

A_nsp1396 AGCTCCCAATATTAGAGGAA TTAATCAATTTCCAAAACCT CCAATTATAATTGGCATAAC TATAAAAAAAATTATTACAA ATGCGTGGGCTGTTACAATT

A_Aerythr AGCACCCAATATTAAAGGGA CTAACCAATTACCAAAACCT CCAATTATAATAGGTATTAC TATAAAAAAAATTATAACAA AAGCGTGAGCTGTAACAATA

A_Berythr AGCCCCCAGTATTAAAGGAA CTAATCAATTACCAAAGCCT CCAATTATAATGGGTATAAC TATGAAAAAGATTATGACAA AGGCATGAGCTGTAACAATA

A_AhovaSP AGCTCCCAGTATTAAAGGAA CTAGTCAATTTCCAAAACCA CCAATTATAATAGGCATAAC TATAAAGAAAATTATTACAA AAGCGTGAGCTGTAACAATG

A_BhovaSP AGCTCCTAATATTAAAGGAA CTAGCCAATTTCCAAAACCA CCAATTATAATGGGTATAAC TATAAAAAAAATTATTACAA AAGCGTGAGCTGTAACAATA

A_ignipes AGCTCCTAACATTAAaGGAA CTAATCAATTTCCAAATCCT GcAATYATKATAGGTATAAC TATAAAGAAAATTAATCCAA MGGCGTGGGCTGTAACAATA

A_Bcowani AGCACCTAATATTAAAGGTA CTAATCAATTTCCAAATCCT CCAATTATAATTGGTATAAC TATGAAAAAAATTATAACAA AAGCATGAGCTGTTACAATA

A_Ccowani AGCACCTAATATTAAAGGTA CTAATCAATTTCCAAATCCT CCAATTATAATTGGTATAAC TATGAAAAAAATTATAACAA AAGCATGAGCTGTTACAATA

A_Dcowani AGCACCTAATATTAAAGGTA CTAATCAATTTCCAAATCCT CCAATTATAATTGGTATAAC TATGAAAAAAATTATAACAA AAGCATGAGCTGTTACAATA

A_Asangui AGCTCCTAATATTAAAGGTA CTAGTCAATTTCCGAATCCT CCAATTATAATTGGTATAAC TATGAAAAAAATTATAACAA AGGCGTGAGCTGTTACAATA

A_Bsangui AGCTCCTAATATTAAAGGTA CTAGTCAATTTCCGAATCCT CCAATTATAATTGGTATAAC TATGAAAAAAATTATAACAA AGGCGTGAGCTGTTACAATA

A_Csangui AGCTCCTAATATTAAAGGTA CTAGTCAATTTCCGAATCCT CCAATTATAATTGGTATAAC TATGAAAAAAATTATAACAA AGGCGTGAGCTGTTACAATA

A_Dsangui AGCTCCTAATATTAAAGGTA CTAGTCAATTTCCGAATCCT CCAATTATAATTGGTATAAC TATGAAAAAAATTATAACAA AGGCGTGAGCTGTTACAATA

Doratogon ACATTATAGATTTGGTCATC TCCAATTAAACTTCCTGGTT GGCTTAATTCTAAGCGAATT AATATGCTTAGAGAGGTACC AATTATTGCTGCTCAAGCCC

Mad_maxAS ACATTGTAGATTTGATCATC TCCAATTAAGCTACCTGGCT GGCCAAGCTCAAGACGAATG AGAACTCTAAGAGCAGTTCC TACTATCGCAGCTCAAGCAC

Mad_maxBS ACATTGTAGATTTGATCATC TCCAATTAAGCTACCTGGCT GGCCAAGCTCAAGACGAATG AGAACTCTAAGAGCAGTTCC TACTATCGCAGCTCAAGCAC

S_simplex ACGTTGTAGATTTGACTATC GTTAATTATACCTCCAGGTT GGCCAAGCTCAGTTCGAATT AATATTCTAAGTGCGGTACC AGAAAATGCAGCGCAAGCAC

S_triareu ACATTATAAATCTGGTCATC CCCAATTAGTCTTCCTGGTT GTCCTnGTTCTAGCCGAATT AGTATACTAAGAGCTGTTCC GATTATTGCGGCTCAAGCTC

C_AsemiSP ACATTATAGATTTGATCATC TCCAATTAAACTTCCTGGTT GGCCTAATTCTAATCGAATT AGTATTCTTAAGGCTGTTCC AACTATGGCTGCTCAAGCAC

C_BsemiSP ACATTATAGATTTGATCATC TCCAATTAAACTTCCTGGTT GGCCTAATTCTAATCGAATT AGTATTCTTAAGGCTGTTCC AACTATGGCTGCTCAAGCAC

A_Avampyr nnnnnnnnnnnnnnnnnnnn nnnnnnnnnnnnnnnnnnnn nnnnnnnnnnnnnnnnnnnn nnnnnnnnnnnnnnnnnnnn nnnnnnnnnnnnnnnnnnnn

A_Bvampyr ACATTATAAATTTGATCATC TCCAATTAAGCTNCCTGGTT GTCCTAATTCTAATCGAATT AATATACTTAAAGCTGTTCC AATTATTGCAGCTCAGGCTC

A_Cvampyr ACATTATAAATTTGATCATC TCCAATTAAGCTTCCTGGTT GTCCTAATTCTAATCGAATT AATATACTTAAAGCTGTTCC AATTATTGCAGCTCAGGCTC

A_Dvampyr nnnnnnnnnnnnnnnnnnnn nnnnnnnnnnnnnnnnnnnn nnnnnnnnnnnnnnnnnnnn nnnnnnnnnnnnnnnnnnnn nnnnnnnnnnnnnnnnnnnn

A_Ainfern ACATTATAAATTTGATCATC TCCAATTAAACTTCCTGGTT GGCCTAATTCTAATCGAATT AATATACTTAAAGCTGTTCC AATTATTGCCGCCCAAGCTC

A_Binfern ACATTATAAATTTGATCATC TCCAATTAAACTTCCTGGTT GGCCTAATTCTAATCGAATT AATATACTTAAAGCTGTTCC AATTATTGCCGCCCAAGCTC

A_Cinfern ACGTTATAAATTTGATCATC TCCAATTAAACTTCCTGGTT GGCCTAATTCTAATCGAATT AGTATACTTAAAGCTGTTCC AATTATTGCCGCCCAGGCTC

A_Dinfern nnnnnnnnnnnnnnnnnnnn nnnnnnnnnnnnnnnnnnnn nnnnnnnnnnnnnnnnnnnn nnnnnnnnnnnnnnnnnnnn nnnnnnnnnnnnnnnnnnnn

A_Einfern ACATTATAAATTTGATCATC TCCAATTAAACTTCCTGGTT GGCCTAATTCTAATCGAATT AGTATACTTAAAGCTGTCCC AATTATTGCCGCCCAGGCTC

A_Acorall ACATTATAAATTTGATCATC TCCAATTAGACTTCCNGGTT GACCTAATTCTAACCGAATT AGTATACATAAATCTGTCCC CCT-----------------

A_Bcorall ACATTATAAATTTGATCATC TCCAATTAGACTCTCAGGTT GACCTAATTCTAACCGAATT AGTATACATAAATCTGTCCC AATTATTGCAGCTCATGCTT

A_nsp1229 ACATTATAAATTTGATCATC TCCAATTAATCTTCCTGGTT GACCTAGCTCTAATCGAATT AATATACTTAAAGCTGTTCC AATTATTGCAGCCCATGCTC

A_nsp1396 ACATTATAAATTTGGTCATC TCCAATTAAACTTCCTGGTT GTCCCAATTCTAACCGAATC AATATACTTAAAGCTGTCCC AATTATTGCAGCTCATGCAC

A_Aerythr ACATTGTAAATTTGATCATC TCCAATTAGTCTTCCAGGTT GACCTAATTCTAATCGAATT AATATTCTTAAAGCTGTCCC AATTATTGCAGCTCATGCAC

A_Berythr ACGTTGTAAATTTGGTCATC TCCAATTAATCTCCCAGGTT GCCCTAATTCTAATCGAATT AATATTCTTAAAGCTGTTCC AATTATTGCAGCTCATGCAC

A_AhovaSP ACGTTATAAATTTGNTCATC TCCAATTAATCTACCTGGCT GACCTAATTCTAATCGAATC AATATTCTTAGGGCTGTTCC AATTATTGCAGCTCAAGCCC

A_BhovaSP ACATTATAAATTTGATCATC TCCAATTAAGCTACCTGGCT GCCCTAATTCTAATCGAATT AATATTCTTAAGGCTGTTCC AATTATTGCAGCTCAAGCCC

A_ignipes ACATTAtAAATTTGGTCATC TCCGATTAARCTACCTGGTT GGCCTAGYTCTAATCGAATT AATATTCTTAAGGCTGTTCC --------------------

A_Bcowani ACATTATAGATTTGGTCATC TCCAATTAAACTACCTGGTT GGCCTAATTCTAGTCGAATT AATATACTTAGAGCAGTTCC AATTATTGCAGCTCAGGCTC

A_Ccowani ACATTATAGATTTGGTCATC TCCAATTAAACTACCTGGTT GGCCTAATTCTAGTCGAATT AATATACTTAGAGCAGTTCc aATTATTGCAGCTCAGGCTC

A_Dcowani ACATTATAGATTTGGTCATC TCCAATTAAACTACCTGGTT GGCCTAATTCTAGTCGAATT AATATACTTAGAGCAgTTCC AATTATTGCAGCTCAGGCTC

A_Asangui ACATTGTAAATTTGGTCATC TCCGATTAAACTGCCTGGTT GACCTAGTTCTAGTCGAATT AATATACTTAAAGCGGTTCC AATTATCGCAGCTCAAGCTC

A_Bsangui ACATTGTAAATTTGGTCATC TCCGATTAAACTGCCTGGTT GACCTAGTTCTAGTCGAATT AATATACTTAAAGCGGTTCC AATTATCGCAGCTCAAGCTC

A_Csangui ACATTGTAAATTTGGTCATC TCCGATTAAACTGCCTGGTT GACCTAGTTCTAGTCGAATT AATATACTTAAAGCGGTTCC AATTATCGCAGCTCAAGCTC

A_Dsangui ACATTGTAAATTTGGTCATC TCCGATTAAACTGCCTGGTT GACCTAGTTCTAGTCGAATT AATATACTTAAAGCGGTTCC AATTATCGCAGCTCAAGCTC

Doratogon CAAAAATTAAATATATAGGG TTTTTTGAT-TTATATAAAA AATCAGGCCTGCCCACTGA- -----AAATTTGAAGGGCTG CAGTATTTTGACTGTACAAA

Mad_maxAS CAAAAATTAAATATATTGnn nnnnnnnnnnnnnnnnnnnn nnnnnnnnnnnnnnnnnnnn nnnnnnnnnnnnnnnnnnnn nnnnnnnnnnnnnnnnnnnn

Mad_maxBS CAAAAATTAAATATATTGnn nnnnnnnnnnnnnnnnnnnn nnnnnnnnnnnnnnnnnnnn nnnnnnnnnnnnnnnnnnnn nnnnnnnnnnnnnnnnnnnn

S_simplex CCAAAATTAAATATATTGGG TTTTTTGTTGTTAAATAAAA AATCTGGCCTGCCCACTGAT -----TTATTTGAAGGGCTG CAGTATATTGACTGTACAAA

S_triareu CAAAAATCAAGTATATAGGG TTTTTTGTAGTTAAATAAAA AATCTGGCCTGCCCACTGAA ATA--TTATTTGAAGGGCTG CAGTATATTGACTGTACAAA

C_AsemiSP CAAAAACTAAGTATATAGnn nnnnnnnnnnnnnnnnnnnn nnnnnnnnnnnnnnnnnnnn nnnnnnnnnnnnnnnnnnnn nnnnnnnnnnnnnnnnnnnn

C_BsemiSP CAAAAAT-----------nn nnnnnnnnnnnnnnnnnnnn nnnnnnnnnnnnnnnnnnnn nnnnnnnnnnnnnnnnnnnn nnnnnnnnnnnnnnnnnnnn

A_Avampyr nnnnnnnnnnnnnnnnnnGG TTTTTTGTTATTAAATAAAA AATCTGGCCTGCCCACTGAA ATGA-ATTATTGAAGGGCTG CAGTATATTGACTGTACAAA

A_Bvampyr CAAAAATTAAATATATAGnn nnnnnnnnnnnnnnnnnnnn nnnnnnnnnnnnnnnnnnnn nnnnnnnnnnnnnnnnnnnn nnnnnnnnnnnnnnnnnnnn

A_Cvampyr CAAAAATTAAATATATAGGG TTTTTTGTTATTAAATAAAA AATCTGGCCTGTCCGCTGAA TG---ATTATTGAAGGGCTG CAGTATATTGACTGTACAAA

A_Dvampyr nnnnnnnnnnnnnnnnnnnn nnnnnnnnnnnnnnnnnnnn nnnnnnnnnnnnnnnnnnnn nnnnnnnnnnnnnnnnnnnn nnnnnnnnnnnnnnnnnnnn

A_Ainfern CAAAAACTAAATATATAGGG TTTTTTGTTGTTAGATAAAA AATCCGGCCTGCCCACTGAA ATGA-GCAATTGAAGGGCTG CAGTATATTGACTGTACAAA

A_Binfern CAAAAACTAAATATATAGGG TTTTTTGTTGTTAGATAAAA AATCCGGCCTGCCCACTGAA ATGA-GCAATTGAAGGGCTG CAGTATATTGACTGTACAAA

A_Cinfern CAAAAACTAAATATATAGGG TTTTTTGTTGTTAGATAAAA AATCCGGCCTGCCCACTGAA ATGA-GTAATTGAAGGGCTG CAGTATATTGACTGTACAAA

A_Dinfern nnnnnnnnnnnnnnnnnnGG TTTTTTGTTGTTAGATAAAA AATCCGGCCTGCCCACTGAA ATGA-GTAATTGAAGGGCTG CAGTATATTGACTGTACAAA

A_Einfern CAAAAACTAAATATATAGGG TTTTTTGTTGTTAGATAAAA AATCCGGCCTGCCCACTGAA ATGA-GTAATTGAAGGGCTG CAGTATATTGACTGTACAAA

A_Acorall ------------------GG TTTTTTGTTATTAAATAAAA AATCTGGCCTGCCCACTGAA AATGTATAATTGAAGGGCTG CAGTATATTGACTGTACAAA

A_Bcorall CAAAGATTAAATATATATGG TTTTTTGTTATTAAATAAAA AATCTGGCCTGCCCACTGAA AATGTATAATTGAAGGGCTG CAGTATATTGACTGTACAAA

A_nsp1229 CAAAAATTAAATATATAGGG TTTTTTGTTGTTATATAAAA AATCTGGCCTGCCCACTGAA AATGAGTAATTAAAGGGCTG CAGTATATTGACTGTACAAA

A_nsp1396 CAAAAATTAAATATATAGGG TTTTTTGTTATTAAATAAAA AATCTGTCCTGCCCACTGAT ATAA-GTAATTAAAGGGCTG CAGTATATTGACTGTACAAA

A_Aerythr CAAAAATTAAGTATATAGGG TTTTTTGTTGTTAAATAAAA AATCTGGCCTGCCCACTGAG T----ATAATTAAAGGGCTG CAGTATATTGACTGTACAAA

A_Berythr CAAAAATTAAATATATAGnn nnnnnnnnnnnnnnnnnnnn nnnnnnnnnnnnnnnnnnnn nnnnnnnnnnnnnnnnnnnn nnnnnnnnnnnnnnnnnnnn

A_AhovaSP CAAAGACTAAGTATATAGGG TTTTTTGTTGTTAAATAAAA AATCTGGCCTGCCCACTGA- -----AAAGTTGAAGGGCTG CAGTATATTGACTGTACAAA

A_BhovaSP CAAAGACTAAGTATATAGGG TTTTTTGTTGTTAAATAAAA AATCTGGCCTGCCCACTGA- -----AAAATTAAAGGGCTG CAGTATATTGACTGTACAAA

A_ignipes -------------------- -------------------- -------------------- -------------------G CAGGATATTGACTGTACAAA

A_Bcowani CaAAGATTAAATATATAGnn nnnnnnnnnnnnnnnnnnnn nnnnnnnnnnnnnnnnnnnn nnnnnnnnnnnnnnnnnnnn nnnnnnnnnnnnnnnnnnnn

A_Ccowani CGAAGATTAAATATATAG-- -------------------- -------CCTGCCCACTGAG TA---TTAATTGAAGGGSTG CAGTATATTGACTGKACAAA

A_Dcowani CGAAGATTAAATATATAG-- -------------------- ---------TGCCCACTGAG TA---TTAATTGAAGGGCTG CAGTATATTGACTGTACAAA

A_Asangui CAAAAATTAAATATATAGGG TTTTTTGTTGTTAAATAAAM AATCYGGCCTGCCCACTGAA TA---TTGGTTAAAGGGCTG CAGTATATYGACTGTACAAA

A_Bsangui CAAAAATTAAATATATAGnn nnnnnnnnnnnnnnnnnnnn nnnnnnnnnnnnnnnnnnnn nnnnnnnnnnnnnnnnnnnn nnnnnnnnnnnnnnnnnnnn

A_Csangui CAAAAATTAAATATATAGnn nnnnnnnnnnnnnnnnnnnn nnnnnnnnnnnnnnnnnnnn nnnnnnnnnnnnnnnnnnnn nnnnnnnnnnnnnnnnnnnn

A_Dsangui CAAAAATTAAATATATAG-- -------------------- -------------------- -------------------- --------------------

Doratogon GGTAGCATAATCATTAGTCT TTTAATTAAGGACTGGTATG AAAGGTTTGACGGAGATTGA CTGTCTCAATTTTAAATTAT GAATTTAATTTTTAAGGAAA

Mad_maxAS nnnnnnnnnnnnnnnnnnnn nnnnnnnnnnnnnnnnnnnn nnnnnnnnnnnnnnnnnnnn nnnnnnnnnnnnnnnnnnnn nnnnnnnnnnnnnnnnnnnn

Mad_maxBS nnnnnnnnnnnnnnnnnnnn nnnnnnnnnnnnnnnnnnnn nnnnnnnnnnnnnnnnnnnn nnnnnnnnnnnnnnnnnnnn nnnnnnnnnnnnnnnnnnnn

S_simplex GGTAGCATATTCATTAGTCT TTTTATTGAGGACTGGAATG AAAGGCTAGACAGGCTTTAG CTGTCTCTAGGGCATTGAAT AAAATTTTTTTCTTTGTGAA

S_triareu GGTAGCATATTCATTAGTCT TTTGATTGAGGACTGGAATG AATGGCTAGACAGGCTTTAA CTGTCTCTGAAGTATAAATT AAAGTTTTTTTCCTTGTGAA

C_AsemiSP nnnnnnnnnnnnnnnnnnnn nnnnnnnnnnnnnnnnnnnn nnnnnnnnnnnnnnnnnnnn nnnnnnnnnnnnnnnnnnnn nnnnnnnnnnnnnnnnnnnn

C_BsemiSP nnnnnnnnnnnnnnnnnnnn nnnnnnnnnnnnnnnnnnnn nnnnnnnnnnnnnnnnnnnn nnnnnnnnnnnnnnnnnnnn nnnnnnnnnnnnnnnnnnnn

A_Avampyr GGTAGCATAATCATTAGTCT TTTAATTGAGGACTGGAATG AAAGGTCTAACAAGCTTTAA CTGTCTCTAAAATATTTTAT TAAATTATTTTTTTTGTGAA

A_Bvampyr nnnnnnnnnnnnnnnnnnnn nnnnnnnnnnnnnnnnnnnn nnnnnnnnnnnnnnnnnnnn nnnnnnnnnnnnnnnnnnnn nnnnnnnnnnnnnnnnnnnn

A_Cvampyr GGTAACATAATCATTAGTCT TTTAATTGAGGACTGGAATG AAAGGTCTAACAAGATTTAA CTGTCTCTAAAATATTTTAT TAAATTATTTTTTTTGTGAA

A_Dvampyr nnnnnnnnnnnnnnnnnnnn nnnnnnnnnnnnnnnnnnnn nnnnnnnnnnnnnnnnnnnn nnnnnnnnnnnnnnnnnnnn nnnnnnnnnnnnnnnnnnnn

A_Ainfern GGTAGCATAATCATTAGTCT TTTAATTGAAGACTGGAATG AAAGGTTTAACAAGCTTTAA CTGTCTCTAGAATATTTTAT TAAATTATTTTTTTTGTGAA

A_Binfern GGTAGCATAATCATTAGTCT TTTAATTGAAGACTGGAATG AAAGGTTTAACAAGCTTTAA CTGTCTCTAGAATATTTTAT TAAATTATTTTTTTTGTGAA

A_Cinfern GGTAGCATAATCATTAGTCT TTTAATTGAAGACTGGAATG AAAGGTTTAACAAGCTTTAA CTGTCTCTATAATATTTTAT TAAATTATTTTTTTTGTGAA

A_Dinfern GGTAGCATAATCATTAGTCT TTTAATTGAAGACTGGAATG AAAGGTTTAACAAGCTTTAA CTGTCTCTATAATATTTTAT TAAATTATTTTTTTTGTGAA

A_Einfern GGTAGCATAATCATTAGTCT TTTAATTGAAGACTGGAATG AAAGGTTTAACAAGCTTTAA CTGTCTCTGGAATATTTTAT TAAATTATTTTTTTTGTGAA

A_Acorall GGTAGCATAATCATTAGTCT TTTAATTGAGGACTGGAATG AAAGGTCTAACAAGCTTTAA CTGTCTCTAGGATATTTTAT TAAATTATTTTTTTTGTGAA

A_Bcorall GGTAGCATAATCATTAGTCT TTTAATTGAGGACTGGAATG AAAGGTCTAACAAGCTTTAA CTGTCTTTAGGATATTTTAT TAAATTATTTTTTTTGTGAA

A_nsp1229 GGTAGCATAATCATTAGTCT TTTAATTGAAGACTGGAATG AAAGGTTTAACAAGTTTTAA CTGTCTCTGAAATATTTTAT TAAGTTATTTTTTTTGTGAA

A_nsp1396 GGTAGCATAATCATTAGTCT TTTAATTGAGGACTGGAATG AAAGGTTTAACAAGCTTTAA CTGTCTCTAGGGTATTTTAT TAATTTATTTTTTTTGTGAA

A_Aerythr GGTAGCATAATCATTAGTCT TTTAATTGAAGACTTGAATG AAAGGTCTAACAAGTTTTAA CTGTCTCTAAAATACTTTAT TAAATTATTTTTTTTGTGAA

A_Berythr nnnnnnnnnnnnnnnnnnnn nnnnnnnnnnnnnnnnnnnn nnnnnnnnnnnnnnnnnnnn nnnnnnnnnnnnnnnnnnnn nnnnnnnnnnnnnnnnnnnn

A_AhovaSP GGTAGCATAATAATTAGTCT TTTAATTGAAGACTGGAATG AAAGGTCTCACAAGTTTTAA CTGTCTCTGGGATATTTTAT TAAATTATTTTCTTTGTGAA

A_BhovaSP GGTAGCATAATAATTAGTCT TTTAATTGAAGACTGGAATG AAAGGTCTAACAAGTTTTGA CTGTCTCTGAAGTATTTTAC TAAGTTATTTTCTTTGTGAA

A_ignipes GGTAGCATAATAATTAGTCT TTTAATTGAAGACTGGAATG AAAGGTCCAACAAGCTTTAA CTGTCTCTGAAGTATTTTAT TAAMTTATTTTTTTTGTAAA

A_Bcowani nnnnnnnnnnnnnnnnnnnn nnnnnnnnnnnnnnnnnnnn nnnnnnnnnnnnnnnnnnnn nnnnnnnnnnnnnnnnnnnn nnnnnnnnnnnnnnnnnnnn

A_Ccowani GGTAGCATAATCATTAGTCT TTTAATTGAAGACTGGAAKG AAAGGTCTAACAAGTTTTAG CTGTCTCTAAAGTATTTTAT TAACTTATTTTTTTTGTGAA

A_Dcowani GGTAGCATAATCATTAGTCT TTTAATTGAAGACTGGAATG AAAGGTCTAACAAGTTTTAG CTGTCTCTAAAGTATTTTAT TAACTTATTTTTTTTGTGAA

A_Asangui GGTAGCATAATCMTTAGTCT TTTAATTGAAGACTGGAATG AAAGGTCTAACAAGCTTTAA CTGTCTCTAAAAAATTTTAT TAAGTTATTTTTTTTGTGAA

A_Bsangui nnnnnnnnnnnnnnnnnnnn nnnnnnnnnnnnnnnnnnnn nnnnnnnnnnnnnnnnnnnn nnnnnnnnnnnnnnnnnnnn nnnnnnnnnnnnnnnnnnnn

A_Csangui nnnnnnnnnnnnnnnnnnnn nnnnnnnnnnnnnnnnnnnn nnnnnnnnnnnnnnnnnnnn nnnnnnnnnnnnnnnnnnnn nnnnnnnnnnnnnnnnnnnn

A_Dsangui -------------------- -------------------- -------------------- ----------AAAATTTTAT TAAGTTATTTTTTTTGTGAA

Doratogon AGAGCTTAAATATTACAAAG GGACGAGAAGACCCTATCAA ATTTCA-TTATTAATAAGTA AAATTTTATTTTGATAAAAA TTTTATTTTCTAAATAATTT

Mad_maxAS nnnnnnnnnnnnnnnnnnnn nnnnnnnnnnnnnnnnnnnn nnnnnnnnnnnnnnnnnnnn nnnnnnnnnnnnnnnnnnnn nnnnnnnnnnnnnnnnnnnn

Mad_maxBS nnnnnnnnnnnnnnnnnnnn nnnnnnnnnnnnnnnnnnnn nnnnnnnnnnnnnnnnnnnn nnnnnnnnnnnnnnnnnnnn nnnnnnnnnnnnnnnnnnnn

S_simplex AATTCAGGGATAAAATAAAG GGACAAGAAGACCCTATCAA ACTTAT-TTTAGGTAACGTG TTACTTTA-AGAGATAAAAG TAATA-TTGAAGATAAAATT

S_triareu AAAACAAGGATAATTTAAAG GGACAAGAAGACCCTATCAA ACTTAT-TTTAGTCTCAATT TTATTTTT-ATATATAAAAG TGGTA-TTGTGGGGAAAATT

C_AsemiSP nnnnnnnnnnnnnnnnnnnn nnnnnnnnnnnnnnnnnnnn nnnnnnnnnnnnnnnnnnnn nnnnnnnnnnnnnnnnnnnn nnnnnnnnnnnnnnnnnnnn

C_BsemiSP nnnnnnnnnnnnnnnnnnnn nnnnnnnnnnnnnnnnnnnn nnnnnnnnnnnnnnnnnnnn nnnnnnnnnnnnnnnnnnnn nnnnnnnnnnnnnnnnnnnn

A_Avampyr AAAACAAAAATGTAACAAAG GGACAAGAAGACCCTGTCAA ACTTATATTTATTTAAAATA TAATTTAA-GTTAATAAAGG TAGTA-TATTAGTTAAATTT

A_Bvampyr nnnnnnnnnnnnnnnnnnnn nnnnnnnnnnnnnnnnnnnn nnnnnnnnnnnnnnnnnnnn nnnnnnnnnnnnnnnnnnnn nnnnnnnnnnnnnnnnnnnn

A_Cvampyr AAAACAAAAATGTAACAAAG GGACAAGAAGACCCTGTCAA ACTTATATTTATTTAAAATA TAATTTAA-GTTAATAAAGG TAGTA-TATTGGTTAAATTT

A_Dvampyr nnnnnnnnnnnnnnnnnnnn nnnnnnnnnnnnnnnnnnnn nnnnnnnnnnnnnnnnnnnn nnnnnnnnnnnnnnnnnnnn nnnnnnnnnnnnnnnnnnnn

A_Ainfern AAAACAAAAATGGATTAAAG GGACAAGAAGACCCTATCAA GCTTATATTTATTTAAAATA TAATTTAA-GTGAATAAAGG TAATA-TATTAGTTAAATTT

A_Binfern AAAACAAAAATGGATTAAAG GGACAAGAAGACCCTATCAA GCTTATATTTATTTAAAATA TAATTTAA-GTGAATAAAGG TAATA-TATTAGTTAAATTT

A_Cinfern AAAACAAAAATGGAATAAAG GGACAAGAAGACCCTATCAA ACTTATATTTATTTAAAATA TAATTTGA-ATGAATAAAGG TAATA-TATTAGTTAAATTT

A_Dinfern AAAACAAAAATGGAATAAAG GGACAAGAAGACCCTATCAA ACTTATATTTATTTAAAATA TAATTTGA-ATGAATAAAGG TAATA-TATTAGTTAAATTT

A_Einfern AAAACAAAAATGGAATAAAG GGACAAGAAGACCCTATCAA ACTTATATTTATTTAAAATA TAATTTGA-ATGAATAAAGG TAATA-TATTAGTTAAATTT

A_Acorall AAAACAAAAATGTAATAAAG GGACAAGAAGACCCTATCAA ACTTGTATTTATTTAATGTA CAATTTAA-GTTAATAAAGA TAGTA-TTTTAGTTAAATTT

A_Bcorall AAAACAAAAATGTAATAAAG GGACAAGAAGACCCTATCAA ACTTGTATTTATTTAATGTA CAATTTAA-GTTAATAAAGA TAGTA-TTTTAGTTAAATTT

A_nsp1229 AAAACAAAAATTAAGTAAAG GGACAAGAAGACCCTATCAA GCTTAGATTTATTTAAGATA TAGCTTAA-ATTAATAAAAG TAATA-TGTTGGTTAAAATT

A_nsp1396 AAAACAAAAATAAGGTAAAG GGACAAGAAGACCCTATCAA ACTTGAATTTATTTAAGGTA TAGCTTGA-ATTAATAAAAG TAATATTTTTAGTTAAAATT

A_Aerythr AAAACAAAGATGAAATAAAG GGACAAGAAGACCCTATCAA ACTTAT-TTTATCTTATGTA TAATTTAA----ATTAATGA TAGTA-TATTAGTTAAAATT

A_Berythr nnnnnnnnnnnnnnnnnnnn nnnnnnnnnnnnnnnnnnnn nnnnnnnnnnnnnnnnnnnn nnnnnnnnnnnnnnnnnnnn nnnnnnnnnnnnnnnnnnnn

A_AhovaSP AAAACAAAGATGAATTAAAG GGACAAGAAGACCCTATCAA ACTTAT-TTTATTTTGTATA TAATTTGA-ATAAATAAAGA TAATA-TGTTAGTTAAAATT

A_BhovaSP AAAACAAAGATGAATTAAAG GGACAAGAAGACCCTATCAA GCTTAT-TTTATTTTATATA TAATTTAA-GTAAATAAGAG TAATA-TATTAGTTAAAATT

A_ignipes AAAACAAAGATGAATTAAAG GCACAAGAAGACCCTATCAA ACTTAT-TTTATTTTGTATA TAATTTAA-GTAAATAASAG TAATA-TGCTAGTTAAAATT

A_Bcowani nnnnnnnnnnnnnnnnnnnn nnnnnnnnnnnnnnnnnnnn nnnnnnnnnnnnnnnnnnnn nnnnnnnnnnnnnnnnnnnn nnnnnnnnnnnnnnnnnnnn

A_Ccowani AAAACAAAAATGAAATAAAG GGACAAGAAGACCCTATCAA ACYTAT-TTTATTTTATATA TTATTTAA----NNNNNNNN NAATG-TATTAGTTAAAATT

A_Dcowani AAAACAAAAATGAAATAAAG GGACAAGAAGACCCTATCAA ACTTAT-TTTATTTTATATA TTATTTAA-----AGATAAA TAATG-TATTAGTTAAAATT

A_Asangui AAAACAAAAATGAGATAAAG GGACAAGAAGACCCTATCAA ACTTAT-TTTATTTTATGTA TTGTTTAA-----ATATAAA TATTG-TGTTAGTTAAAATT

A_Bsangui nnnnnnnnnnnnnnnnnnnn nnnnnnnnnnnnnnnnnnnn nnnnnnnnnnnnnnnnnnnn nnnnnnnnnnnnnnnnnnnn nnnnnnnnnnnnnnnnnnnn

A_Csangui nnnnnnnnnnnnnnnnnnnn nnnnnnnnnnnnnnnnnnnn nnnnnnnnnnnnnnnnnnnn nnnnnnnnnnnnnnnnnnnn nnnnnnnnnnnnnnnnnnnn

A_Dsangui AAAACAAAAATGAGATAAAG GGACAAGAAGACCCTATCAA ACTTAT-TTTATTTTATGTA TTGTTTAA-----ATATAAA TAATG-TGTTAGTTAAAATT

Doratogon TGCTGGGGCGGCATGT-AAT TAACATTATTAAATTTTAAT ATTTTGTTTAGCGTTAATAT ---GATCCACTTAGTG--TG ATATTAAGAATAAATTACTG

Mad_maxAS nnnnnnnnnnnnnnnnnnnn nnnnnnnnnnnnnnnnnnnn nnnnnnnnnnnnnnnnnnnn nnnnnnnnnnnnnnnnnnnn nnnnnnnnnnnnnnnnnnnn

Mad_maxBS nnnnnnnnnnnnnnnnnnnn nnnnnnnnnnnnnnnnnnnn nnnnnnnnnnnnnnnnnnnn nnnnnnnnnnnnnnnnnnnn nnnnnnnnnnnnnnnnnnnn

S_simplex TACTGGGGCGGTAAGTAATA ATACATTATTTTTATTTAAC AACTTAATTGGCTTT--TAT AAGGATCCAT--GTAGATTG ATATTGAGAAAAAGTTACTG

S_triareu TGCTGGGGCGGTGAGTAATA AAGCGTTATTTTTATTTAAC AAATTAAGTGGC-TTATAAT AAGGATCCAA--TGTGGTTG ATAGTAAGAAAAAGTTACTG

C_AsemiSP nnnnnnnnnnnnnnnnnnnn nnnnnnnnnnnnnnnnnnnn nnnnnnnnnnnnnnnnnnnn nnnnnnnnnnnnnnnnnnnn nnnnnnnnnnnnnnnnnnnn

C_BsemiSP nnnnnnnnnnnnnnnnnnnn nnnnnnnnnnnnnnnnnnnn nnnnnnnnnnnnnnnnnnnn nnnnnnnnnnnnnnnnnnnn nnnnnnnnnnnnnnnnnnnn

A_Avampyr TACTGGGGCGGTAAGT-ATA AAACATTATTTGTGTTTAAT GTTGTTAATAGC-TAAATAT AAGGATCCAA-GATTGATTG ATAGTAAGAAAGAGTTACTG

A_Bvampyr nnnnnnnnnnnnnnnnnnnn nnnnnnnnnnnnnnnnnnnn nnnnnnnnnnnnnnnnnnnn nnnnnnnnnnnnnnnnnnnn nnnnnnnnnnnnnnnnnnnn

A_Cvampyr TACTGGGGCGGTAAGT-ATA AAACATTATTTGTGTTTAAT GTTGTTAATAGC-TAAATAT AAGGATCCAA-GATTGATTG ATAGTAAGAAAGAGTTACTG

A_Dvampyr nnnnnnnnnnnnnnnnnnnn nnnnnnnnnnnnnnnnnnnn nnnnnnnnnnnnnnnnnnnn nnnnnnnnnnnnnnnnnnnn nnnnnnnnnnnnnnnnnnnn

A_Ainfern TACTGGGGCGGTAAGT-ATA AAACATTATTTGTGTTTAAT ATTATTAATAGC-TAGATGT AAGGATCCAA-GGTTGATTG ACAATAAGAGAGAGTTACTG

A_Binfern TACTGGGGCGGTAAGT-ATA AAACATTATTTGTGTTTAAT ATTATTAATAGC-TAGATGT AAGGATCCAA-GGTTGATTG ACAATAAGAGAGAGTTACTG

A_Cinfern TACTGGGGCGGTAAGT-ATA AAACATTATTTGTGTTTAAT ATTATTAATAGC-TAGATGT AAGGATCCAA-AATTGATTG ACAATAAGAAAGAGTTACTG

A_Dinfern TACTGGGGCGGTAAGT-ATA AAACATTATTTGTGTTTAAT ATTATTAATAGC-TAGATGT AAGGATCCAA-AATTGATTG ACAATAAGAAAGAGTTACTG

A_Einfern TACTGGGGCGGTAAGT-ATA AAACATTATTTGTGTTTAAT ATTATTAATAGC-TAGATGT AAGGATCCAA-AATTGATTG ACAATAAGAGAAAGTTACTG

A_Acorall TACTGGGGCGGTGAGT-ATG AAACGTTATTTGTATTTAAT ATTATTAATAGC-TTAATAT AAGGATCCAA-GAGTGATTG ATAGTAAGAGAGAGTTACTG

A_Bcorall TACTGGGGCGGTGAGT-ATG AAACGTTATTTGTATTTAAT ATTATTAATAGC-TTAATAT AAGGATCCAA-GAGTGATTG ATAGTAAGAGAGAGTTACTG

A_nsp1229 TACTGGGGCGGTAAGT-ATA AAACATTATTTGTATTTAAT GTTATTAGTAGC-TAAATTT AAGGATCCAA-AAATGATTG ATAAAAAGAAAGAGTTACTG

A_nsp1396 TACTGGGGCGGTAAGT-ATA AAACATTATTTGTATTTAAT ATTATTAATAAC-CAGATAT AAGGATCCAA-GATTGATTG ATAGAGAGAGAGAGTTACTG

A_Aerythr TACTGGGGCGGTAAGT-ATA AAACGTTATTTGTATGTAAT ATCTTTGATAAC-TTAATAT AAGGATCCAA-AATTGATTG ATAGTAAGAAAAAGTTACTG

A_Berythr nnnnnnnnnnnnnnnnnnnn nnnnnnnnnnnnnnnnnnnn nnnnnnnnnnnnnnnnnnnn nnnnnnnnnnnnnnnnnnnn nnnnnnnnnnnnnnnnnnnn

A_AhovaSP TACTGGGGCGGTAAGT-ATA AAACATTATTTAATTAAAAT ATCTTTAGTAGC-TTAATAT AAGGATCCAAGAGTTGATTG ATAATGAGAAAAAGTTACTG

A_BhovaSP TACTGGGGCGGTAAGT-ATA AAACATTATTTAATTAAAAT ATTTTTAACAGC-TTAATAT AAGGATCCAAAAATTGATTG ATAATAAGAAAAAGTTACTG

A_ignipes TACTGGGGCGGTGAGT-ATA AAACCTTATTTAGGTACAAT ATTTTTAATGGC-TTAATAT AAGGATCCAAAGATTGATTG ATAATAAAAAAAAGTTACTG

A_Bcowani nnnnnnnnnnnnnnnnnnnn nnnnnnnnnnnnnnnnnnnn nnnnnnnnnnnnnnnnnnnn nnnnnnnnnnnnnnnnnnnn nnnnnnnnnnnnnnnnnnnn

A_Ccowani TACTGGGGCGGTAGGT-ATA GAACATTATTTATATATAAT ATCTTTAATGGC-TTAATAT AAGGATCCAA-AATTGATTG ATAATGAGAAAAAGTTACTG

A_Dcowani TACTGGGGCGGTAGGT-ATA GAACATTATTTATATATAAT ATCTTTAATGGC-TTAATAT AAGGATCCAA-AATTGATTG ATAATGAGAAAAAGTTACTG

A_Asangui TACTGGGGCGGTAGGT-ATA AAACATTATTGATATATAAT ATTTTTAATGGC-TTAATAT AAGGATCCAA-AATTGATTG ATAATAAGAAGAAGTTACTG

A_Bsangui nnnnnnnnnnnnnnnnnnnn nnnnnnnnnnnnnnnnnnnn nnnnnnnnnnnnnnnnnnnn nnnnnnnnnnnnnnnnnnnn nnnnnnnnnnnnnnnnnnnn

A_Csangui nnnnnnnnnnnnnnnnnnnn nnnnnnnnnnnnnnnnnnnn nnnnnnnnnnnnnnnnnnnn nnnnnnnnnnnnnnnnnnnn nnnnnnnnnnnnnnnnnnnn

A_Dsangui TACTGGGGCGGTAGGT-ATA AAACATTATTTATATATAAT ATTTTTAATGGC-TTAATAT AAGGATCCAA-AATTGATTG ATAATAAGAAGAAGTTACTG

Doratogon TAGGGATAACAGCGTAATAT TTTTTGAGAGTTCTTATTGA CAAAAATGTTTGCGACCTCG ATGTTGGATTAAGAAATCTT ATTGGTGCAGTAGCTGA---

Mad_maxAS nnnnnnnnnnnnnnnnnnnn nnnnnnnnnnnnnnnnnnnn nnnnnnnnnnnnnnnnnnnn nnnnnnnnnnnnnnnnnnnn nnnnnnnnnnnnnnnnnnnn

Mad_maxBS nnnnnnnnnnnnnnnnnnnn nnnnnnnnnnnnnnnnnnnn nnnnnnnnnnnnnnnnnnnn nnnnnnnnnnnnnnnnnnnn nnnnnnnnnnnnnnnnnnnn

S_simplex TAGGGATAACAGCGTAATGT TCTTTGAGAGTTCATATTGA CAAAAATGTTTGCGACCTCG ATGTTGGATTAAGAAGTCTT TTTGGTGCAGTAGCTAA-TA

S_triareu TAGGGATAACAGCGTAATAT TATTTGAGAGTTCATATTGA CAATAATGTTTGCGACCTCG ATGTTGGATTAAGAAGTCTT TTTGGTGCAGTAGCTGA-TA

C_AsemiSP nnnnnnnnnnnnnnnnnnnn nnnnnnnnnnnnnnnnnnnn nnnnnnnnnnnnnnnnnnnn nnnnnnnnnnnnnnnnnnnn nnnnnnnnnnnnnnnnnnnn

C_BsemiSP nnnnnnnnnnnnnnnnnnnn nnnnnnnnnnnnnnnnnnnn nnnnnnnnnnnnnnnnnnnn nnnnnnnnnnnnnnnnnnnn nnnnnnnnnnnnnnnnnnnn

A_Avampyr TAGGGATAACAGCGTAATAT TTTYTGAGAGWTCWTATTGA CAAAWATGTTTGCGACCTCG ATGTTGGATTAAGMA----- --------------------

A_Bvampyr nnnnnnnnnnnnnnnnnnnn nnnnnnnnnnnnnnnnnnnn nnnnnnnnnnnnnnnnnnnn nnnnnnnnnnnnnnnnnnnn nnnnnnnnnnnnnnnnnnnn

A_Cvampyr TAGGGATAACAGCGTAATAT TTTTTGAGAGTTCTTATTGA CAAAAATGTTTGCGACCTCG ATGTTGGATTAAGACATTT- --------------------

A_Dvampyr nnnnnnnnnnnnnnnnnnnn nnnnnnnnnnnnnnnnnnnn nnnnnnnnnnnnnnnnnnnn nnnnnnnnnnnnnnnnnnnn nnnnnnnnnnnnnnnnnnnn

A_Ainfern TAGGGATAACAGCGTAATGT TTTTTGAGAGTTCATATTGA CAAAGATGTTTGCGACCTCG ATGTTGGATTAAGAAGTCTT ACTGATGCAGGAGTTAG-TA

A_Binfern TAGGGATAACAGCGTAATGT TTTTTGAGAGTTCATATTGA CAAAGATGTTTGCGACCTCG ATGTTGGATTAAGAAGTCTT ACTGATGCAGGAGTTAG-TA

A_Cinfern TAGGGATAACAGCGTAATGT TTTTTGAGAGTTCATATTGA CAAAGATGTTTGCGACCTCG ATGTTGGATTAAGAAGTCTT ACTGATGCAGGAGTTAG-TA

A_Dinfern TAGGGATAACAGCGTAATGT TTTTTGAGAGTTCATATTGA CAAAGATGTTTGCGACCTCG ATGTTGGATTAAGAAGTCTT ACTGATGCAGGAGTTAG-TA

A_Einfern TAGGGATAACAGCGTAATGT TTTTTGAGAGTTCATATTGA CAAAGATGTTTGCGACCTCG ATGTTGGATTAAGAAGTCTT ACTGATGCAGGAGTTAG-TA

A_Acorall TAGGGATAACAGCGTAATAT TTTTTGAGAGTTCATATTGA CAAAAATGTTTGCGACCTCG ATGTTGGATTAAGAAGTCTT ACTGGTGCAGTAGTCAG-TA

A_Bcorall TAGGGATAACAGCGTAATAT TTTTTGAGAGTTCATATTGA CAAAAATGTTTGCGACCTCG ATGTTGGATTAAGAAGTCTT ACTGGTGCAGTAGTCAG-TA

A_nsp1229 TAGGGATAACAGCGTAATAT TTTTTGAGAGTTCTTATTGA CAAGAATGTTTGCGACCTCG ATGTTGGATTAAGAAGTCTT TCTGATGCAGAAGTTAG-TA

A_nsp1396 TAGGGATAACAGCGTAATAT TTTTTGAGAGTTCATATTGA CAAGAATGTTTGCGACCTCG ATGTTGGATTAGGAAATCTT TCTGATGCAGAAGTCAG-TG

A_Aerythr TAGGGATAACAGCGTAATAT TTTTTGAGAGTTCATATTGA CAAAAGTGTTTGCGACCTCG ATGTTGGATTAAGAAGTCTT ATTGGTGCAAAAGTCAATTT

A_Berythr nnnnnnnnnnnnnnnnnnnn nnnnnnnnnnnnnnnnnnnn nnnnnnnnnnnnnnnnnnnn nnnnnnnnnnnnnnnnnnnn nnnnnnnnnnnnnnnnnnnn

A_AhovaSP TAGGGATAACAGCGTAATAT TTTTTGAGAGTTCATATTGA CAAAATTGTTTGCGACCTCG ATGTTGGATTAAGAAATCTT ATTGGTGCAAAAGTCAATTT

A_BhovaSP TAGGGATAACAGCGTAATAT TTTTTGAGAGTTCATATTGA CAAAAGTGTTTGCGACCTCG ATGTTGGATTAAGAAATCTT ATTGGTGCAAAAGTCAATTT

A_ignipes TAGGGATAACAGCGTAATAT CTTTTGAGAGTTCATATTGA CAAAAATGTTTGCCACCTCG ATGTTGGATTAGGAAATCTT ATTGACGCAAGAGTCAATTT

A_Bcowani nnnnnnnnnnnnnnnnnnnn nnnnnnnnnnnnnnnnnnnn nnnnnnnnnnnnnnnnnnnn nnnnnnnnnnnnnnnnnnnn nnnnnnnnnnnnnnnnnnnn

A_Ccowani TAGGGATAACAGCGTAATAT TTTTTGAGAGTTCATA-TGA CAAAAATGTTTGCGACCTCG ATG----------------- --------------------

A_Dcowani TAGGGATAACAGCGTAATAT TTTTTGAGAGTTCATATTGA CAAAAATGTTTGCGACCTCG ATGTTGGATTAAGAAATCCT ATTGGGGCAGGATTCAA-TT

A_Asangui TAGGGATAACAGCGTAATAT TTTTTGAGAGTACATAATGA CRAAAGTGTTTGCSACCTCS ATGTTGGATTAAGAAAT--- --------------------

A_Bsangui nnnnnnnnnnnnnnnnnnnn nnnnnnnnnnnnnnnnnnnn nnnnnnnnnnnnnnnnnnnn nnnnnnnnnnnnnnnnnnnn nnnnnnnnnnnnnnnnnnnn

A_Csangui nnnnnnnnnnnnnnnnnnnn nnnnnnnnnnnnnnnnnnnn nnnnnnnnnnnnnnnnnnnn nnnnnnnnnnnnnnnnnnnn nnnnnnnnnnnnnnnnnnnn

A_Dsangui TAGGGATAACAGCGTAATAT TTTTTGAGAGTTCATATTGA CTAAAGT-TTTGCGACCTCG ATGTTGGATTAAGAAATCCT ATTGACGCAGAATCA-----

Doratogon TAAAGAGGGTCTGTTCGACC TTTAAATTCTT

Mad_maxAS nnnnnnnnnnnnnnnnnnnn nnnnnnnnnnn

Mad_maxBS nnnnnnnnnnnnnnnnnnnn nnnnnnnnnnn

S_simplex AAAAGATAGTCTGTTCGACT ATTAAATTCTT

S_triareu GAGAGATAGTCTGTTCGACT ATTAAATTCTT

C_AsemiSP nnnnnnnnnnnnnnnnnnnn nnnnnnnnnnn

C_BsemiSP nnnnnnnnnnnnnnnnnnnn nnnnnnnnnnn

A_Avampyr -------------------- -----------

A_Bvampyr nnnnnnnnnnnnnnnnnnnn nnnnnnnnnnn

A_Cvampyr -------------------- -----------

A_Dvampyr nnnnnnnnnnnnnnnnnnnn nnnnnnnnnnn

A_Ainfern TAAAGATAGTCTGTTCGACT ATTAAATTCTT

A_Binfern TAAAGATAGTCTGTTCGACT ATTAAATTCTT

A_Cinfern TAAAGATAGTCTGTTCGACT ATTAAATTCTT

A_Dinfern TAAAGATAGTCTGTTCGACT ATTAAATTCTT

A_Einfern TAAAGATAGTCTGTTCGACT ATTAAATTCTT

A_Acorall TAAAGATAGTCTGTTCGACT ATTAAATTCTT

A_Bcorall TAAAGATAGTCTGTTCGACT ATTAAATTCTT

A_nsp1229 TAAAGATAGTCTGTTCGACT ATTAAATTCTT

A_nsp1396 TATAGATAGTCTGTTCGACT ATTAATTTCCT

A_Aerythr TAAAGATAGTCTGTTCGACT ATTAAATTCTT

A_Berythr nnnnnnnnnnnnnnnnnnnn nnnnnnnnnnn

A_AhovaSP TAGAGATAGTCTGTTCGACT ATTAAATTCTT

A_BhovaSP TAAAGATAGTCTGTTCGACT ATTAAATTCTT

A_ignipes TAAAGATAGTCTGTTCGACT ATTAAATTCTT

A_Bcowani nnnnnnnnnnnnnnnnnnnn nnnnnnnnnnn

A_Ccowani -------------------- -----------

A_Dcowani TAGGGATAGTCTGTTCGACT ATTAAATTCTT

A_Asangui -------------------- -----------

A_Bsangui nnnnnnnnnnnnnnnnnnnn nnnnnnnnnnn

A_Csangui nnnnnnnnnnnnnnnnnnnn nnnnnnnnnnn

A_Dsangui -------------------- -----------

;

end;
