# Supplementary material for: Mountain Refugia Play a Role in Soil Arthropod Speciation on Madagascar: A Case Study of the Endemic Giant Fire-Millipede Genus Aphistogoniulus
Source: PLoS One. 2011 Dec 6;6(12):e28035. doi: 10.1371/journal.pone.0028035 (PMC3232213; doi:10.1371/journal.pone.0028035)
Supplement: Supporting Information S8 — Specimens, voucher code and locality information of specimens used for the molecular analysis. All 31 specimens, belonging to four genera and 12 species, were newly sequenced. Abbreviations: FMNH-INS = Field Museum insect collection number; CASENT = California Academy of Sciences Entomology specimen number. (DOC) [file pone.0028035.s008.doc]

**Supporting Information S8:** Specimens, voucher code and locality information of specimens used for the molecular analysis. All 31 specimens, belonging to four genera 12 species were newly sequenced. Abbreviations of museum acronyms: FMNH-INS = Field Museum of Natural history - insect collection; CASENT = California Academy of Sciences, Entomology.

| **Species** | **Voucher code** | **locality** |
| --- | --- | --- |
| *Madabolus maximus* Wesener & Enghoff, 2008 | FMNH-INS X01 | Madagascar, pet trade |
| *Madabolus maximus* Wesener & Enghoff, 2008 | FMNH-INS X02 | Madagascar, pet trade |
| *Spiromimus simplex* Wesener & Enghoff, 2009 | CASENT 9032813 | Madagascar, Province d’Antsiranana, PN de Marojejy, Manantenina River, rainforest, 450 m, 14°26’12”S, 049°46’30”E, coll. B.L. Fisher et al., 12-15.xi.2003 |
| *Spiromimus triaureus* Wesener & Enghoff, 2009 | CASENT 9032804 | Madagascar, Province de Mahajanga, Forêt de Tsimembo, tropical dry forest, 20 m, 19°1’17”S, 44°26’26”E, coll. Fisher, Griswold et al., 21-25.xi.2001 |
| *Colossobolus semicyclus* Wesener, 2009 | CASENT 9032800 | Madagascar, Province d’Antsiranana, Forêt de Binara, rainforest, 650–800 m, 13°15’48” S, 049°36’12” E, coll. B. L. Fisher, 19.XI.2004 |
| *Colossobolus semicyclus* Wesener, 2009 | CASENT 9032801 | Madagascar, Province d’Antsiranana, Forêt de Binara, rainforest, 650–800 m, 13°15’48” S, 049°36’12” E, coll. B. L. Fisher, 19.XI.2004 |
| *Aphistogoniulus vampyrus* A  Wesener, 2009 | FMNH-INS 5387 | Madagascar, Province de Toliara, PN d’Andohahela, parcel 1, 1500 m, montane rainforest, 24°34.2’S, 46°43.9’S, coll. S. Goodman et al, 17-27.xi.1995 |
| *Aphistogoniulus vampyrus* B  Wesener, 2009 | FMNH-INS 5366 | Madagascar, Province de Toliara, PN d’Andohahela, parcel 1, 13.5 km NE Eminiminy, camp #3, 1200 m, montane rainforest, 24°35.0’S, 46°44.1’E, coll. S. Goodman, 1–17.xi.1995 |
| *Aphistogoniulus vampyrus* C  Wesener, 2009 | FMNH-INS 5392 | Madagascar, Province de Toliara, PN d’Andohahela, parcel 1, 1500 m, montane rainforest, 24°34.2’S, 46°43.9’S, coll. S. Goodman *et al.*, 17-27.xi.1995 |
| *Aphistogoniulus vampyrus* D  Wesener, 2009 | FMNH-INS 5414 | Madagascar, Province de Toliara, PN d’Andohahela, parcel 1, 1500 m, montane rainforest, 24°32.2’S, 46°43.9’E, coll. S. Goodman, 17–27.xi.1995 |
| *Aphistogoniulus infernalis* A Wesener, 2009, | FMNH-INS | Madagascar, Province de Toliara, Sainte Luce, S8, 24°46.520’S, 047°09.074’E, 28 m, littoral forest on basaltic soil, coll. Wesener et al., 29.v.2007 |
| *Aphistogoniulus infernalis* B Wesener, 2009, | FMNH-INS | Madagascar, Province de Toliara, Sainte Luce, S8, 24°46.520’S, 047°09.074’E, 28 m, littoral forest on basaltic soil, coll. Wesener et al., 29.v.2007 |
| *Aphistogoniulus infernalis* C Wesener, 2009, Isaka-Ivondro Nord | FMNH-INS-56488 | Madagascar, Province de Toliara, Andohahela, Isaka-Ivondro Nord [is this within park?], 668 m, rainforest, 24°44.185’S, 46°51.607’E, coll. Wesener et al., 12.vi.2007 |
| *Aphistogoniulus infernalis* D Wesener, 2009, Isaka-Ivondro Nord | FMNH-INS-56488 | Madagascar, Province de Toliara, Andohahela, Isaka-Ivondro Nord [is this within park?], 668 m, rainforest, 24°44.185’S, 46°51.607’E, coll. Wesener et al., 12.vi.2007 |
| *Aphistogoniulus infernalis* E Wesener, 2009 | CASENT 9032823 | Madagascar, Province de Toliara, Grand Lavasoa, 450 m, rainforest, 25˚05'16" S, 046˚44'56" E, coll. B. L. Fisher et al., 30.XI.2006 |
| *Aphistogoniulus corallipes* A (DeSaussure & Zehntner, 1902) | FMNH-INS-56116 | Madagascar, Province de Toliara, Private Reserve Manantantely, rainforest, 24°59’17.14”S, 046°55’27.95” E, coll. T. Wesener et al., 06.vi.2007 |
| *Aphistogoniulus corallipes* B (DeSaussure & Zehntner, 1902) | FMNH-INS-56116 | Madagascar, Province de Toliara, Private Reserve Manantantely, rainforest, 24°59’17.14”S, 046°55’27.95” E, coll. T. Wesener et al., 06.vi.2007 |
| *Aphistogoniulus* *jeekeli* n. sp. A | CASENT 9032790 | Madagascar, Province de Fianarantsoa, Forêt Classée de Vatovavy, rainforest, 175 m, 21°24'00" S, 47°56'24" E, coll. S. Goodman et al*.*, 6.-8.vi.2005 |
| *Aphistogoniulus* *jeekeli* n. sp. B | CASENT 9032822 | Madagascar, Province de Fianarantsoa, Réserve Speciale de Manombo, rainforest, 30 m, 23°00'57" S, 47°43'08" E, coll. Brian L. Fisher et al.*.*, 20.iv.2006. |
| *Aphistogoniulus erythrocephalus* A (Pocock, 1893) | CASENT 9032802 | Madagascar, Province d’Antsiranana, Ambondrobe, littoral rainforest, 10 m, 13°42'55" S, 050°06'06" E, coll. B. L. Fisher, 29.XI.2004 |
| *Aphistogoniulus erythrocephalus* B (Pocock, 1893) | CASENT 9032810 | Madagascar, Province d’Antsiranana, Forét d’Ambanitaza, rainforest, 240 m, 14°40'46" S, 050°11'01" E, coll. B. L. Fisher, 26.XI.2004 |
| *Aphistogoniulus hova* A (DeSaussure & Zehntner, 1897) | CASENT 9032803 | Madagascar, Province de Toamasina, Reserve de Betampona, Camp Vohitsivalana, rainforest, 520 m, 17°53'12" S, 049°12'09" E, coll. Brian L. Fisher et al., 1–3.xii.2005 |
| *Aphistogoniulus hova* B (DeSaussure & Zehntner, 1897) | CASENT 9032821 | Madagascar, Province de Toamasina, Ile Sainte Marie, Forêt de Kalalao, rainforest, 100 m, 16°55'21" S, 049°53'14" E, coll. Brian L. Fisher et al., 24–27.xi.2005 |
| *Aphistogoniulus hova* C (DeSaussure & Zehntner, 1897) | FMNH-INS 55886 | Madagascar, imported to Germany by OdenwaldExoten, most likely from: Province de Toamasina, Andasibe (Périnet), 2009 |
| *Aphistogoniulus cowani* B  (Butler, 1882) | FMNH-INS 7792 | Province de Fianarantsoa, extreme northern limit of RS d’Ivohibe, mid-elevation forest, 1200 m, 22°28.2’S, 46°57.6’E, coll. S. Goodman, 3-9.ix.1997 |
| *Aphistogoniulus cowani* C  (Butler, 1882) | FMNH-INS 7791 | Province de Fianarantsoa, extreme northern limit of RS d’Ivohibe, mid-elevation forest, 1200 m, 22°28.2’S, 46°57.6’E, coll. S. Goodman, 3-9.ix.1997, |
| *Aphistogoniulus cowani* D  (Butler, 1882) | FMNH-INS 7866 | Province de Fianarantsoa, extreme northern limit of RS d’Ivohibe, mid-elevation forest, 1200 m, 22°28.2’S, 46°57.6’E, coll. S. Goodman, 3-9.ix.1997, |
| *Aphistogoniulus sanguineus* A Wesener, 2009 | FMNH-INS 54 | Madagascar, Province d’Antananarivo, RS d’Ambohitantely, disturbed transitional montane mossy forest, 1450 m, 18°10.1’S, 47°16.6’E, coll. S. Goodman, 7 -12.xii.1997 |
| *Aphistogoniulus sanguineus* B Wesener, 2009 | FMNH-INS 7904 | Madagascar, Province d’Antananarivo, RS d’Ambohitantely, disturbed transitional montane mossy forest, 1450 m, 18°10.1’S, 47°16.6’E, coll. S. Goodman, 7 -12.xii.1997 |
| *Aphistogoniulus sanguineus* C Wesener, 2009 | FMNH-INS 44981 | Madagascar, Province d’Antananarivo, RS d’Ambohitantely, disturbed transitional montane mossy forest, 1450 m, 18°10.1’S, 47°16.6’E, coll. S. Goodman, 7 -12.xii.1997 |
| *Aphistogoniulus sanguineus* D Wesener, 2009 | FMNH-INS 7890 | Madagascar, Province d’Antananarivo, RS d’Ambohitantely, disturbed transitional montane mossy forest, 1450 m, 18°10.1’S, 47°16.6’E, coll. S. Goodman, 7 -12.xii.1997 |
