# Supplementary material for: Mountain Refugia Play a Role in Soil Arthropod Speciation on Madagascar: A Case Study of the Endemic Giant Fire-Millipede Genus Aphistogoniulus
Source: PLoS One. 2011 Dec 6;6(12):e28035. doi: 10.1371/journal.pone.0028035 (PMC3232213; doi:10.1371/journal.pone.0028035)
Supplement: Supporting Information S9 — PCR primers & Sequencing primers. (DOC) [file pone.0028035.s009.doc]

**Supporting Information 9A 2**: PCR primers

| **Gene** | **Primer** | **Primer (sequence)** | **source** |
| --- | --- | --- | --- |
| 16S rRNA | **16a** | 5’-CGCCTGTTTATCAAAAACAT-3’ | Palumbi *et al.*, 1991 |
|  | **16b** | 5’-CCGGTCTGAACTCAGATCATGT-3’ | Palumbi *et al.*, 1991 |
| COI | **HCO** | 5’-TAAACTTCAGGGTGACCAAAAAATCA-3’ | Folmer *et al.*, 1994 |
|  | **LCO** | 5’-GGTCAACAAATCATAAAGATATTGG-3’ | Folmer *et al.*, 1994 |
| 18S rRNA | **F1** | 5’-CTGGTTGATCCTGCCAGT-3’ | Giribet *et al.*, 1996 |
|  | **R1** | 5’-TATTGATCCTTCCGCAGGTTCACCT-3’ | Giribet *et al.*, 1996 |

**Supporting Information 9B**: Sequencing primers

| **Gene** | **Primer** | **Primer (sequence)** | **source** |
| --- | --- | --- | --- |
| 16S rRNA | **16a** | 5’-CGCCTGTTTATCAAAAACAT-3’ | Palumbi *et al.*, 1991 |
|  | **16b** | 5’-CCGGTCTGAACTCAGATCATGT-3’ | Palumbi *et al.*, 1991 |
| COI | **HCO-2198** | 5’-TAAACTTCAGGGTGACCAAAAAATCA-3’ | Folmer *et al.*, 1994 |
|  | **LCO-1490** | 5’-GGTCAACAAATCATAAAGATATTGG-3’ | Folmer *et al.*, 1994 |
| 18S rRNA | **F1** | 5’-CTGGTTGATCCTGCCAGT-3’ | Giribet *et al.*, 1996 |
|  | **F2** | 5’-GCCGCGGTAATTCCAGC-3’ | Raupach *et al.*, 2009 |
|  | **F3** | 5’-CGATCAGATACCGCCCTAGTTC-3’ | Raupach *et al.*, 2009 |
|  | **F4** | 5’-GGCCGTTCTTAGTTGGTGGAG-3’ | Raupach *et al.*, 2009 |
|  | **R1** | 5’-TATTGATCCTTCCGCAGGTTCACCT-3’ | Giribet *et al.*, 1996 |
|  | **R2** | 5’-CGCGGCTGCTGGCACCAGAC-3’ | Raupach *et al.*, 2009 |
|  | **R3** | 5’-CCGTCAATTCCTTTAAGTTTCAG-3’ | Raupach *et al.*, 2009 |
|  | **R4** | 5’-CATCTAGGGCATCACAGACC-3’ | Raupach *et al.*, 2009 |
