# Supplementary material for: Mountain Refugia Play a Role in Soil Arthropod Speciation on Madagascar: A Case Study of the Endemic Giant Fire-Millipede Genus Aphistogoniulus
Source: PLoS One. 2011 Dec 6;6(12):e28035. doi: 10.1371/journal.pone.0028035 (PMC3232213; doi:10.1371/journal.pone.0028035)
Supplement: Supporting Information S11 — Result file of the Bayesian analysis using RASP. (DOC) [file pone.0028035.s011.doc]

**Supporting Information S11:** Result file of the Bayesian analysis using RASP.

**Bayesian Analysis result file**

**[TAXON]**

**1 Colossobolus_semicyclus_Seasonal_Dry_Forest A**

**2 Aphistogoniulus_cowani_Montane_Rainforest B**

**3 A_sanguineus_Montane_Rainforest B**

**4 A_erythrocephalus_Lowland/Mid_elevation Rainforest CD**

**5 A_hova_Lowland/Mid_elevation_Rainforest CD**

**6 A_corallipes_Lowland_Rainforest C**

**7 A_jeekeli_Lowland_Rainforest C**

**8 A_infernalis_Lowland_Rainforest C**

**9 A_vampyrus_Mid_elevation_Rainforest D**

**[TREE]**

**Tree=(1,(((2,3),(4,5)),(7,(6,(8,9)))));**

**[RESULT]**

**result of combined:**

**node 10 (anc. of terminals 2-3): B 96.41 BC 2.33 C 0.53 BD 0.43 AB 0.15 D 0.10 A 0.03 BCD 0.01 ABC 0.00 CD 0.00 AC 0.00 ABD 0.00 AD 0.00 ABCD 0.00 ACD 0.00**

**node 11 (anc. of terminals 4-5): CD 84.26 C 13.44 BCD 1.31 D 0.73 BC 0.21 ACD 0.03 BD 0.01 AC 0.01 B 0.00 ABCD 0.00 AD 0.00 ABC 0.00 A 0.00 ABD 0.00 AB 0.00**

**node 12 (anc. of terminals 2-5): C 63.61 CD 10.20 D 8.98 BC 7.64 B 6.72 BCD 1.22 BD 1.08 AC 0.22 A 0.20 ACD 0.04 AD 0.03 ABC 0.03 AB 0.02 ABCD 0.00 ABD 0.00**

**node 13 (anc. of terminals 8-9): C 96.92 CD 2.47 D 0.37 BC 0.12 AC 0.07 B 0.02 A 0.01 BCD 0.00 ACD 0.00 BD 0.00 AD 0.00 ABC 0.00 AB 0.00 ABCD 0.00 ABD 0.00**

**node 14 (anc. of terminals 6-9): C 99.35 CD 0.43 BC 0.15 AC 0.05 D 0.02 B 0.01 A 0.00 BCD 0.00 ACD 0.00 ABC 0.00 BD 0.00 AD 0.00 AB 0.00 ABCD 0.00 ABD 0.00**

**node 15 (anc. of terminals 7-9): C 99.06 BC 0.59 CD 0.20 AC 0.07 B 0.06 D 0.02 A 0.01 BCD 0.00 ABC 0.00 ACD 0.00 BD 0.00 AB 0.00 AD 0.00 ABCD 0.00 ABD 0.00**

**node 16 (anc. of terminals 2-9): C 90.19 B 2.46 BC 2.20 D 1.81 CD 1.62 A 0.83 AC 0.74 BD 0.04 BCD 0.04 AB 0.02 ABC 0.02 AD 0.01 ACD 0.01 ABD 0.00 ABCD 0.00**

**node 17 (anc. of terminals 1-9): A 55.50 C 35.06 AC 3.38 B 2.84 D 2.35 AB 0.27 AD 0.23 BC 0.17 CD 0.14 ABC 0.02 ACD 0.01 BD 0.01 ABD 0.00 BCD 0.00 ABCD 0.00**

**result of run 1:**

**node 10 (anc. of terminals 2-3): B 96.61 BC 2.23 C 0.48 BD 0.39 AB 0.16 D 0.08 A 0.03 BCD 0.01 ABC 0.00 CD 0.00 AC 0.00 ABD 0.00 AD 0.00 ABCD 0.00 ACD 0.00**

**node 11 (anc. of terminals 4-5): CD 83.99 C 13.52 BCD 1.39 D 0.82 BC 0.22 ACD 0.04 BD 0.01 AC 0.01 B 0.00 ABCD 0.00 AD 0.00 ABC 0.00 A 0.00 ABD 0.00 AB 0.00**

**node 12 (anc. of terminals 2-5): C 63.01 CD 10.57 D 9.12 BC 7.68 B 6.63 BCD 1.29 BD 1.11 AC 0.25 A 0.21 ACD 0.04 AD 0.04 ABC 0.03 AB 0.03 ABCD 0.01 ABD 0.00**

**node 13 (anc. of terminals 8-9): C 96.49 CD 2.83 D 0.44 BC 0.13 AC 0.07 B 0.02 A 0.01 BCD 0.00 ACD 0.00 BD 0.00 AD 0.00 ABC 0.00 AB 0.00 ABCD 0.00 ABD 0.00**

**node 14 (anc. of terminals 6-9): C 99.32 CD 0.45 BC 0.15 AC 0.05 D 0.02 B 0.01 A 0.00 BCD 0.00 ACD 0.00 ABC 0.00 BD 0.00 AD 0.00 AB 0.00 ABCD 0.00 ABD 0.00**

**node 15 (anc. of terminals 7-9): C 99.18 BC 0.49 CD 0.19 AC 0.07 B 0.05 D 0.02 A 0.01 BCD 0.00 ABC 0.00 ACD 0.00 BD 0.00 AB 0.00 AD 0.00 ABCD 0.00 ABD 0.00**

**node 16 (anc. of terminals 2-9): C 89.91 B 2.56 BC 2.35 D 1.72 CD 1.59 A 0.89 AC 0.82 BD 0.05 BCD 0.04 AB 0.02 ABC 0.02 AD 0.02 ACD 0.01 ABD 0.00 ABCD 0.00**

**node 17 (anc. of terminals 1-9): A 55.53 C 35.12 AC 3.54 B 2.76 D 2.19 AB 0.28 AD 0.22 BC 0.18 CD 0.14 ABC 0.02 ACD 0.01 BD 0.01 ABD 0.00 BCD 0.00 ABCD 0.00**

**result of run 2:**

**node 10 (anc. of terminals 2-3): B 96.21 BC 2.44 C 0.59 BD 0.47 AB 0.13 D 0.11 A 0.03 BCD 0.01 ABC 0.00 CD 0.00 AC 0.00 ABD 0.00 AD 0.00 ABCD 0.00 ACD 0.00**

**node 11 (anc. of terminals 4-5): CD 84.53 C 13.36 BCD 1.22 D 0.64 BC 0.19 ACD 0.03 BD 0.01 AC 0.00 B 0.00 ABCD 0.00 AD 0.00 ABC 0.00 A 0.00 ABD 0.00 AB 0.00**

**node 12 (anc. of terminals 2-5): C 64.23 CD 9.83 D 8.83 BC 7.59 B 6.82 BCD 1.16 BD 1.04 AC 0.20 A 0.18 ACD 0.03 AD 0.03 ABC 0.02 AB 0.02 ABCD 0.00 ABD 0.00**

**node 13 (anc. of terminals 8-9): C 97.36 CD 2.11 D 0.31 BC 0.12 AC 0.07 B 0.02 A 0.01 BCD 0.00 ACD 0.00 BD 0.00 AD 0.00 ABC 0.00 AB 0.00 ABCD 0.00 ABD 0.00**

**node 14 (anc. of terminals 6-9): C 99.39 CD 0.41 BC 0.14 AC 0.04 D 0.02 B 0.01 A 0.00 BCD 0.00 ACD 0.00 ABC 0.00 BD 0.00 AD 0.00 AB 0.00 ABCD 0.00 ABD 0.00**

**node 15 (anc. of terminals 7-9): C 98.93 BC 0.69 CD 0.20 B 0.08 AC 0.07 D 0.02 A 0.01 BCD 0.00 ABC 0.00 BD 0.00 ACD 0.00 AB 0.00 AD 0.00 ABCD 0.00 ABD 0.00**

**node 16 (anc. of terminals 2-9): C 90.47 B 2.35 BC 2.04 D 1.91 CD 1.66 A 0.76 AC 0.66 BD 0.04 BCD 0.04 AB 0.02 ABC 0.02 AD 0.01 ACD 0.01 ABD 0.00 ABCD 0.00**

**node 17 (anc. of terminals 1-9): A 55.47 C 34.98 AC 3.22 B 2.94 D 2.53 AB 0.27 AD 0.23 BC 0.17 CD 0.15 ABC 0.02 ACD 0.01 BD 0.01 ABD 0.00 BCD 0.00 ABCD 0.00**
